# Taping for conditions of the musculoskeletal system: an evidence map review

**DOI:** 10.1186/s12998-020-00337-2

**Published:** 2020-09-15

**Authors:** Zachary A. Cupler, Muhammad Alrwaily, Emily Polakowski, Kevin S. Mathers, Michael J. Schneider

**Affiliations:** 1 Physical Medicine & Rehabilitation Services, Butler VA Healthcare System, 353 N. Duffy Road, Butler, Pennsylvania USA; 2 Division of Physical Therapy, School of Medicine, West Virginia University, Morgantown, West Virginia USA; 3 Department of Physical Therapy, King Fahad Specialist Hospital, Dammam, Saudi Arabia; 4 Private Practice, Independence Physical Therapy, Mystic, Connecticut USA; 5 VA Pittsburgh Healthcare System, Pittsburgh, Pennsylvania USA; 6 Department of Physical Therapy, School of Health and Rehabilitation Sciences, University of Pittsburgh, Pittsburgh, Pennsylvania USA

**Keywords:** Taping, Musculoskeletal, Evidence map

## Abstract

**Background:**

Taping is a common treatment modality used by many rehabilitation providers. Several types of tapes and taping methods are used in the treatment of musculoskeletal dysfunction and pain.

**Purpose:**

To summarize and map the evidence related to taping methods used for various joints and conditions of the musculoskeletal system, and to provide clinicians and researchers with a user-friendly reference with organized evidence tables.

**Data sources:**

The PEDro, CINAHL, Cochrane Database of Systematic Reviews, Cochrane Central Register for Controlled Trials, PubMed, and PROSPERO databases were searched from inception through October 31, 2019.

**Study selection:**

Eligible studies were selected by two independent reviewers and included either systematic reviews (SRs) or randomized controlled trials (RCTs) and included a musculoskeletal complaint using a clinical outcome measure.

**Data extraction:**

Data was extracted by two investigators independently. Risk of bias and quality were assessed using A MeaSurement Tool to Assess systematic Reviews (AMSTAR) for SRs or the Physiotherapy Evidence Database (PEDro) scale for RCTs. The protocol was registered with PROSPERO (CRD42019122857).

**Data synthesis:**

Twenty-five musculoskeletal conditions were summarized from forty-one SRs and 127 RCTs. There were 6 SRs and 49 RCTs for spinal conditions. Kinesio tape was the most common type of tape considered. Four evidence tables representing the synthesized SRs and RCTs were produced and organized by body region per condition.

**Limitations:**

Inclusion of only English language studies. Also, the heterogeneous nature of the included studies prevented a meta-analysis.

**Conclusions:**

There is mixed quality evidence of effectiveness for the different types of taping methods for different body regions and conditions. All of the SRs and RCTs found during our search of the taping literature have been organized into a series of appendices. A synthesis of the results have been placed in evidence tables that may serve as a useful guide to clinicians and researchers.

## Introduction

### Description of the condition

Musculoskeletal conditions are reported by approximately one in two adults in the United States, and are considered amongst the top five medical conditions reported in 2015 [1]. These conditions are a major contributor to disability, accounting for 17.1% of years lived with disability [1]. The disability and reduced quality of life resulting from these conditions are reported to be at much higher rates than hypertension and elevated cholesterol levels [1]. The major contributors to this disability are low back pain, neck pain, [2] hip and knee osteoarthritis [3], shoulder complex conditions amongst other joints [4]. Collectively, these conditions are commonly seen in rehabilitation clinics and taping is a very popular modality used in their management.

### Description of the intervention

The use of taping as a treatment method has been reported in the literature as early as 1969 [5]. Taping is used to treat acute and chronic musculoskeletal complaints including, but not limited to, pain, function, joint instability, and edema [6]. Prophylactic use of tape to prevent sporting injuries has also been reported [5, 7, 8]. At the 2012 Olympic games in London, taping was one of the five most frequently used treatment modalities, accounting for 8.9% of used interventions [9]. Multiple methods of taping are available, including rigid taping, Kinesio tape, McConnell and Mulligan. Each of these types of taping has been associated with specific therapeutic mechanisms that we detail below.

#### Rigid (athletic) taping

Rigid taping is a tan-colored adhesive strap that is used primarily by athletes to prevent or reduce the chance of injury. Rigid tape, such as leukotape, is frequently applied in a standardized protocol for each joint. The application begins with an under-wrap to reduce skin irritation, with rigid tape applied over the under-wrap on the area of injury. For each joint, a rigid tape protocol is a pattern of multiple straps applied to the body part to secure the joint in a specific position while allowing the individual to participate in the athletic activity. A specific sub-type of rigid taping for abnormal pronation is described in the literature at times as “low dye tape” [6]. This method of taping is used for purposes such as reducing pain, providing proprioceptive feedback during activity, and limiting excessive joint motion [10].

#### Kinesio taping

Kinesio tape (KT) was developed in Japan by Kenzo Kase, a chiropractor and acupuncturist, as an alternative tape and methodology to the traditional rigid taping materials and methods [11]. After the dramatic increase in its usage during the Olympics, KT has become a popular taping method in daily life, sports, as well as by various health care practitioners [11]. KT is an adhesive elastic tape that comes in numerous colors and patterns that is applied directly to the skin without the need for under-wrap. KT is designed to mimic the elasticity of the skin. Theoretically, the KT is presumed to target different receptors within the somatosensory system with the intent to lessen pain and promote lymphatic drainage by lifting the skin to increase interstitial space [12]. Numerous trials and systematic reviews have been conducted to investigate the mechanisms of KT [7, 8, 13–17].

#### McConnell taping

McConnell taping (Mc-T) is a method pioneered in 1984, by Jenny McConnell, an Australian physical therapist [18]. This method uses a more rigid, adhesive type of tape similar to the traditional athletic tape. The Mc-T is primarily advocated to help with alignment of the patella in patients with patellofemoral pain [19], however, the application of Mc-T has been described for other joints, such as the shoulder and hip [6]. A common concern in individuals with patellofemoral pain is that the pain is presumed to be due to incorrect tracking of the patella on the femur. This tape purports to address these problems by attempting to realign the tracking of the knee. This taping method provides proprioception and mechanical pressure on the patella in one specific direction so that the patella can move freely without contacting other parts of the femur during joint articulation and thus reducing pain [19–21].

#### Mulligan taping

Mulligan taping is an adhesive strap method similar to rigid taping. This tape is used as a supplement to the Mulligan mobilization with movement procedure, a therapeutic technique using manual force that is applied to a joint and sustained in specific direction. The manual therapy technique of Mulligan is intended to allow painless motion of a previously painful joint [22]. After the Mulligan mobilization is conducted, rigid tape can be applied to the body region in the same direction as the manual force. This is thought to extend the benefit of the mobilization with movement after the treatment session ends [23].

### Why this evidence map is important

There are numerous systematic reviews (SRs) and randomized controlled trials (RCTs) that have been conducted on the effectiveness of taping on various body regions and musculoskeletal conditions. These studies have included different types of taping methods, varied outcome measures, diverse patient populations for different body regions, and sometimes yield contradictory conclusions. Because of this variety of methods and measures, it has become difficult for clinicians and researchers to appraise evidence about taping effectiveness. Therefore, this evidence map sought to comprehensively summarize, synthesize, and organize the abundant literature about the effectiveness of various taping methods on the musculoskeletal system. This evidence mapping seeks to develop a concise, and comprehensive clinic-friendly tool that summarizes the evidence regarding the effectiveness of taping for the musculoskeletal system while also identifying gaps in knowledge and guide future research [24].

## Methods

Evidence mapping is a variant form of a systematic review. The methodology involved for this evidence map begins with a systematic literature search that shares many characteristics of a systematic review, such as defined search date terms, study selection, inclusion and exclusion parameters, data extraction, bias/quality assessment, data synthesis, and study conclusions. Evidence maps, as compared to systematic reviews, were most commonly defined as reviews of evidence to identify research gaps in addition to producing user-friendly end products such as clinical tools [24]. This study was registered in PROSPERO (CRD42019122857).

### Study selection

#### Types of studies

We included SRs from December 31, 2007 to October 31, 2019 and RCTs from inception to October 31, 2019.

#### Type of participants

We included studies that have participants aged 18 years or older presenting with clinical musculoskeletal complaints.

#### Inclusion/exclusion criteria

We included all studies that investigated any type of taping on musculoskeletal conditions. We excluded studies involving only asymptomatic participants, reporting no clinical outcome measures, reporting results limited to less than 1 day following intervention, or manuscripts was reported in a language other than English.

#### Interventions

We included all SRs and RCTs that involved a taping method as an intervention, without restriction to any specific type of tape.

#### Types of outcome measures

We included studies that involved clinically-relevant outcome measures such as pain intensity, disability, range of motion, and muscle strength, etc. We excluded studies that only reported biomechanical observations or basic science outcome measures such as lab-specific measures (e.g. EMG activity, proprioception, biomarkers, etc.)

### Data sources and searches

*Electronic search*: The keywords used to perform our search were informed by current SRs of the taping literature, and included these search terms: “Tape” OR “tapes” OR “taping” OR “strap” OR “strapping”. We searched the following databases: Physiotherapy Evidence Database (PEDro), Cumulative Index to Nursing and Allied Health Literature (CINAHL), Cochrane Database of Systematic Reviews, Cochrane Central Register for Controlled Trials (CENTRAL), PubMed, and PROSPERO. *Searching other resources:* To be more comprehensive, we hand searched the evidence tables within all included SRs for relevant RCTs that may have been missed in our electronic search.

### Data selection, extraction, and quality assessment

Two reviewers (MA, ZC) independently selected potentially relevant SRs and RCTs based first on the title, followed secondly by a review of the abstract. We used consensus to resolve disagreements concerning selection and inclusion of SRs and RCTs. Whenever disagreement persisted, a third reviewer (MS) was consulted to adjudicate the disagreement. We evaluated manuscripts that could be retrieved in full-text format and were published in English.

Two reviewers (MA, ZC) independently extracted the data using a standardized table format for all included SRs and RCTs. We used consensus to resolve discrepancies concerning data extraction. Whenever disagreement persisted, a third reviewer (MS) was consulted for adjudication. The data extracted from SRs included: author, year of publication, title, population studied, search dates, number of included studies, intervention, summary of results, and A MeaSurement Tool to Assess systematic Reviews (AMSTAR) rating. The data extracted from RCTs included: author, year of publication, title, population studied, sample size, intervention groups, outcomes time points, results, key author conclusion, and PEDro quality scores.

#### Assessment of risk of bias and quality in included studies

For SRs, we had two reviewers (KM, MA) utilize the AMSTAR rating criteria to assess the methodological quality of SRs. For RCTs, we extracted quality ratings from PEDro whenever available. If an RCT rating was not available, then we had two reviewers (MA, ZC) independently assess the quality using the PEDro scale criteria. We used consensus for rating SRs and RCTs. If disagreement persisted during assessment, a third reviewer (MS) was consulted for adjudication.

### Data synthesis and analysis

With the busy clinician in mind, we created evidence tables to provide an efficient clinician-friendly way to view the taping literature. We were unable to make our recommendations using the identified SRs because they frequently included contradictory statements made from shared or overlapping RCTs. Rather, we synthesized our recommendations from the RCTs that are retrieved from our database search, and from the RCTs identified within each SRs. We sorted the RCTs for each respective condition, and then we examined the results, conclusions and PEDro scores to make our recommendations.

The PEDro scale is a 10-item tool that provides the user a rapid way to determine internal validity and quality to guide clinical decision-making [25]. Key characteristics considered in the grading schema include: randomized allocation, group baselines, blinding, attainment of primary outcome for 85% of subjects initially allocated, intention to treat, between group comparisons, point measurements, and measurements of variability for at least one outcome. Previous work has suggested an RCT scoring a minimum of 6/10 is considered to be of ‘moderate to high quality’ [26]. To further enhance clinical efficiency, we consolidated PEDro grading to 3 levels: strong, moderate, and weak (see below).

#### Grading of the evidence

The evidence grades were trichotomized into 3 levels of strength: strong, moderate, and low (Table 1). For each level of strength, we used the following criteria:

**Table 1:**
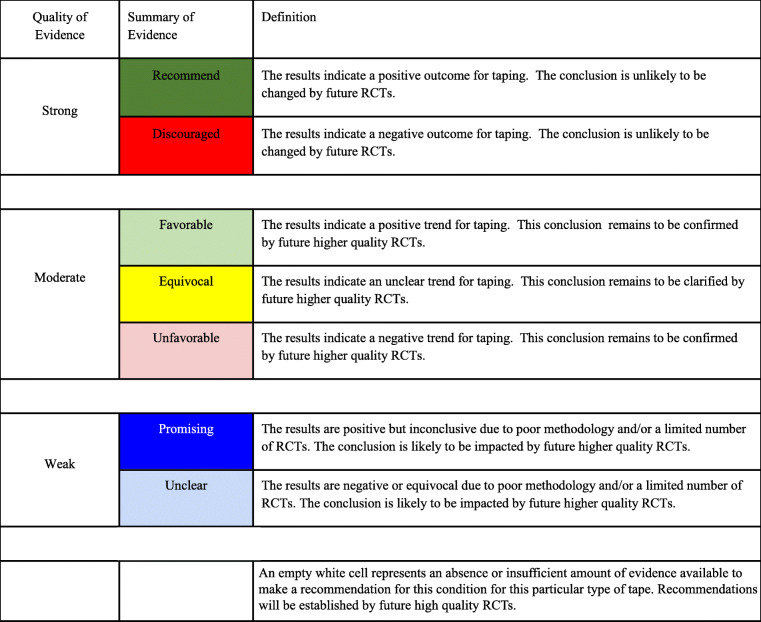
Key to Evidence Summary Tables

#### Strong level evidence

To achieve this evidence rating, the results must stem from preponderance of high-quality RCTs (PEDro 7 - 10). This grade of evidence includes well-designed, well-conducted studies with representative populations. The studies’ conclusions must be in reasonable agreement. The conclusions drawn from a strong level of evidence are considered unlikely to be changed by the results of future RCTs unless improved research methodologies, including sham and choice of control interventions, are incorporated into future studies.

#### Moderate level evidence

To achieve this evidence rating, the results should be based on at least one higher-quality RCT; or a preponderance of higher-quality RCTs with inconsistent conclusions; or a preponderance moderate-quality RCTs (PEDro 4 - 6) with harmonious results. The conclusions from moderate level of evidence are likely to be changed by the results of future, more rigorous RCTs.

#### Weak level evidence

To be at this level of evidence, the results are based on at least one moderate-quality RCT; or a preponderance of moderate-quality RCTs with inconsistent conclusions; or a preponderance of low-quality RCTs (PEDro 0 - 3). The conclusion is insufficient to identify effectiveness for conditions studied.

## Results

**Fig. 1:**
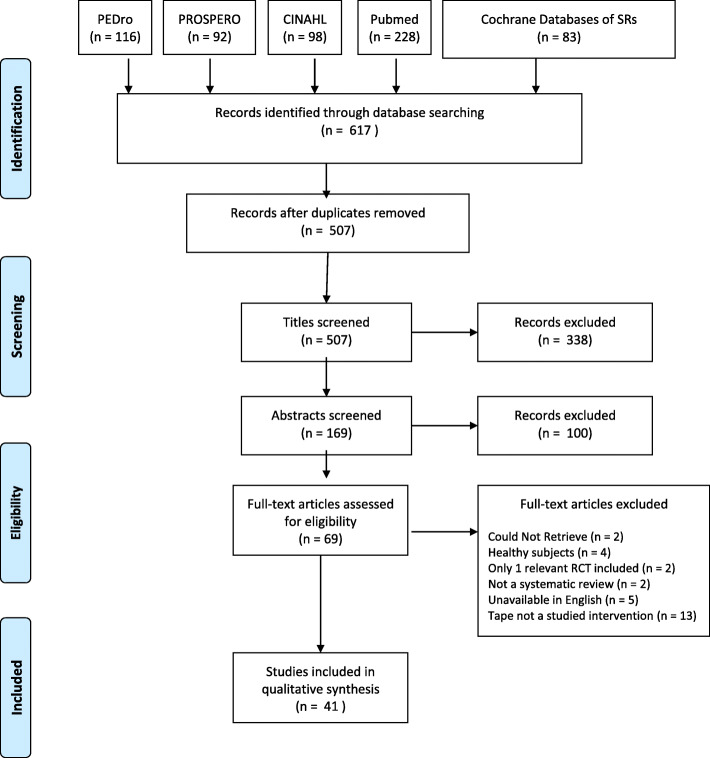
Flow diagram for systematic review selection

**Fig. 2:**
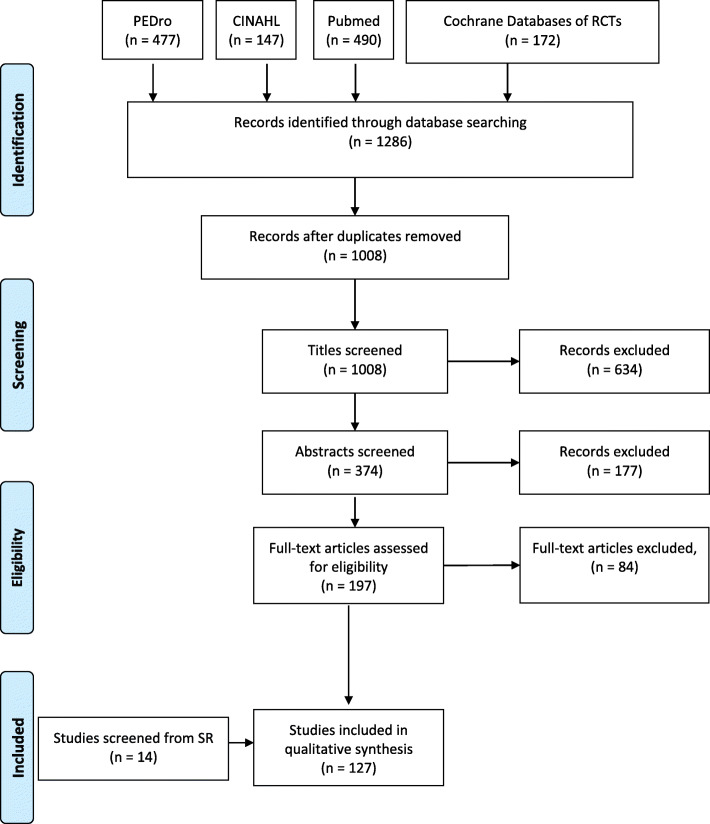
Flow diagram for randomized controlled trial selection

The literature search process for SRs is summarized in Fig. 1, and for RCTs in Fig. 2. Our search identified 41 SRs and 127 RCTs for inclusion, with a total of 25 musculoskeletal conditions summarized. The SRs were arranged in evidence tables and organized by body region: lower extremity, upper extremity and spinal conditions, respectively (Appendix 1 File). The majority of the SRs (22/41) discussed musculoskeletal conditions related to the lower extremity (Table 2).

**Table 2:** Distribution of included articles by region: Systematic Reviews

SR by region	
**Lower extremity**:	**22**
Hip	0
Knee	9
Ankle	7
Foot	6
**Upper extremity**:	**10**
Shoulder	8
Elbow	1
Hand/Wrist	1
**Spine**:	**6**
Lumbar	5
Thoracic	0
Cervical	1
TMJ	0
**Not otherwise classified**	**4**

The RCTs were also arranged in evidence tables and organized by body region: lower extremity, upper extremity, spinal conditions, and “not otherwise classified” (Table 3). Due to the plethora of available RCTs, we further subdivided each body region into its respective joints: knee, ankle, foot, shoulder, elbow and hand/wrist, lumbar spine, thoracic spine, cervical spine, and temporomandibular joint (Appendix 2 File).

**Table 3:** Distribution of included articles by region: Randomized Controlled Trials

RCT by region	
**Lower extremity**:	**42**
Hip	0
Knee	27
Ankle	8
Foot	7
**Upper extremity**:	**33**
Shoulder	19
Elbow	7
Hand/Wrist	7
**Spine**:	**49**
Lumbar	23
Thoracic	2
Cervical	20
TMJ	4
**Not otherwise classified**:	**3**

In the following section, we will detail the findings of the SRs and RCTs for each body region.

### Lower extremity conditions

Twenty-two of the total 41 SRs were related to the lower extremity conditions. The SRs of the lower extremity had an overall AMSTAR score that ranged between 2.5 to 10 (out of a maximum score of 11). Forty-two of the total 127 (33.6%) RCTs were related to lower extremity conditions. The RCTs of the lower extremities had an overall PEDro score that ranged between 1 to 8 (out of a maximum score of 10). Six different lower extremity conditions were identified and evaluated for taping procedures.

#### Hip

There were no SRs or RCTs identified about the hip that met the inclusion criteria.

#### Knee

There were 9 SRs related to the knee or patellofemoral joints (Appendix 1 File: Appendix 1A), with AMSTAR scores ranging from 4 to 10. Seven of the SRs studied taping strategies related to patellofemoral pain syndrome (PFPS) or anterior knee pain [18, 27–32]; one SR studied rigid taping for knee osteoarthritis (OA) [33], and one SR studied elastic taping for knee OA [34]. There were 27 RCTs related to PFPS [35–42], knee osteoarthritis [43–59], patellar dislocation [60], and tibial stress syndrome [61] (Appendix 2 File: Appendix 2A). The overall PEDro scores for these RCTs ranged from 4 to 8.

##### Patellofemoral pain syndrome (PFPS)

Eight studies investigated the effect of taping on PFPS. Two RCTs utilized KT and six RCTs utilized Mc-T.

##### Evidence summary (Table 4)

- There is moderate evidence that the inclusion of KT in the treatment plan of PFPS is equivocal.
- There is moderate evidence that the inclusion of Mc-T in the treatment plan of PFPS is equivocal.

**Table 4:**
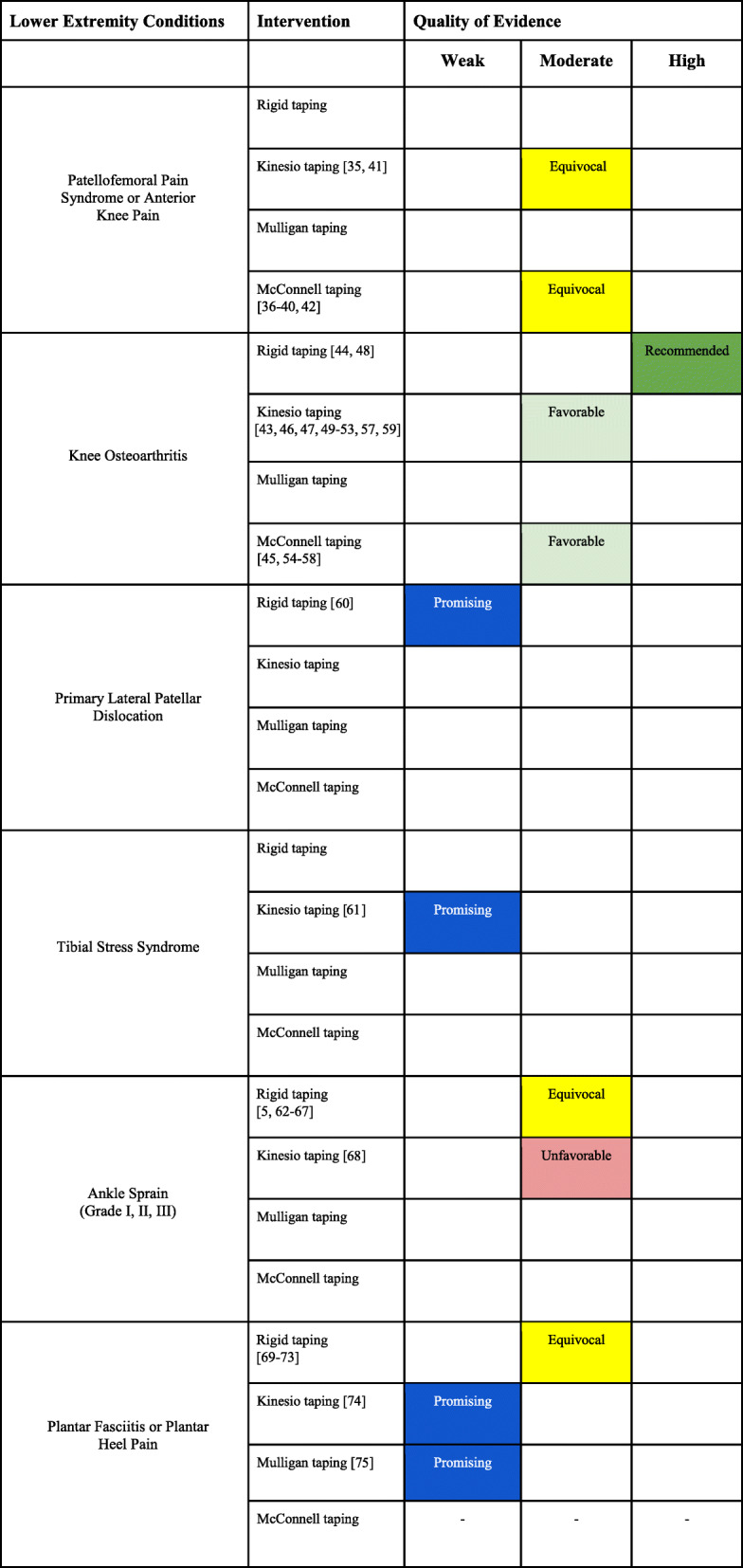
Lower extremity conditions interpretation of the evidence

##### Knee osteoarthritis (OA)

There were seventeen studies that investigated the effect of taping on knee OA. Two RCTs utilized rigid taping, ten RCTs utilized KT and five RCTs utilized Mc-T.

##### Evidence summary (Table 4)

- There is strong evidence that rigid taping is a useful adjunctive treatment in the management of pain and function in the short-term for patients with knee OA.
- There is moderate evidence that the inclusion of KT in the treatment of knee OA is favorable.
- There is moderate evidence that Mc-T is favorable in the treatment of pain and function for knee OA.

##### Primary lateral patellar dislocation

There was one RCT that investigated the effects of rigid taping for lateral patellar dislocation with moderate risk of bias.

##### Evidence summary (Table 4)

- There is promising weak evidence that rigid taping is superior to cast immobilization for recurrence of lateral patellar dislocation.

##### Tibial stress syndrome

There was one RCT that investigated the effects of KT for shin splints with high risk of bias.

##### Evidence summary (Table 4)

- There is promising weak evidence that KT is superior to orthotics for the management of tibial stress syndrome with respect to pain and function.

#### Ankle

There were seven SRs related to the ankle or talocrural joint (Appendix 1 File: Appendix 1B) with AMSTAR scores ranging from 2.5 to 9. All of the SRs investigated taping strategies to treat ankle sprains [7, 76–81]. There were eight RCTs related to ankle sprains [5, 62–68]. The overall PEDro scores ranged from 1 to 8.

##### Ankle sprains

Seven RCTs utilized rigid taping and one RCT utilized KT (Appendix 2 File: Appendix 2B). No studies investigated Mulligan taping or Mc-T for ankle sprains.

##### Evidence summary (Table 4)

- There is moderate evidence that the inclusion of rigid taping in the treatment plan of grade II and grade III ankle sprains is equivocal.
- There is moderate evidence that the inclusion of KT in the treatment plan of grade II and grade III ankle sprains is unfavorable.

#### Foot

There were six SRs related to the foot excluding the ankle or talocrural joint (Appendix 1 File: Appendix 1C), with AMSTAR scores ranging from 5 to 10. Three of the SRs assessed taping strategies to treat plantar heel pain and plantar fasciitis [82–84] and three SRs investigated taping for treatment of excessive foot pronation [85–87]. There were seven RCTs related to plantar fasciitis and plantar heel pain (Appendix 2 File: Appendix 2C). Five RCTs investigated rigid taping [69–73], one RCT investigated KT [74] and one RCT investigated Mulligan taping [75]. No study investigated Mc-T. The overall PEDro scores ranged from 3 to 7.

##### Plantar heel pain

Evidence summary (Table 4)

- There is moderate evidence that the inclusion of rigid taping in the treatment of plantar fasciitis or heel pain is equivocal.
- There is promising weak evidence that KT taping may provide adjunctive benefit to multimodal conservative treatment for plantar fasciitis or heel pain.
- There is promising weak evidence that Mulligan taping may provide adjunctive benefit to multimodal conservative treatment for plantar fasciitis or heel pain.

### Upper extremity conditions

Of the 41 SRs, ten were related to the upper extremities conditions with overall AMSTAR scores that ranged between 6 to 10. Of the 127 RCTs, 33 (26.0%) were related to the upper extremities conditions with overall PEDro scores that ranged between 3 to 9. Six conditions of the upper extremity were identified and evaluated for taping procedures.

#### Shoulder

There were eight SRs related to the shoulder, scapula, and glenohumeral joint (Appendix 1 File: Appendix 1D) with overall AMSTAR scores ranging from 6-10. All SRs evaluated taping strategies for subacromial impingement syndrome or rotator cuff tendinopathy [88–95]. There were nineteen RCTs investigating taping strategies for the treatment of SIS (Appendix 2 File: Appendix 2D). Three RCTs investigated rigid taping [96–98], fifteen RCTs investigated KT [99–113] and one RCT investigated Mulligan taping [114]. No study investigated Mc-T for SIS. The overall PEDro scores ranged from 4 to 9.

##### Subacromial impingement syndrome (SIS)

Evidence summary (Table 5)

- There is moderate evidence that rigid taping provides additional improvement to exercise and manual therapy for the treatment of SIS conditions.
- There is moderate evidence that the inclusion of KT in the treatment plan of SIS is equivocal.
- There is promising weak evidence that Mulligan taping adds benefit to manual therapy in the treatment of SIS conditions.

**Table 5:**
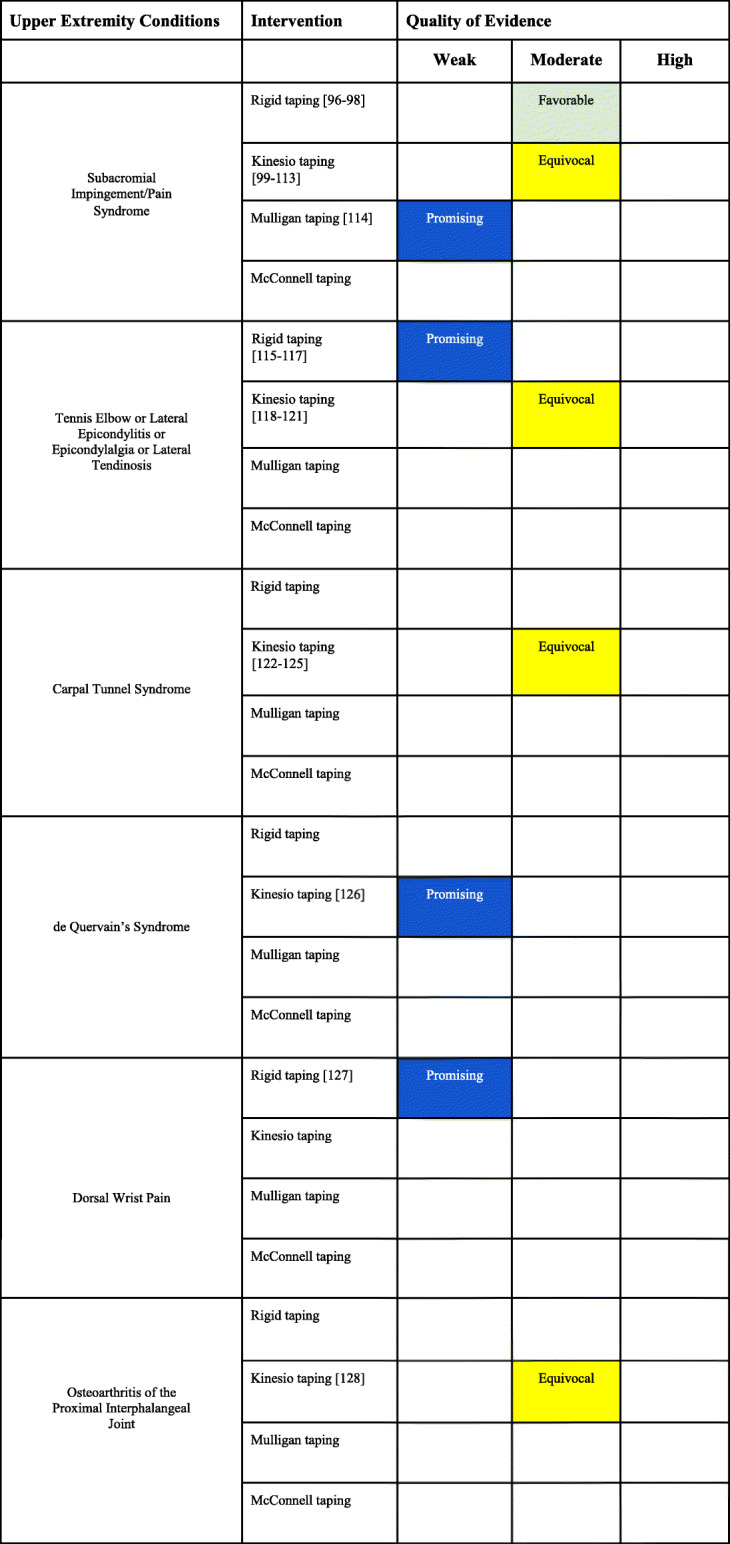
Upper extremity conditions interpretation of the evidence

#### Elbow

There was one SR of taping for lateral epicondylalgia (Appendix 1 File: Appendix 1E) with an AMSTAR rating of 9 [129]. There were seven RCTs studying the effects of taping for conditions of the elbow, humeroulnar joint, humeroradial joint or proximal radioulnar joint (Appendix 2 File: Appendix 2E) with PEDros score that ranged from 3 to 7. All RCTs studied tennis elbow or lateral epicondylalgia with three studies evaluating the effect of rigid taping [115–117] and four studies evaluating the effect of KT [118–121]. No study investigated Mulligan taping or Mc-T for lateral epicondylalgia.

##### Lateral epicondylalgia

Evidence summary (Table 5)

- There is promising weak evidence that rigid taping is a useful adjunct to physical therapy for pain or disability in the treatment of lateral epicondylalgia.
- There is moderate evidence that the use of KT as adjunct to physical therapy for pain or disability in the treatment of lateral epicondylalgia is equivocal.

#### Hand and wrist

There was one SR related to the wrist and hand, (Appendix 1 File: Appendix 1F) with an AMSTAR score of 8 [130]. There were seven RCTs investigating taping strategies for the treatment of conditions of the hand and wrist with PEDro scores ranging from 5 to 9 (Appendix 2 File: Appendix 2F). Four RCTs investigated KT for carpal tunnel syndrome [122–125], one RCT assessed KT for de Quervain’s syndrome [126], one RCT investigated rigid tape for dorsal wrist pain [127], and one RCT evaluated elastic tape configuration for OA of the proximal interphalangeal joint [128]. No studies investigated Mulligan taping or Mc-T musculoskeletal conditions of the wrist or hand.

##### Carpal tunnel syndrome

Evidence summary (Table 5)

- There is moderate evidence that the use of KT in the treatment of pain and disability for carpal tunnel syndrome is equivocal.

##### de Quervain’s syndrome

Evidence summary (Table 5)

- There is promising weak evidence that KT provides benefits to improve pain or swelling in the treatment of de Quervain’s syndrome.

##### Wrist pain

Evidence summary (Table 5)

- There is promising weak evidence that rigid tape provides benefit to improve pain and function in the treatment of dorsal wrist pain.

Osteoarthritis (OA) of the Proximal Interphalangeal Joint

##### Evidence summary (Table 5)

- There is moderate evidence that KT to improve pain or functional improvement in the treatment of OA of the proximal interphalangeal joint is equivocal.

### Conditions of the spine

Of the 41 SRs, six (14.6%) were related to spinal conditions with overall AMSTAR scores that ranged between 4 to 8. Of the 127 RCTs, 49 (38.6%) were related to spinal conditions including temporomandibular joint dysfunction and pain with overall PEDRO scores that ranged between 3 to 9. Eleven conditions of the lower extremity were identified and evaluated for taping procedures.

#### Lumbar spine

There were five SRs that included conditions related to the lumbar spine (Appendix 1 File: Appendix 1G) [131–135]. There were twenty-three RCTs related to conditions of the lumbar spine and abdominal wall, with all but one assessing KT (Appendix 2 File: Appendix 2G). One addressed KT for acute low back pain [136], two investigated KT for back pain in the setting of lumbar disc herniation [137, 138], two investigated KT for pregnancy related low back pain [139, 140], one RCT investigated postpartum diastasis recti abdominis [141], and sixteen investigated KT for chronic low back pain, [142–157]. One study evaluated rigid taping for sacroiliac joint dysfunction [158]. The overall PEDro scores ranged from 3 to 9.

##### Acute low Back pain

Evidence summary (Table 6)

- There is moderate quality evidence that KT provides adjunctive benefit to minimal care for pain control for the treatment of acute low back pain.

**Table 6:**
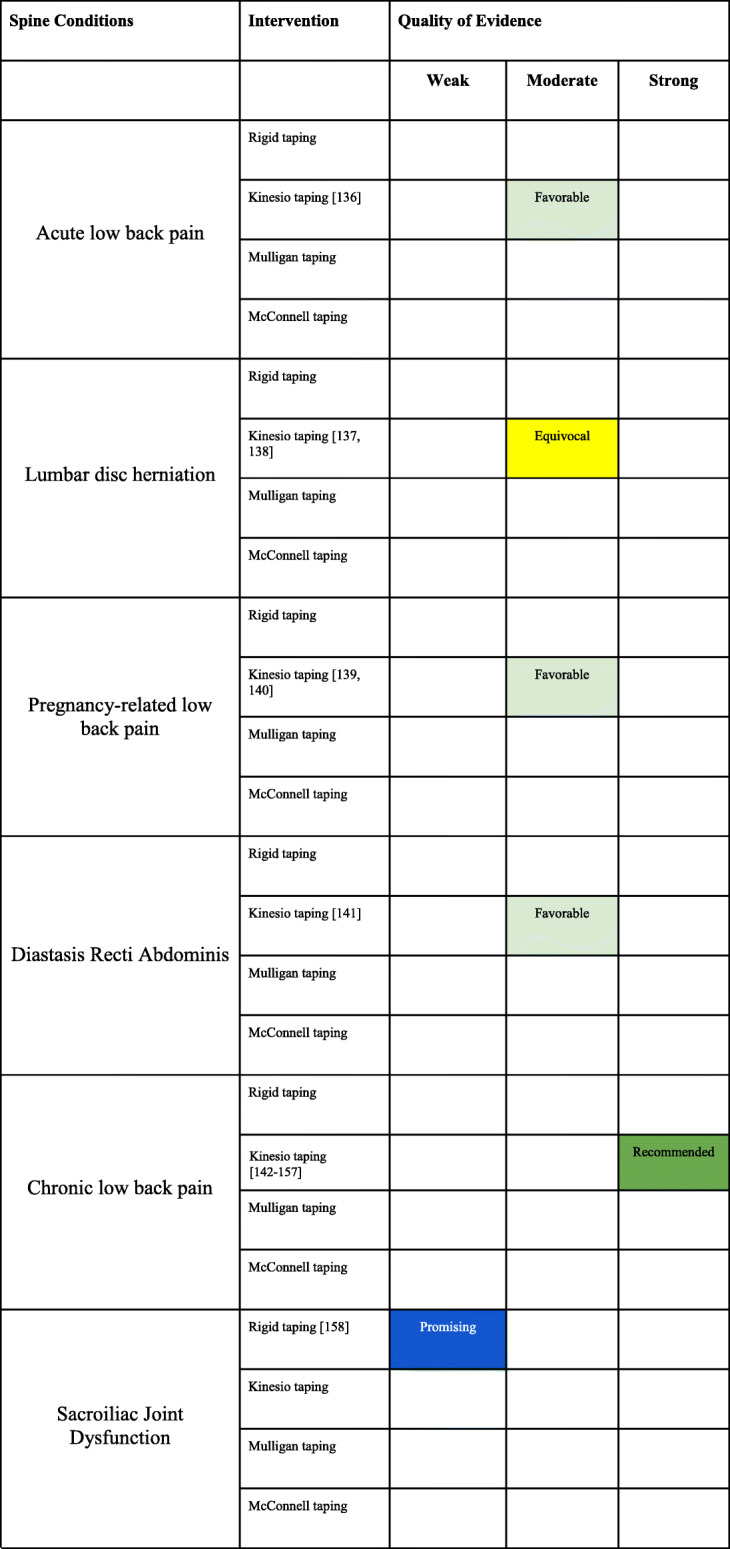

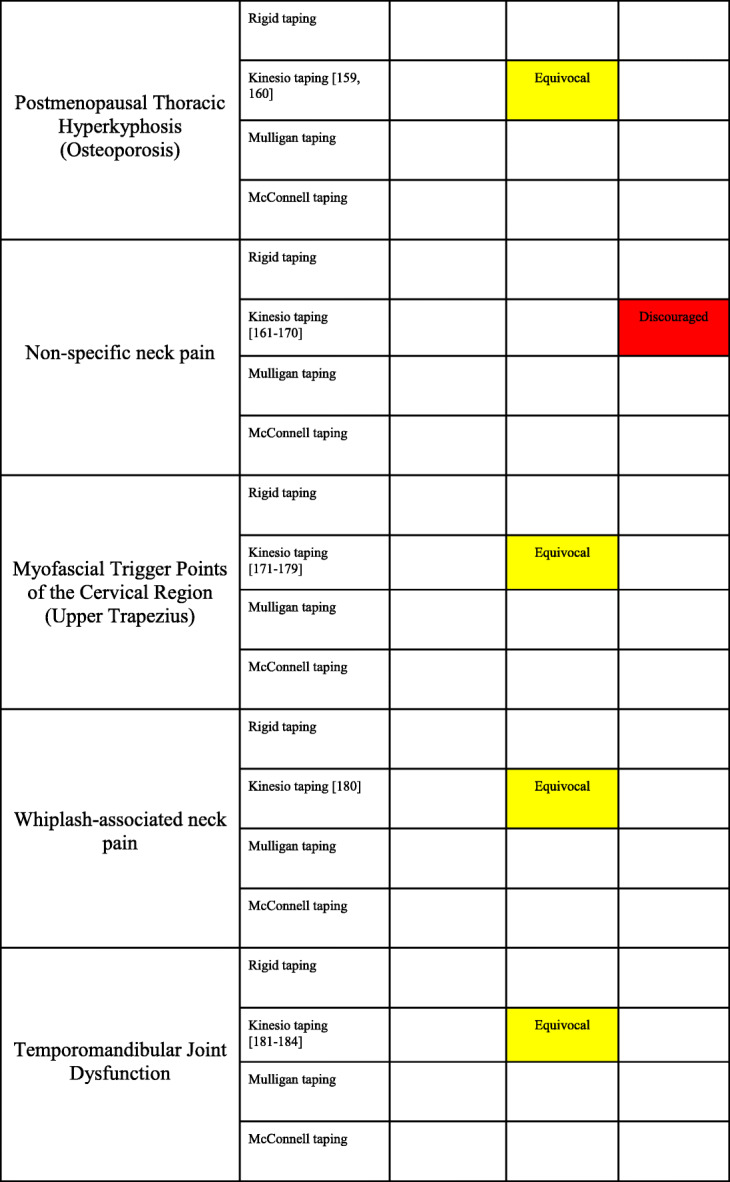
Spinal conditions interpretation of the evidence

##### Lumbar disc herniation

Evidence summary (Table 6)

- There is moderate evidence that the inclusion of KT in the treatment plan of lumbar disc herniation is equivocal.

##### Pregnancy-related low Back pain

Evidence summary (Table 6)

- There is moderate evidence that KT is beneficial for improving pain and disability for the treatment of pregnancy-related low back pain.

##### Diastasis recti abdominis

Evidence summary (Table 6)

- There is moderate evidence that KT is beneficial for improving pain and function for the treatment of diastasis recti abdominis.

##### Chronic low Back pain

Evidence summary (Table 6)

- There is strong evidence that KT improves pain and disability in patients with chronic non-specific low back pain.

##### Sacroiliac joint dysfunction

Evidence summary (Table 6)

- There is weak quality evidence that rigid tape is superior to no treatment for pain and function for the treatment of sacroiliac joint dysfunction.

#### Thoracic spine

There were no SRs related to the thoracic spine identified. There were two RCTs that evaluated KT for the treatment of pain and function secondary to osteoporotic thoracic kyphosis [159, 160] (Appendix 2 File: Appendix 2H) with PEDro scores ranging from 6 to 7. There were no RCTs that studied rigid taping, Mulligan taping or Mc-T in relation to conditions of the thoracic spine.

##### Evidence summary (Table 6)

- There is moderate evidence that KT alone or as part of multimodal rehabilitation is equivocal in the treatment of pain and kyphotic angle in cases of postmenopausal osteoporosis.

#### Cervical spine

There was one SR [135] that included conditions related to the cervical spine with an AMSTAR rating of 8 (Appendix 1 File: Appendix 1G). There were twenty RCTs related to the cervical spine that assessed KT, with overall PEDro scores ranging from 4 to 8 (Appendix 2 File: Appendix 2I). Ten RCTs pertained to mechanical or non-specific neck pain [161–170], nine RCTs pertained to trigger points or pain of the upper trapezius [171–179], and one RCT investigated whiplash associated neck pain [180]. There were no RCTs that studied rigid taping, Mulligan taping or Mc-T in relation to conditions of the cervical spine.

##### Mechanical neck pain

Evidence summary (Table 6)

- There is strong evidence that KT for mechanical neck pain is discouraged.

##### Upper trapezius pain (myofascial trigger points)

Evidence summary (Table 6)

- There is moderate evidence that the inclusion of KT in the treatment plan of upper trapezius pain is equivocal.

##### Whiplash associated neck pain

Evidence summary (Table 6)

- There is moderate evidence that the inclusion of KT in the treatment plan of whiplash associated neck pain is equivocal.

#### Temporomandibular joint

There were no SRs related to the temporomandibular joint identified. There were four RCTs related to the treatment of temporomandibular joint dysfunction with KT [181–184] (Appendix 2 File: Appendix 2J). The overall PEDro scores ranged from 4 to 5. There were no RCTs that studied rigid taping, Mulligan taping or Mc-T in relation to conditions of the temporomandibular joint.

##### Temporomandibular joint dysfunction

Evidence summary (Table 6)

- There is moderate evidence that KT is not superior in the treatment of pain and disability compared to occlusal splint, ischemic compression or exercise in people with temporomandibular joint dysfunction.

### Conditions not elsewhere classified

#### Myofascial pain syndrome

There was one SR assessing KT for the management of myofascial pain syndrome (Appendix 1 File: Appendix 1H) with an AMSTAR score of 3 [185]. There were two RCTs that investigated taping in the management of myofascial pain syndrome [186, 187] with PEDro scores that ranged from 3 to 5 (Appendix 2 File: Appendix 2K).

#### Myofascial pain syndrome

Evidence summary (Table 7)

- There is weak evidence that KT is not beneficial for pain and function in patients with myofascial pain syndrome.

**Table 7:**
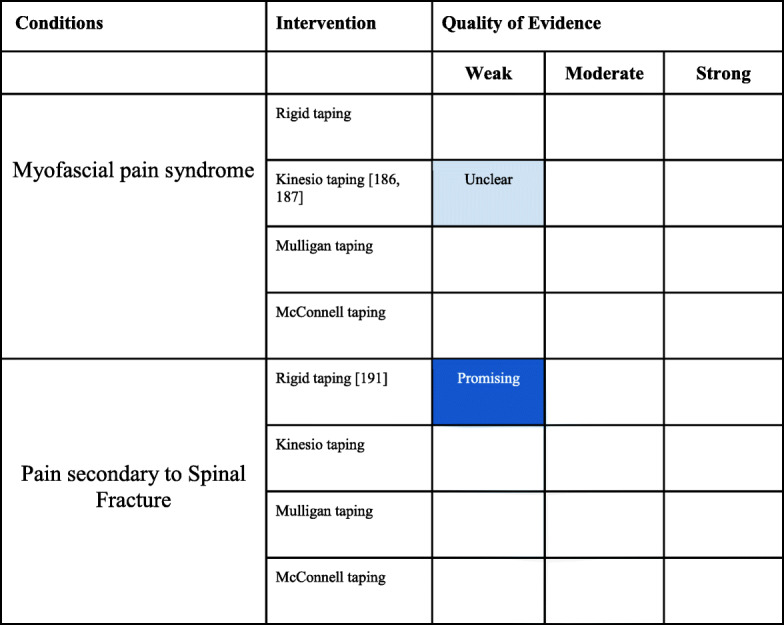
Not Otherwise Classified conditions interpretation of the evidence

### Other musculoskeletal conditions

There were three systematic reviews that could not be classified otherwise, yet met our inclusion criteria (Appendix 1I). One SR, with an AMSTAR of 7, evaluated elastic bandages for orthopedic and sports injuries [189]. Another SR, with an AMSTAR of 9, evaluated KT for chronic musculoskeletal pain [190]. One SR, with an AMSTAR 9 assessed the effects of KT for musculoskeletal conditions following intervention after a week [191]. There was one RCT that investigated rigid taping for the management of pain secondary to an active osteoporotic vertebral compression fracture of the thoracic or lumbar spine [188] with a PEDro score of 6 (Appendix 2 File: Appendix 2K).

Pain Secondary to Osteoporotic Vertebral Compression Fracture

#### Evidence summary (Table 7)

- There is weak evidence that rigid taping may be beneficial for pain and function in people with active osteoporotic compression fractures.

## Discussion

An evidence map is a tool to identify research gaps and clarify future research agendas, while presenting a user-friendly assessment of the evidence for clinicians as well as researchers [24]. As opposed to systematic reviews, evidence maps do not require a narrow question in a PICO format (population, intervention, comparison and outcome). While using a PICO format can be advantageous, it limited previous SRs to specific conditions in certain body regions or to a particular brand of tape. However, the use of an evidence map provides greater flexibility to review the entire body of taping literature. The unique contribution of this evidence map is that it provides a single summary source for all types of taping for all body regions related to a variety of different musculoskeletal conditions. Additionally, this evidence map helps to identify research gaps, and clarify conditions with research surplus. The final unique contribution of this evidence map is the concise clinician-friendly guide or “map” to the reviewed evidence related to taping of musculoskeletal conditions (Tables 4, 5, 6 and 7).

The results of this evidence map show that taping for musculoskeletal conditions is extensively used in clinical and research settings. With respect to SRs, we identified a wide heterogeneity in the populations studied, inclusion and exclusion criteria, taping methods, and outcome measures, resulting in a wide range of conclusions. This heterogeneity did not allow us to pool data or conduct a meta-analysis, making it difficult to provide broad generalizations about the effectiveness of taping on musculoskeletal conditions, based upon the information provided by these SRs.

With respect to the RCTs that we reviewed, the methodological rigor generally ranged from moderate to strong. The RCTs most commonly involved patients with spinal conditions, and KT was the type of tape most commonly studied (90/127 of RCTs), while the least common was Mulligan taping (2/127 of RCTs). Many RCTs used designs that included mixtures of treatment methods such as taping in combination with manual therapy or exercise. These multimodal interventions made it very difficult to parse out the main treatment effect due to taping alone, compared to global treatment effect that came from the combination of other therapeutic methods. However, it is promising that a majority of studies incorporated validate region-specific outcome assessments.

Several studies compared ‘placebo’ taping with ‘real’ taping, which was intended to control for the effects of contact with the clinician, but the number of studies that used this placebo-matched design was limited and had inherent methodological shortcomings. Sham taping and soft tissue therapies, as controls, are possibly inadequate options. Since most of the theorized benefits from taping is neurobiological, both controls will stimulate the same proprioceptive receptors that taping intervention methods are proposed to influence [10, 12, 21, 33]. Taping techniques have also been suggested to exert a mechanical effect on joint alignment and tracking, however, studies have not supported this therapeutic mechanism [31]. Theoretical mechanistic underpinnings for elastic and non-elastic taping strategies for musculoskeletal conditions are still under investigation [7, 192]. There is a similar conundrum in the acupuncture literature, questioning if sham or placebo acupuncture are in fact physiologically inert controls [193, 194]. A more adequate taping control would be methods that do not involve direct stimulation of these receptors, however, such methods are still under development [152].

It is important for clinicians to recognize the large variation in the scientific quality of taping research. This should be a warning to exercise caution when translating these results into clinical practice. Whenever a study of a taping method was found to provide no superior results to another rehabilitation intervention, we were keen on informing field clinicians that using the tape is equivocal. It should be noted that of the twenty five conditions evaluated, the effectiveness of KT was equivocal in most cases and one condition had a strong preponderance of evidence discouraging the use of KT for care. We suggest the use of KT be considered through shared decision making based on clinician experience and patient preference.

### Limitations

One limitation of this evidence map is the absence of a meta-analysis, which was not feasible due to the large heterogeneity in the patient populations (e.g. age groups, chronicity, athletic, geriatric, etc.), interventions (e.g. taping alone vs. multi-modal intervention), and research designs (e.g. incorporate blinding procedures, address placebo and sham concerns) of the included studies. Another limitation is that our results were derived only from studies that were published in English. However, only 4 (4.5%) of the SRs were excluded due to language from our search results which was Dutch, Persian, and Spanish.

## Recommendations for future research

Future research should consider the exploration of taping methods for musculoskeletal conditions related to the hip, elbow, wrist, hand and temporomandibular joints as there is a dearth of taping literature associated with these areas of the body. Also, future research should consider the inclusion of an adequate control or a placebo group, in order to delineate the clinical effect of taping from natural history, regression to the mean, or treatment expectation. Additionally, future research should consider isolating the clinical effectiveness of taping alone, separate and distinct from the many other co-interventions that are typically combined with taping (e.g. taping alone vs. rehab vs. taping plus rehab, or taping alone vs. joint manipulation vs. joint manipulation plus taping). Moreover, future research should not focus only on the immediate post-treatment effects of taping but also capture effectiveness data from outcomes derived from longer post-treatment follow-up periods. Finally, future research should consider comparative effectiveness designs, in which different types of taping methods (e.g. rigid taping versus KT) are compared with one another, as this would provide more clinically relevant information about the choice of taping strategies and may potentially reveal subgroup characteristics for treatment classification [195–197].

## Conclusion

The goal of this evidence map was to summarize and organize the current evidence on taping for musculoskeletal conditions for all regions of the body. Our results provide a clinician-friendly tool to assist with interpretation of the current state of the evidence regarding the effectiveness of taping for musculoskeletal conditions.

## Supplementary information

**Additional file 1: Appendix 1.** Systematic review extraction tables organized by body region.

**Additional file 2: Appendix 2.** Randomized controlled trail extraction tables organized by body region.

## Data Availability

All data generated or analysed during this study are included in this published article and its supplementary information files.

## Abbreviations

AMSTAR: A MeaSurement Tool to Assess systematic Reviews
CINAHL: Cumulative Index to Nursing and Allied Health Literature
KT: Kinesio tape
Mc-T: McConnell Taping
OA: Osteoarthritis
PFPS: Patellofemoral Pain Syndrome
PEDro: Physiotherapy Evidence Database
RCT: Randomized controlled trial
SIS: Subacromial Impingement Syndrome
SR: Systematic review
