# Supplementary material for: Taping for conditions of the musculoskeletal system: an evidence map review
Source: Chiropr Man Therap. 2020 Sep 15;28:52. doi: 10.1186/s12998-020-00337-2 (PMC7491123; doi:10.1186/s12998-020-00337-2)
Supplement: Supplementary file 1 — Additional file 1: Appendix 1. Systematic review extraction tables organized by body region. [file 12998_2020_337_MOESM1_ESM.docx]

**APPENDIX 1: SYSTEMATIC REVIEWS OF TAPING**

**APPENDIX 1A: EVIDENCE TABLE FOR SYSTEMATIC REVIEWS OF THE KNEE**

| Review title (Primary Author/year) | Population | Search Dates | # of included studies | Intervention(s) | Summary of Results | AMSTAR score |
| --- | --- | --- | --- | --- | --- | --- |
| Patellar taping for patellofemoral pain: a systematic review and meta-analysis to evaluate clinical outcomes and biomechanical outcomes  (Barton / 2014) [27] | Adults with patellofemoral pain syndrome including anterior knee pain, chondromalacia  patellae | 1/1/13 | 20 | Patellar taping | Moderate evidence to include patellar taping in management of patellofemoral pain syndrome will most likely have a large effect on reducing pain and improving functional capacity in the immediate term during accompanying rehabilitation exercises.  Patellar taping also appears to be an effective adjunct to exercise over a 4-week period. | 8/11  73% |
| An update for the conservative management of patellofemoral pain syndrome: a systematic review of the literature from 2000 to 2010  (Bolgla / 2011) [28] | Individuals with patellofemoral pain syndrome or anterior knee pain | 1/1/2000 to 12/31/2010 | 22 | Hip strengthening  Quadriceps strengthening  Patella taping  Patella bracing/knee bracing  Foot orthosis | There is support for the use of taping in conjunction with exercise at least for the short-term. Mechanism that taping uses is still unknown. Manner of tape correction may not necessarily influence its beneficial effects. Taping over exercise alone was not supported. Also, taping for long-term symptoms associated with patellofemoral pain syndrome is minimal. | 4/11  36% |
| Patellar taping for patellofemoral pain syndrome in adults  (Callaghan / 2012) [29] | Adults with patellofemoral pain syndrome | 8/1/11 | 5 | Patellar taping | Current available evidence with clinically relevant outcomes is low quality and insufficient to draw conclusions on effects of taping, either alone or as part of a program. | 10/11  91% |
| Effects of Kinesio Taping versus McConnell Taping for Patellofemoral Pain Syndrome: A Systematic Review and Meta-Analysis  (Chang / 2015) [18] | Individuals with patellofemoral pain syndrome | 7/31/2014 | 11 | KT  McConnell taping  Athletic tape (Endura-Fix tape, Protape, Leukotape P rigid tape, Hypafix tape)  Patellar taping with neuromuscular retraining | The use of KT for patellofemoral pain syndrome patients had a small effect in pain relief.  There were small increases in motor function improvement and moderate increases in muscle activity among patellofemoral pain syndrome patients who used KT.  McConnell and KT significantly improve muscle activity, motor function, and quality of life, benefits, which are possibly facilitated by pain relief. | 10/11  91% |
| Efficacy of Nonsurgical Interventions for Anterior Knee Pain: Systematic Review and Meta-Analysis of Randomized Trials  (Collins / 2012) [30] | Individuals with anterior or retropatellar knee pain | 11/30/09 | 27 | Foot orthoses with and without physical therapy  Exercise  Closed chain exercises Patella taping in conjunction with exercise  Acupuncture | Short-term data showed significant large to very large effects of 4 weeks of taping and exercise over exercise alone, and over placebo tape with exercise. Longer-term data shows no significant between-group effects when patellar taping and education were compared to education alone, and when patellar taping was added to exercise and education. | 7/11  64% |
| The use of McConnell taping to correct abnormal biomechanics and muscle activation patterns in subjects with anterior knee pain: a systematic review  (Leibbrandt / 2015) [31] | Individuals <40 years old diagnosed with anterior knee pain | 6/30/2014 | 8 | McConnell taping Placebo  No taping | McConnell taping does not alter knee kinematics and kinetics or muscle activation patterns of the knee muscles.  There is currently inadequate evidence for the effect of McConnell taping on biomechanics and muscle activation in individuals with anterior knee pain. | 10/11  91% |
| Effects of Elastic Taping on knee osteoarthritis: A systematic review and meta-analysis  (Li, X / 2018) [34] | Individuals with knee OA | 09/26/2018 | 11 | Elastic Taping  No treatment  Placebo KT  Sham Taping | There is underpowered evidence to suggest that elastic taping is  effective in the treatment of knee OA.  The interpretation of our results should be considered cautiously due to the limitations of the included trials, such as methodological drawbacks and poor data quality. | 9/11  81% |
| Systematic Review of the Effect of Taping Techniques on Patellofemoral Pain Syndrome  (Logan / 2017) [32] | All ages with anterior knee pain patellofemoral pain symptoms | 1/1/1995 to 4/30/2015 | 5 | Tension taping and exercise  Placebo taping  Taping alone | Taping alone does not significantly reduce pain.  Knee taping, including placebo taping, combined with exercise provides superior reduction in pain compared with exercise alone.  Rehabilitation programs should be multifactorial, with an emphasis on exercise therapy and education, while utilizing adjuncts, such as knee taping, to complement the treatment regimen. | 8/11  73% |
| Non-elastic taping, but not elastic taping, provides benefits for patients with knee osteoarthritis: systemic review and meta-analysis  (Ouyang / 2017) [33] | Adults with knee OA | 5/31/2017 | 11 | Leukotaping  Kinesiotaping  Compared to control taping | The leukotaping effects are more likely to be temporary rather than curative.  The results do not support the benefit effects of KT. However, these elastic taping studies could evaluate mixed temporary and treatment effects, which limited us to understand the true value of KT.  The current evidence suggests the use of leukotaping to temporarily control the symptoms of knee OA. | 8/11  73% |

**Appendix 1B: EVIDENCE TABLE FOR SYSTEMATIC REVIEWS OF THE ANKLE**

| Review title (Primary Author/year) | Population | Search Dates | # of included studies | Intervention(s) | Summary of Results | AMSTAR score |
| --- | --- | --- | --- | --- | --- | --- |
| A systematic review on the effectiveness of external ankle supports in the prevention of inversion ankle sprains among elite and recreational players  (Dizon / 2009) [7] | Adolescent and adult athletes with previous injuries | 2009 | 6 (2 taping studies) | Tape  Brace  Orthosis | Good evidence for either ankle taping or ankle braces to prevent lateral ankle sprains among previously injured players. Without previous ankle injuries, effects still need to be proven. No evidence on which external ankle support is better than the other. | 8.5/11  77% |
| A systematic review on the treatment of acute ankle sprain: Brace versus other functional treatment types  (Kemler / 2011) [76] | Sports and non-sports participants with an acute ankle injury | 1/1/1990 to 4/1/2009 | 8 | Braces  Tubigrip®,  Elastic wrap  Ankle tape | In terms of functional outcomes, ankle braces are more effective than other types of functional treatment for treating acute ankle sprains. Findings of other studies suggest that the use of ankle braces is more cost-effective and should be considered for the treatment of acute ankle sprains. | 9/11  82% |
| The effect of ankle taping or bracing on proprioception in functional ankle instability: A systematic review and meta-analysis  (Raymond / 2012) [77] | Young adults with recent or chronic sprained ankle or functional ankle instability | 3/1/12 | 8 | Ankle brace  Ankle taping | Ankle tape or brace has no effect on proprioception and may in fact make proprioception worse in the inversion/eversion plane where proprioception is measured as threshold to movement detection. Taping and bracing should not be discouraged because they may still prevent injury; it is unlikely that the protective effect is due to enhanced proprioception however. | 9/11  82% |
| Managing ankle sprains in primary care: what is best practice? A systematic review of the last 10 years of evidence  (Seah / 2011) [78] | Adults with acute or chronic ankle sprain | 1/1/2000 to 12/31/2009 | 33 | Elastic bandaging  Soft casting  Taping  Orthoses with coordination training  Immobilization in a below-knee cast or pneumatic brace  Tubular compression bandage.  Lace-up brace  Semi-rigid orthoses and Supervised rehabilitation training | Two studies in this systematic review referred to taping. One found that function interventions, including taping, is better than immobilization of multiple outcome measures. The other found elastic bandaging to be less of an effective functional treatment than lace-up supports. | 2.5/11  23% |
| Kinesio taping is superior to other taping methods in ankle functional performance improvement: a systematic review and meta-analysis  (Wang / 2018) [79] | Healthy Adults and Adults with ankle sprains | 03/31/2018 | 10 | KT  No tape  Placebo tape | KT improves dynamic balance in participants with or without ankle instability compared with other taping methods.  KT ensures a preferable vertical jump height while not reducing ankle mobility compared with athletic taping or elastic taping. | 7/11  63% |
| The effects of Kinesiotape applied to the lateral aspect of the ankle: relevance to ankle sprains -- a systematic review  (Wilson / 2015) [80] | Athletes and non-athletes lateral ligament ankle sprains | 4/12/2014 | 8 | KT  Placebo/sham tape  No tape | It is recommended that KT could be used in clinical practice to prevent lateral ankle injuries through its effects on postural control, and manage lateral ankle injuries, due to its positive effects on proprioception, muscle endurance and activity performance. It must be noted that KT may not provide sufficient mechanical support to unstable ankles to facilitate improved confidence during the performance of postural control tasks. Adverse events associated with KT appear unlikely. | 8/11  73% |
| The Effectiveness of Physical Agents for Lower-Limb Soft Tissue Injuries: A Systematic Review  (Yu / 2016) [81] | Adults and children with grade I/II sprains and  strains, tendinopathy, and nonspecific  lower-limb pain, excluding major pathology | 1/1/1990 to 1/26/2015 | 23 | Low-level laser therapy  Cryotherapy  Extracorpeal shockwave therapy  Orthoses  Low-dye taping  Taping  Ultrasound  Brace  Electrical muscle stimulation  Night splints | For recent grade II/III ankle sprains, the choice of assistive devices should be based on shared decision-making between clinicians and patients, as various assistive devices (ie, tape, compression bandage, semi-rigid brace or boot, and immobilization walking cast) lead to similar outcomes.  The true effectiveness of cryotherapy or assistive devices (ie, tape, compression bandage, semi-rigid brace or boot, and immobilization walking cast) is not revealed in these studies because they included no sham/placebo group. | 8/11  73% |

**APPENDIX 1C: EVIDENCE TABLE FOR SYSTEMATIC REVIEWS OF THE FOOT**

| Review title (Primary Author/year) | Population | Search Dates | # of included studies | Intervention(s) | Summary of Results | AMSTAR score |
| --- | --- | --- | --- | --- | --- | --- |
| Efficacies of different external controls for excessive foot pronation: a meta-analysis  (Cheung / 2011) [85] | Healthy adults diagnosed with musculoskeletal conditions likely to be related to excessive foot pronation | 11/1/10 | 29 | Foot orthoses  Motion control footwear  Therapeutic adhesive tape | Taping was found to be more effective at reducing calcaneal eversion than both footwear and orthoses. Part of this could be due to the constant readjustment with reapplication of tape. Low-dye taping, although one of the most popular taping methods, was not found to be effective in checking excessive foot pronation. | 8.5/11  77% |
| A physiological and psychological basis for anti-pronation taping from a critical review of the literature  (Franettovich / 2008) [86] | Individuals with foot pronation | 6/1/06 | 22 | Low-dye taping | Anti-pronation tape was found to change foot and leg posture both statically and possibly dynamically. Preliminary evidence suggests that anti-pronation tape alters muscle activity in the leg during dynamic activity, but caution is advised in interpreting results of a few studies of small sample sizes. The placebo effect of taping is not well understood, but there appears evidence that this idea should not be discounted in anti-pronation taping. | 5/11  45% |
| Taping for plantar fasciitis  (Podolsky / 2015) [82] | Patients with plantar fasciitis | 12/1/12 | 8 | Low-dye taping  Calcaneal taping | In the short-term, taping is beneficial and can be implemented as an immediate pain reliever. The recommended taping techniques are low-dye taping and calcaneal taping. | 6/11  55% |
| The effect of low-dye taping on kinematic, kinetic, and electromyographic variables: a systematic review  (Radford / 2006) [87] | Athletes and non-athletes with and without foot pathology | 11/15/2005 | 5 | Low-dye taping  No taping | Some kinematic changes to the foot occur after taping application (particularly navicular height after application), suggesting a reduction in foot pronation. The result is not known to be of clinical significance, however, as these trials were scientific in nature. Further studies should focus on patient centered outcomes, such as pain, function, and quality of life. | 7/11  64% |
| The effectiveness of conservative, non-pharmacological treatment, of plantar heel pain: A systematic review with meta-analysis  (Salvioli / 2017) [83] | Adults with a diagnosis of plantar heel pain or plantar fasciitis | 3/10/2017 | 20 (4 meta-analyses) | Extracorpeal shock wave therapy  Laser  Orthoses  Stretching  Ultrasound guided pulsed radiofrequency  Dry needling  Low-dye taping  Calcaneal Taping  Placebo  No treatment  Sham treatment | Shock wave therapy, laser therapy, orthoses, Ultrasound Guided Pulsed Radiofrequency, dry-needling and calcaneal taping all showed some beneficial effect in reducing plantar heel pain, in comparison to placebo, although some interventions did not achieve a significant reduction, and evidence quality was only low or moderate for most interventions. Thus, no firm, evidence-based clinical advice can be given on the relative reduction of plantar heel pain. | 10/11  91% |
| Efficacy of taping for the treatment of plantar fasciosis: a systematic review of controlled trials  (van de Water / 2010) [84] | Patients with plantar fasciosis not related to trauma and provoked with physical activity | 10/7/07 | 5 | No treatment  Orthotic  Medication  Ultrasound  Injections  Taping | There is limited, but supporting evidence of a positive effect of taping as an intervention or part of an intervention for patients with plantar fasciosis on pain in the short term. Inconclusive results were found concerning disability improvement. | 10/11  91% |

**APPENDIX 1D: EVIDENCE TABLE FOR SYSTEMATIC REVIEWS OF THE SHOULDER**

| Review title (Primary Author/year) | Population | Search Dates | # of included studies | Intervention(s) | Summary of Results | AMSTAR score |
| --- | --- | --- | --- | --- | --- | --- |
| The efficacy of taping for rotator cuff tendinopathy: a systematic review  (Desjardins-Charbonneau / 2015) [88] | 18 years or older with rotator cuff tendinopathy, partial rotator cuff tear, impingement syndrome or subacromial bursitis | 10/1/2014 | 10 | Non-elastic tapping  KT | Because of the high risk of bias and the heterogeneity of results of the included studies, the authors cannot, at this time, formally recommend taping for treatment of rotator cuff tendinopathy.  There is insufficient evidence to formally conclude on the efficacy of non-elastic taping used alone or in conjunction with other interventions to treat rotator cuff tendinopathy.  KT might improve pain free ROM but those improvements do not translate into pain reduction and increased function. There is insufficient evidence to recommend KT in conjunction with another intervention. | 9/11  82% |
| Treatments for shoulder impingement syndrome: a PRISMA systematic review and network meta-analysis  (Dong / 2015)[89] | Adults diagnosed with subacromial impingement syndrome not caused by other systemic disease or trauma | 4/15/2014 | 52 (qualitative synthesis)  33 (quantitative synthesis) | Acupuncture therapy  Corticosteroid injection  Diacutaneous fibrolysis therapy  Exercise treatment  KT  Low-level laser therapy  Nonsteroidal anti-inflammatory drug injection  Pulsed electromagnetic field therapy  Specific exercise therapy  Ultrasound therapy  Arthroscopic subacromial decompression  Arthroscopic bursectomy without acromioplasty  Open Subacromial decompression  Platelet-leuokocyte gel injection  Radiofrequency therapy | For those patients who seek nonoperative treatment option at an early stage of subacromial impingement syndrome, exercise combined with other therapies should be recommended. Among these therapies, KT, specific exercises, and acupuncture therapy should be considered as the first line choices, whereas pulsed electromagnetic field therapy, localized corticosteroid injection, diacutaneous fibrolysis, and ultrasound therapy may be considered as the second-line treatment choices; however, low-level laser therapy and the localized injection of nonsteroidal antiinflammatory drugs are not recommended. | 6/11  54% |
| Effectiveness of physical therapy treatment of clearly defined subacromial pain: a systematic review of randomized controlled trials  (Haik / 2016) [90] | Adults with subacromial pain syndrome | 4/2015 | 64 | All types of active or passive physical therapy interventions with high methodological quality RCT were included  Pulsed electromagnetic field  Ultrasound  Microwave therapy  Transcutaneous electrical nerve stimulation  Exercise therapy  Manual therapy  Taping  Acupuncture | There is moderate evidence that KT does not produce additional benefits over mixed therapy protocols or placebo taping to reduce pain, improve function or increase the ROM. | 6/11  54% |
| Effectiveness of stretching exercise versus kinesiotaping in improving length of pectoralis minor: a systematic review and network meta-analysis.  (Lai / 2019) [91] | Healthy adults without symptomatic shoulders and Adults with shoulder pain or with rounded shoulders | 1/2019 | 6 | KT  PNF stretch  Static stretch | Compared with no intervention, KT can be beneficial for lengthening the pectoralis minor.  Intervention with static stretching alone has no effect on pectoralis minor length.  Compared with KT and no intervention, proprioceptive neuromuscular facilitation stretching can increase pectoralis minor index. | 7/11  63% |
| Clinical outcomes of a scapular-focused treatment in patients with subacromial pain syndrome: a systematic review  (Reijneveld / 2017) [92] | Patients with Subacromial impingement syndrome | 2/23/2015 | 4 | Scapular focused exercise  Scapular focused exercise and scapular mobilization techniques  Scapular taping | No evidence was found to support the use of scapular taping. | 8/11  72% |
| Does taping in addition to physiotherapy improve the outcomes in Subacromial impingement syndrome? A systematic review  (Saracoglu / 2017) [93] | Adults with shoulder impingement | 2008 to 6/2015 | 4 | KT  Rigid taping  Sham Taping  Usual physiotherapy care | Taping, in addition to physiotherapy interventions (e.g. exercise, electrotherapy, and manual therapy), might be an optional modality for managing patients with shoulder impingement syndrome, especially for the initial stage of the treatment.  Further robust, placebo-controlled and consistent studies are needed in order to prove whether it is more effective than physiotherapy interventions without taping. | 10/11  91% |
| Effectiveness of conservative interventions including exercise, manual therapy and medical management in adults with shoulder impingement: a systematic review and meta-analysis of RCTs  (Steuri / 2017) [94] | Adults with shoulder impingement | 1/2017 | 77 (quantitative synthesis)  23  (qualitative synthesis) | Corticosteroid injections  Nonsteroidal anti-inflammatory drugs  Exercise  Manual therapy  Ultrasound  Laser  Extracorporeal shockwave therapy  Tape  Nerve block | Exercise, especially shoulder-specific exercises, should be prescribed for all patients with shoulder impingement. The addition of manual therapy, tape, extracorpeal shockwave therapy and laser might add a small benefit. | 8/11  72% |
| Effectiveness of passive physical modalities for shoulder pain: systematic review by the Ontario protocol for traffic injury management collaboration (Yu / 2014) [95] | Adults and children with soft tissue injuries of the shoulder | 1/1/1990 to 4/18/2013 | 11 | Extracorporeal shockwave therapy  Ultrasound  Low level laser therapy  Bipolar interferential current therapy  Local microwave diathermy  Tape | Pretensioned tape and shock-wave therapy are not more effective than placebo therapy for subacromial impingement syndrome. | 7/11  63% |

**APPENDIX 1E: EVIDENCE TABLE FOR SYSTEMATIC REVIEWS OF THE ELBOW**

| Review title (Primary Author/year) | Population | Search Dates | # of included studies | Intervention(s) | Summary of Results | AMSTAR score |
| --- | --- | --- | --- | --- | --- | --- |
| Sticking to the facts: A systematic review of the effects of therapeutic tape in lateral epicondylalgia  (George / 2019) [129] | Individuals with lateral epicondylalgia | 3/2018 | 8 | KT  Rigid Taping  Placebo tape  No tape | There is likely immediate improvement in pain and function in individuals with lateral epicondylalgia treated with rigid tape.  The evidence is unclear whether kinesiotape influences pain and function immediately or in the short-term. | 9/11  82% |

**APPENDIX 1F: EVIDENCE TABLE FOR SYSTEMATIC REVIEWS OF THE WRIST AND HAND**

| Review title (Primary Author/year) | Population | Search Dates | # of included studies | Intervention(s) | Summary of Results | AMSTAR score |
| --- | --- | --- | --- | --- | --- | --- |
| The effectiveness of passive physical modalities for the management of soft tissue injuries and neuropathies of the wrist and hand: a systematic review by the Ontario Protocol for Traffic Injury Management (OPTIMa) collaboration  (D’Angelo / 2015) [130] | Adults diagnosed with soft tissue injuries and neuropathies of the wrist and hand | 1/1/1990 to 1/19/2015 | 11 | Low-level laser therapy  Orthoses  Surgery  Acupuncture  KT  Multimodal physical therapy  Thumb spica cast  Corticosteroid injection | Found evidence that KT and a thumb spica cast may provide short-term benefits to patients with de Quervain disease. | 8/11  72% |

**APPENDIX 1G: EVIDENCE TABLE FOR SYSTEMATIC REVIEWS OF THE SPINE**

| Review title  (Primary Author/year) | Population | Search Dates | # of included studies | Intervention(s) | Summary of Results | AMSTAR score |
| --- | --- | --- | --- | --- | --- | --- |
| Effects of kinesiotape on pain and disability in individuals with chronic low back pain: a systematic review and meta-analysis of randomized controlled trials.  (Li, Y / 2019) [131] | Individuals with chronic low back pain | 02/13/2018 | 19 | KT  Sham  Placebo taping | Individuals with chronic low back pain experience statistically significant improvements in disability through the sole application of KT.  For patients with chronic low back pain, the addition of KT to physical therapy/exercise does not lead to extra effect on pain reduction or disability improvement. . | 5/11  45% |
| Effectiveness of kinesio taping in patients with chronic nonspecific low back pain: a systematic review with meta-analysis  (Luz Junior / 2019) [132] | Individuals with chronic non-specific low back pain | 2/26/2018 | 11 | KT  Placebo  No taping  KT with Exercise | There is low to very low quality evidence that KT was not superior to no intervention, placebo, other intervention or KT combined with exercise at any time of the follow-ups.  Studies targeting to evaluate the effects of KT at both intermediate and long term should be conducted.  The duration of use of KT should also be further investigated as there is no consensus on optimal dosage of this intervention. | 8/11  72% |
| Kinesio taping for chronic low back pain: A systematic review.  (Nelson / 2016) [133] | Individuals with chronic low back pain | Inception to 6/17/2015 | 5 | KT  physical therapy (stretching of back, iliopsoas and hamstrings; strengthening for abdominal muscles) | The effect of KT on the assessed parameters is small and may be most beneficial as an adjunctive therapy for individuals with chronic low back pain.  These results suggest that the precise method of taping may not be as critical to improving outcomes as originally thought. | 4/11  36% |
| Kinesio taping in treatment of chronic non-specific low back pain: a systematic review and meta-analysis  (Sheng / 2019) [134] | All ages with chronic non-specific low back pain | 7/31/2018 | 8 | KT  Physical therapy  Acupuncture  Sham taping  No taping  Modalities | KT, in isolation or as part of a package of care, is superior to no taping, acupuncture and other general physical therapy modalities to reduce pain and disability, for chronic non-specific low back pain patients. | 7/11  63% |
| Effect of taping on spinal pain and disability: systematic review and meta-analysis of randomized trials  (Vanti / 2015) [135] | Symptomatic adults with acute, subacute, or chronic specific or  non-specific spinal pain, myofascial pain syndrome, or whiplash associated disorder | Inception to 6/2014 | 8  (5 low back pain, 3 neck pain) | Elastic or non-elastic taping  Multimodal treatment without any explicit investigation on the distinct effects of each procedure were excluded | Concerning clinical relevance, no differences between experimental and control groups were found.  Only Chen and colleagues’ study on nonelastic taping yielded results in the experimental group that attained the threshold minimal clinically important difference for short-term pain and disability reduction, differently from placebo.  There is low-quality evidence that elastic taping versus sham/placebo or no treatment provides no significant improvement in disability in the immediate posttreatment period and at 1- and 2-month follow-ups.  There is low-quality evidence that non-elastic taping versus placebo provides no significant improvement in disability at posttreatment follow-up and at 1-month follow-up.  There is low-quality evidence from one trial that elastic taping versus placebo reduces pain in the immediate post-treatment period.  Very low-quality evidence (from one trial) that nonelastic taping versus no treatment provides no significant reduction in pain or disability in the immediate post-treatment period. | 8/11  72% |

**APPENDIX 1H: EVIDENCE TABLE FOR SYSTEMATIC REVIEWS OF THE MYOFASCIAL PAIN SYNDROME**

| Review title  (Primary Author/year) | Population | Search Dates | # of included studies | Intervention(s) | Summary of Results | AMSTAR score |
| --- | --- | --- | --- | --- | --- | --- |
| Evidence for kinesio taping in management of myofascial pain syndrome: a systematic review and meta-analysis  (Zhang / 2019) [185] | Individuals diagnosed with myofascial pain syndrome or myofascial trigger points | 11/2018 | 20 | KT  No tape  Placebo tape | Kinesio taping is statistically superior to other treatments for relieving the pain intensity and ROM of patients with myofascial pain syndrome at post-intervention.  The follow-up effectiveness of KT over other treatments still remains unclear.  Few studies measured disability, so the limited evidence did not find any reduction in the disability. | 3/11  27% |

**APPENDIX 1I: EVIDENCE TABLE FOR SYSTEMATIC REVIEWS MISCELLANEOUS MUSCULOSKELETAL CONDITIONS**

| Review title (Primary Author/year) | Population | Search Dates | # of included studies | Intervention(s) | Summary of Results | AMSTAR score |
| --- | --- | --- | --- | --- | --- | --- |
| Elastic Bandaging for Orthopedic and Sports Injury Prevention and Rehabilitation: A Systematic Review  (Fousekis / 2017) [189] | Athletes  (first aid, sports injuries, orthopedic injuries, and sports injuries prevention and rehabilitation) | Inception to 2/2015 | 12 | Elastic non-adhesive bandages | Evidence to date suggests that rehabilitation with the use of elastic non adhesive bandages may be inferior to braces in achieving more effective edema reduction and an earlier return to optimal functional status after ankle-joint injuries.  The current review found a small amount of evidence supporting the idea that elastic non-adhesive bandage improves knee and ankle proprioception in in patients with OA and meniscal problems and healthy individuals.  It seems that elastic non-adhesive bandage has a minimal protective function in terms of reduction of injury risk through improvement of joint stability.  Elastic non-adhesive bandage can lead to a significant reduction in both passive and active ROM.  The findings of this review are unable to fully support the use of elastic non-adhesive bandage against other taping techniques for musculoskeletal-injury prevention and rehabilitation. | 7/11  64% |
| Kinesio taping in musculoskeletal pain and disability that lasts for more than 4 weeks: is it time to peel off the tape and throw it out with the sweat? A systematic review with meta-analysis focused on pain and also methods of tape application  (Lim / 2015) [190] | Chronic musculoskeletal pain | Inception to 7/2/2014 | 17 | KT | KT proved to be superior to no taping, sham taping and usual care, for the reduction of pain in individuals with more than 4 weeks of musculoskeletal pain.  KT is not more effective than other forms of intervention in reducing pain. KT is also not more effective than minimal or other forms of intervention in the reduction of disability related to chronic musculoskeletal pain. | 9/11  82% |
| Effects of kinesio taping alone versus sham taping in individuals with musculoskeletal conditions after intervention for at least one week: a systematic review and meta-analysis  (Ramirez-Valez / 2019) [191] | Individuals >18 years of age with musculoskeletal conditions. | 01/15/2018 | 6 | KT  Sham taping | KT does not provide superior effects on pain and disability scores or trunk flexion ROM when compared with sham intervention.  There is inconclusive and low-quality evidence of a beneficial  effect of KT alone over sham taping in patients with low back pain. | 9/11  81% |

Abbreviations

Kinesio tape - KT

Osteoarthritis - OA

Range of motion - ROM
