# Supplementary material for: Taping for conditions of the musculoskeletal system: an evidence map review
Source: Chiropr Man Therap. 2020 Sep 15;28:52. doi: 10.1186/s12998-020-00337-2 (PMC7491123; doi:10.1186/s12998-020-00337-2)
Supplement: Supplementary file 2 — Additional file 2: Appendix 2. Randomized controlled trail extraction tables organized by body region. [file 12998_2020_337_MOESM2_ESM.docx]

**APPENDIX 2: RANDOMIZED CONTROLLED TRIALS OF TAPING**

**APPENDIX 2A: EVIDENCE TABLE FOR RANDOMIZED CONTROLLED TRIALS OF THE KNEE**

| Study | Population description | Sample size | Interventions | Outcomes/time points | Results | Key author conclusions | PEDro Score |
| --- | --- | --- | --- | --- | --- | --- | --- |
| Akbas  (2010) [35] | **Age**: 17-50  **Diagnosis**: Patellofemoral pain syndrome    **Inclusion**: unilateral, female referred by orthopedic consultant  **Exclusion**: tendonitis, Osgood-Schlatter syndrome, known articular cartilage, meniscus or ligament damage, history of patellar subluxation or dislocation and previous knee surgery | N = 31 | **T_1_**: Kinesio Tape (KT), strengthening, stretching  (n=15)  **T_2_**: strengthening, stretching  (n=16) | **Pain**:  VAS during 9 activities: resting, prolonged sitting with knees flexed, kneeling, walking, squatting, ascending and descending stairs, going up and down hill  **Disability**:  Anterior Knee Pain Scale/Kujala Scale  **Functional**: Obers test  Hamstring tension  **Time points**: 6 week intervention duration   1. Baseline 2. 3 week 3. Post-treatment | **Pain**:  Decreased significantly in all positions in both groups with no significant between groups difference  **Disability**:  Anterior Knee Pain Scale increased in both significantly, but no between group differences present  **Functional**:  Pain Hamstring tension significantly decreased in both groups - more gradually in the control group and in the first three weeks for the KT group. Iliotibial band/tensor fasica lata length increased significantly in both groups, control group in the last three weeks | Adding KT to a conventional exercise program does not improve the results in patients with patellofemoral pain syndrome, other than a faster improvement in hamstring muscle flexibility. | 5 |
| Aydoğdu (2017) [43] | **Age**: not specifically described  **Diagnosis**: knee osteoarthritis    **Inclusion**: unilateral knee osteoarthritis according to  the American College of Rheumatology, stage 2 and 3 based on the Kellgren-Lawrence criteria  **Exclusion**: previous knee joint operation, pregnancy or mental problems preventing them from doing exercise; participants with infection in the areas close to the knee joint, metal implant, or allergy to KT | N = 54 | **T_1_**: KT and conventional treatment  (n=28)  **C**: conventional treatment (ultrasound, TENs, exercise, cold)  (n=26) | **Pain**:  VAS  **Disability**:  Knee Injury Osteoarthritis Outcome Score (KOOS)  **Functional**: ROM  Muscle strength (dynamometer)  **Time points**: 3 week intervention duration   1. Baseline 2. 1 hour after initial taping (intervention group only) 3. Post-treatment | **Pain**:  There was a statistically significant improvement in VAS for both groups.  There was no significant difference between group  **Disability**:  There was a statistically significant improvement in KOOS for both groups.  There was no significant difference between group  **Functional**:  There was a statistically significant improvement in ROM, quadriceps muscle strength for both groups.  There were no significant differences with these parameters between group | KT has significant immediate effects after a single KT application on ROM, pain and functional status in patients with knee osteoarthritis.  KT in addition to classical treatment is not superior to classical treatment alone in terms of clinical outcomes over 3 weeks later. | 6 |
| Bennell  (2005) [44] | **Age**: > 50  **Diagnosis**: Knee osteoarthritis    **Inclusion**: knee pain on most  days of the past month (average pain severity of > 4 on an 11 point numerical rating scale), osteophytes on x ray (as assessed by an experienced radiologist), and pain or difficulty in rising from sitting or climbing stairs.  **Exclusion**: physiotherapy or knee surgery (in the previous 12 months), lower limb arthroplasty, Synvisc^®^ or intra-articular steroid injections (in the previous six months), a systemic arthritic condition, a severe medical condition, poor skin condition, known allergic reaction to tape, or a body mass index of more than 36 kg/m2 (which would result in  difficulty in knee taping). | N = 140 | **T_1_**: Exercise, massage, taping (rigid), mobilisation  (n=73)  **T_2_**: placebo treatment [sham ultrasound, no treatment]  (n=67) | **Pain**:  VAS  **Disability**:  Western Ontario and McMaster Universities Arthritis Index (WOMAC)  knee pain scale  36-item Short Form Survey (SF-36)  global change  **Functional**: Quad strength  Balance test  **Time points**: 12 week intervention duration   1. Baseline 2. Post treatment 3. 24 weeks | **Pain**:  Similar pain reductions at 12 weeks and 24 weeks.  **Disability**:  Both groups showed similar improvements on all disability outcomes.  **Functional**:  Global improvement reported by similar amounts in both groups (not statistically significant).  There was no change in either group or quad strength. | Physical therapy program was no more effective than regular contact with a PT at reducing pain and disability | 8 |
| Clark  (2000) [36] | **Age**: 16-40  **Diagnosis**: Anterior knee pain    **Inclusion**: pain > 3 months  **Exclusion**: history of true locking, patella dislocation, arthritis, any knee radiographic abnormality, ligament laxity (medial and lateral collateral ligament or anterior draw test), malignancy, infection, or previous knee physiotherapy | N = 80 | **T_1_**: exercise, McConnell tape, education  (n=20)  **T_2_**: McConnell tape and education  (n=20)  **T_2_**: exercise and education  (n=20)  **C**: education alone (n=20) | **Pain**:  VAS  **Disability**:  WOMAC  Hospital and Anxiety Depression scale  **Functional**: Patient satisfaction  **Time points**: 3 month intervention duration   1. Baseline 2. 3 months 3. 12 months | **Pain**:  All groups improved significantly in VAS though between groups did not differ  **Disability**:  All groups improved significantly in WOMAC,  and anxiety scores on the Hospital and Anxiety Depression scale, though between groups did not differ  **Functional**:  Quadriceps strength improved in all groups, but appeared to improve more in groups with exercising and education rather than tape alone (approached significance) | Muscle stretching and strengthening had a beneficial effect at three months post physiotherapy. Taping did not influence the outcome. | 7 |
| Cushnaghan (1994) [45] | **Age**: 55-84 described in the methods only in reference to the demographics of the described patient otherwise no age criteria was stated prior to enrollment  **Diagnosis**: Knee osteoarthritis    **Inclusion**: anterior knee pain, difficulty walking/with steps and stairs, radiographic evidence of osteoarthritis  **Exclusion**: none described | N = 14 | **T_1_**: neutral (over front of patella without pressure)  **T_2_**: medial taping (McConnell)  **T_3_**: lateral taping  **Subgroup sample size not available in study | **Pain**:  VAS  **Disability**: n/a  **Functional**: Rating of change with each treatment  Tape preference  **Time points**: 4 day intervention duration   1. Baseline 2. 1 hour after application 3. Post treatment then crossover | **Pain**:  Medial tape resulted in a 25% reduction in knee pain and was statistically significant  **Functional**:  Medial taping was better for symptom change, and tape preference | Medial patella taping's results on pain reduction were statistically and clinically significant compared to neutral and lateral taping at all time points except for one hour and one day. Medial patella taping resulted in reduced pain in patients with knee osteoarthritis. | 4 |
| Dhanakotti (2016) [46] | **Age**: 40-65  **Diagnosis**: knee osteoarthritis  **Inclusion**: pain at least in one knee >4 weeks duration, diagnosis of knee osteoarthritis by an orthopedic physician, radiographic evidence of grade II & III of Kellgren and Lawrence criteria for knee osteoarthritis, tibiofemoral arthritis  unilateral involvement, pain intensity level between 6-8 point of NPRS, able to walk independently  without any assistive devices, can ascend and descend at least a flight of stairs  **Exclusion**: acute inflammation, contracture or surgery affecting the knee, score <20 on the mini-mental state examination, pain with exercise, unstable medical conditions within the past 6 months, non-steroidal anti-inflammatory drugs (NSAIDs), injection or physical therapy within last 3 months in knee joint, BMI > 30, secondary osteoarthritis, skin allergy, local skin lesion or infection | N = 30 | **T_1_**: KT + conventional physical therapy  (n=15)  **C**: conventional physical therapy (stretching and strengthening)  (n=15) | **Pain**:  NPRS  **Disability**:  WOMAC  **Functional**:  Quadriceps strength  **Time points**: 3 week intervention   1. Baseline 2. Post-treatment | **Pain**:  Both groups improved however, there was statistical significance favoring the addition of KT  **Disability**:  Both groups improved however, there was statistical significance favoring the addition of KT  **Functional:**  Both groups improved however, there was statistical significance favoring the addition of KT | The addition of KT to to conventional physiotherapy reduced pain, improved quadriceps  strength and knee functional ability in  knee osteoarthritis. | 7 |
| Ghourbanpour (2018) [37] | **Age**: 20-50  **Diagnosis**: patellofemoral pain syndrome  **Inclusion**: anterior knee pain >1 month, discomfort and pain during palpation of the medial and lateral side of patella, worsening of symptoms during prolonged sitting,  climbing stairs, squatting, running, skipping and jumping, positive Apprehension and Clark tests, feeling pain during resistive knee extension  **Exclusion**: history of dislocation and direct trauma to the patella, any  rheumatologic conditions, diabetes, any trauma or meniscus injury, ligamentous instability, referred pain from the lumbar spine, hip, pelvis and sacroiliac regions, remarkable knee joint inflammation and effusion, knee surgery, knee physiotherapy, steroid injection | N = 30 | **T_1_**: McConnell Patellar Taping + conventional  (n=15)  **C**: conventional (quadriceps strengthening, hamstring stretching)  (n=15) | **Pain**:  VAS  **Disability**:  KOOS  **Functional**: n/a  **Time points**: 4 week intervention   1. Baseline 2. Post-treatment | **Pain**:  Both groups showed significant reduction in pain intensity compared to baseline, however, no difference between groups  **Disability**:  No significant differences between groups post-treatment. | Conventional treatment with and without patellar taping appear to have a positive impact on pain reduction and quality of life in patients with patellofemoral pain syndrome.  Adding McConnell patellar taping to conventional physiotherapy has no more additional effects than conventional physiotherapy alone. | 4 |
| Han  (2018) [47] | **Age**: not specifically described  **Diagnosis**: knee osteoarthritis    **Inclusion**: 6≥VAS  **Exclusion**: other treatment or therapy for pain alleviation during the study period, pain or depression  medications | N = 32 | **T_1_**: Elastic tape + routine treatment  (n=16)  **C**: routine treatment (thermotherapy, electrotherapy)  (n=16) | **Pain**:  VAS  **Disability**:  Back Depression Inventory (BDI)  **Functional**: n/a  **Time points**: 4 week intervention duration   1. Baseline 2. Post-treatment | **Pain:**  The taping group had a significant improvement in VAS  .  **Disability:**  The taping group had a significant improvement in BDI | Taping showed an immediate effect in decreasing knee pain and depression among patients  with degenerative arthritis. | 5 |
| Harrison (1999) [38] | **Age**: 12-35  **Diagnosis**: Patellofemoral pain syndrome    **Inclusion**: two of the following criteria: (1) patellar pain with manual compression of the patella on the femur, (2) patellar tenderness with palpation of the posterior-medial and posterior-lateral borders of patella; (3) patellar pain during resisted knee extension, (4) patellar pain with manual compression during isometric knee extensor contraction  **Exclusion**: other musculoskeletal conditions of the knee, previous or pending knee surgery, gross knee effusion, knee pain referred from the hip or spine, upper or lower motor neuron lesions, history of steroid injections to the knee | N = 113 | **T_1_**: home strengthening and flexibility program (n=42)  **T_2_**: similar exercise program monitored by PT  (n=34)  **T_3_**: exercises, McConnell patellar taping, biofeedback  (n=36) | **Pain**:  VAS  **Disability**:  Patellofemoral scale  Step test  **Functional**: Functional Index Questionnaire  Subjective clinical change measure  **Time points**: 1 month intervention   1. Baseline 2. 1 month 3. 3 months 4. 6 months 5. 1 year | **Pain**:  Significant improvements in step test pain threshold for all groups with no between group differences  **Disability**:  No significant between group differences in the PF scale  **Functional**:  At one month, group three showed significantly higher function and lower pain scores than group 2, but by one year, no differences between groups existed | A modified program including taping and biofeedback may result in a quicker improvement, but long term results remain very similar. | 5 |
| Hinman (2003) [48] | **Age**: >50  **Diagnosis**: Knee osteoarthritis    **Inclusion**: clinical and radiological classification criteria  of the American College of Rheumatology (presence  of osteophytes,  **Exclusion**: allergy to tape or history of joint replacement, symptoms or signs suggestive of another cause of knee pain, physiotherapy for the knee (previous six months), body mass index > 38, rheumatoid arthritis, steroid injection or knee surgery in previous six months, history of knee taping, and fragile skin around the knee. | N = 87 | **T_1_**: Therapeutic tape (rigid)  (n=29)  **T_2_**: control tape (n=29)  **T_3_**: no tape  (n=29) | **Pain**:  VAS  **Disability**:  WOMAC  Disability VAS  **Functional**: n/a  **Time points**: 3 week intervention   1. Baseline 2. Post treatment 3. 6 week follow up | **Pain**:  Therapeutic tape had significantly greater reduction in pain on most secondary outcomes than no tape group  **Disability**:  Therapeutic tape had significantly greater reduction in disability on most secondary outcomes than no tape group | Therapeutic knee taping is efficacious treatment for the management of pain and disability in patients with knee osteoarthritis. | 8 |
| Ibrahim  (2015) [49] | **Age**: 30-50  **Diagnosis**: knee osteoarthritis  **Inclusion**: unilateral, chronic, diagnosed by an orthopedist as having grade II knee osteoarthritis with the presence of knee pain, osteophytes and definite joint space narrowing based on the American College of Rheumatology criteria  **Exclusion**: rheumatoid arthritis, intra-articular steroid injection, knee surgery, neurological condition affecting lower limbs, use of assistive devices for walking | N = 60 | **T_1_**: KT + traditional exercise  (n=15)  **T_2_**: Sensorimotor training + traditional exercise  (n=15)  **C**: Traditional exercise  (n=15) | **Pain**:  VAS  **Disability**:  Arthritis impact functional assessment scale  **Functional**:  Proprioceptive acuity (dynamometer)  **Time points**: 8 week intervention   1. Baseline 2. Post-treatment | **Pain**:  The KT group showed a  highly significant reduction in the pain intensity compared with traditional exercise and sensorimotor training groups  **Disability**:  The sensorimotor group demonstrated greater improvement in disability compared to KT and traditional exercise  **Functional:**  Proprioceptive accuracy was statistically significant at post-treatment in favor of the sensorimotor group | Sensorimotor training is most efficient modality producing  more positive effects on proprioception and functional activity level than KT for treating patients with knee osteoarthritis. | 3 |
| Kachanathu (2017) [61] | **Age**: 20-30  **Diagnosis**: tibial stress syndrome (shin splints)  **Inclusion**: hyperpronated foot (navicular drop 10 mm), atraumatic history of >1 week of medial tibial pain exacerbated by running, presence of at least 10 cm of diffuse palpatory tenderness at the distal ⅔ of the posteromedial aspect of the leg with a positive provocative test, pain during forced passive ankle dorsiflexion, pain during active ankle plantar flexion against resistance  **Exclusion**: stress fractures, bone tumors, compartment syndrome, congenital anomalies, vascular insufficiencies, known allergy to tape, other injuries to the lower extremities that required reduced activity, treatment from another health care practitioner | N = 40 | **T_1_**: KT + stretching and strengthening exercises  (n=20)  **C**: Orthotics + stretching and strengthening exercises  (n=20) | **Pain**:  VAS  **Disability**: n/a  **Functional**:  6-m single-leg distance hop test  Navicular drop test  **Time points**:1 week intervention duration   1. Baseline 2. Post-treatment | **Pain**:  The KT groups demonstrated significant improvement in the compared to the orthotics  **Functional**:  Significant changes in hop distance was observed in the taping group post-treatment, while navicular drop test values improved in both groups without significant difference | KT plays a vital role in improving functional outcomes compared to orthotics by reducing pain and improving functional  activity in patients with shin splint; however, navicular drop correction was insufficient after  both interventions. | 3 |
| Kaya Mutlu (2017) [50] | **Age**: not specifically described  **Diagnosis**: Knee osteoarthritis  **Inclusion**: diagnosis of osteoarthritis of the knee according to the American College of Rheumatology  criteria  **Exclusion**: rheumatoid arthritis,  previous knee or hip joint replacement surgery of the affected joint, any surgical procedure on the lower limbs within the previous year, a planned surgical procedure on the lower limbs within the next 6 months, opioid analgesia or corticosteroid or analgesic injection interventions for knee pain within  the previous 6 months, physical therapy intervention on the lower limbs in the previous 6 months, and uncontrolled hypertension, moderate to high risk of cardiac complications  during exercise | N = 42 | **T_1_**: KT  (n=21)  **T_2_**: Placebo taping  (n=21) | **Pain**:  VAS  **Disability**:  WOMAC  **Functional**:  Aggregated Locomotor Function  Range of motion (ROM)  Muscle strength  **Time points**: 12-16 day intervention   1. Baseline 2. After initial application 3. After 3rd application 4. 1 month follow-up | **Pain**:  KT group demonstrated  a significant decrease in the VAS activity compared with placebo group from the initial taping application through 1 month follow-up  **Disability**: No significant changes between groups.  **Functional**:  KT group demonstrated  a significant decrease in the walking task of the Aggregated Locomotor Function compared with placebo group from the initial taping application through 1 month follow-up | The KT group demonstrated statistically significant improvements in  pain levels during activity and walking task and these effects were observed after the 1-month follow-up.  The VAS night score and knee flexion ROM in the KT group were also improved after  the 1-month follow-up. | 8 |
| Kocyigit (2015) [51] | **Age**: 30-70  **Diagnosis**: knee osteoarthritis  **Inclusion**: knee pain diagnosed as knee osteoarthritis according to  clinical diagnostic criteria proposed by American College of Rheumatology criteria, VAS between 20 and 70 mm,  **Exclusion**: previous knee fracture, surgery in last six months, previous or concurrent diagnosis of cruciate and collateral ligament tear, presence of acute inflammation findings in the involved knee, inflammatory joint disease, history of electrotherapy or injection for the knee in the last 3 months, previous application of KT | N = 43 | **T_1_**: KT  (n=22)  **T_2_**: Sham taping  (n=21) | **Pain**:  VAS with activity  VAS at night  **Disability**:  Nottingham Health Profile (NHP)  **Functional**:  Lequesne index  **Time points**: 12 day intervention   1. Baseline 2. Post-treatment | **Pain**:  VAS for activity and nocturnal pain decreased significantly after treatment for both groups  **Disability**:  NHP pain and activity scores decreased significantly after treatment for both groups. NHP energy score favored sham taping at day 12.  **Functional**:  Lequense index scores decreased significantly after treatment for both groups. | Inconclusive  evidence of a beneficial effect of KT over sham taping in knee  osteoarthritis.  Taping may represent an effective adjunct therapy in the management  of knee osteoarthritis patients. | 8 |
| Kowall  (1996) [39] | **Age**: 14-40  **Diagnosis**: unilateral or bilateral patellofemoral pain syndrome  **Inclusion**: pain > 1 month, unilateral or bilateral patellofemoral pain  **Exclusion**: history of patellofemoral dislocation, synovial plicae, or meniscal or ligamentous injury, previous knee trauma or surgery | N = 25 | **T_1_**: standard physical therapy program  (n=13)  **T_2_**: standard program and McConnell taping (n=12) | **Pain**:  VAS: frequency, severity, pain on athletic events, effect on activities of daily living (ADLs)  **Disability**: n/a  **Functional**: isokinetic strength  Electromyography (EMG) activity  **Time points**: 4 week intervention  1. Baseline  2. Post-treatment | **Pain:**  Both groups significantly reduced in pain, but not significantly different  **Functional:**  Both groups improved in EMG activity, but not statistically significantly | A standard physical therapy program seems to show benefits for patients with patellofemoral pain, but taping gives no additional benefits. | 4 |
| Malgaonkar (2014) [52] | **Age**: 50-60  **Diagnosis**: knee osteoarthritis  **Inclusion**: history of knee osteoarthritis for two years, grade 3 changes on radiological evaluation using Kellgren and Lawrence system, three of the criteria plus knee pain based on American college of rheumatology criteria for classification and reporting of osteoarthritis of knee joint (age >50 years, stiffness <30 min, crepitus, bony tenderness, bony enlargement, no palpable warmth), male and female subjects  **Exclusion**: osteoarthritis of hip, ankle and foot, serious concomitant systemic disease, Intra-articular corticosteroid or hyaluronic injection in past six months, lower limb surgery, osteoarthritis of knee with deformity, on medicine such as analgesics for knee pain | N = 40 | **T_1_**: KT  (n=20)  **C**: Movement with mobilization  (n=20) | **Pain**:  VAS  **Disability**:  WOMAC  **Functional**: n/a  **Time points**: 2 week intervention   1. Baseline 2. Post-treatment | **Pain**:  Significant improvement in mean VAS was observed in both groups, however there was no significant difference between groups post-treatment  **Disability**:  Significant improvement in disability was observed in both groups, however there was no significant difference between groups post-treatment | Both Movement with Mobilization and KT significantly improve pain and functional disability in the short-term for subjects with osteoarthritis of the knee joint. | 4 |
| Mason  (2011) [40] | **Age**: 13-82  **Diagnosis**: Patellofemoral pain syndrome    **Inclusion**: pain > 1 month, pain around or behind patella with 2 or more of following: prolonged sitting, squatting, kneeling, ascending or descending stairs, running  **Exclusion**: patellar tendinitis,  Osgood-Schlatter disease or hip osteoarthritis, meniscal symptoms, required surgery, rheumatoid symptoms, synovitis, back pain, tibiofemoral osteoarthritis | N = 60 | **T_1_**: Infrapatellar taping (McConnell) (n=15)  **T_2_**: Quadriceps strengthening (n=15)  **T_3_**: Quadriceps stretching  (n=15)  **C**: Control  (n=15)  ** Following one week of single modality treatment, all four groups received one week of treatment  combining taping, quadriceps strengthening and quadriceps stretching | **Pain**:  VAS in 4 activities (going up stairs, down stairs, stepping down large step, self-selected activity  **Disability**: n/a  **Functional**:  Quadriceps isokinetic strength  Quadriceps length  Pain-free eccentric knee control angle Compliance  **Time points**: 2 week intervention   1. Baseline 2. 1 week (one treatment) 3. 2 weeks (all treatments) | **Pain:**  For taping, no significant changes in pain during stair climbing up and down stairs, the step down test or in quadriceps strength while pain during a self-selected activity, had improved significantly following one week of patellar taping  **Functional:**  For taping, no significant in quadriceps strength while, quadriceps length had improved significantly following one week of patellar taping  ********  Statistically significant changes in two of seven measures for the taping group, five of seven for strengthening group, five of seven for the stretching group, and none in the control group. When all three modalities were combined for one week, all seven measures improved significantly. | Quadriceps stretching and strengthening resulted in more improvements than taping for the treatment of patellofemoral pain.  Combining these treatments is recommended to treat patellofemoral pain. | 6 |
| Miller  (2013) [41] | **Age**: 18-20  **Diagnosis**: unilateral anterior knee pain  **Inclusion**: pain >2 weeks, 3 of the following: pain with running, stair climbing, squatting, sitting, knee flexion  **Exclusion**: knee tendinopathy, lower extremity fracture, knee or ankle ligamentous injury, conditions affecting balance (eg, concussion), osteoarthritis, lower extremity or back surgery (including arthroscopy), patellar subluxation /dislocation, or neurologic deficit | N = 18 | **T_1_**: KT  (n=6)  **T_2_**: lumbopelvic manipulation  (n=6)  **T_3_**: control taping [strip of KT tape in improper placement]  (n=6) | **Pain**:  VAS (rest, squatting, Y-balance)  **Disability**:  Lower extremity functional scale  **Functional**: Y-balance test  Squatting ROM  **Time points**: 3 day intervention   1. Baseline 2. Immediately after intervention 3. Post-treatment | **Pain:**  No significant differences were observed between groups for VAS with squatting or VAS with the Y-balance  **Disability:**  No significant differences were observed between groups for lower extremity functional scale  **Functional:**  KT group performed better at Y-balance test and squatting ROM than lumbopelvic manipulation and control groups  KT and lumbopelvic performed significantly better than the control at double-leg squatting ROM. | KT may improve gluteus medius activation and lumbopelvic manipulation may also immediately improve patellofemoral pain syndrome rehabilitation programs. | 6 |
| Öğüt  (2018) [53] | **Age**: 50-60  **Diagnosis**: knee osteoarthritis    **Inclusion**: female, knee osteoarthritis according to American College  of Rheumatology diagnostic criteria, Kellgren-Lawrence grade 2 or 3  **Exclusion**: history of lower extremity surgery, any diagnosis of musculoskeletal disease other than osteoarthritis, Kellgren-Lawrence grade 1 or 4, intra-articular knee joint injections <6 months, lower extremity sensory or strength deficits, cognitive impairment, open wound or skin lesions in the treatment area, skin allergies | N = 61 | **T_1_**: KT with tension + modalities  (n=31)  **T_2_**: Sham KT + modalities (hot pack, TENs, ultrasound, isometric exercise)  (n=30) | **Pain**:  VAS  **Disability**:  WOMAC  **Functional**: Active ROM (AROM)  50 meter walking time  Isokinetic muscle strength  **Time points**: 3 week intervention duration   1. Baseline 2. Post treatment 3. 1 month 4. 3 months | **Pain**:  Significant improvement was observed in the KT group compared to sham-KT group immediately after treatment at 1 month follow-up  **Disability**:  Significant improvement was observed in the KT group compared to sham-KT group immediately following treatment  **Functional**:  Significant differences were determined in both groups in  the zero, one, and three-month values when compared to baseline values | The application of KT to females with knee OA appears to be a method that may be effective on pain and functional capacity. | 5 |
| Parekh  (2018) [54] | **Age**: 40-60  **Diagnosis**: knee osteoarthritis    **Inclusion**: knee pain  **Exclusion**: fracture or injury, carcinoma, systemic illness, subject in other ongoing research study | N = 50 | **T_1_**: McConnell + exercise  (n=25)  **T_2_**: exercise (straight leg  raise, straight leg abduction, straight leg extension, last degree extension, and static quadriceps)  (n=25) | **Pain**: n/a  **Disability**:  WOMAC  **Functional**: Timed Up and Go test (TUG)  **Time points**: 1 week intervention duration   1. Baseline 2. Post treatment | **Disability**:  Statistically significant improvement occurred in both groups for WOMAC, though favored the taping group  **Functional**:  Significant changes in TUG value favored the taping group | Taping is effective individuals diagnosed with knee osteoarthritis to improve the functional outcome of patients in the short term. | 3 |
| Quilty  (2003) [55] | **Age**: not specifically described  **Diagnosis**: knee osteoarthritis    **Inclusion**: chronic knee pain, patellofemoral joint osteophytes  **Exclusion**: advanced radiographic changes of hip or tibiofemoral joint osteoarthritis, history of major knee surgery, fractures involving the knee joint, rheumatoid arthritis, previous patellar taping, currently receiving treatment from a physiotherapist for knee problems | N = 87 | **T_1_**: Standard physiotherapy and taping  (n=43)  **T_2_**: standard physiotherapy (n=44) | **Pain**:  VAS  **Disability**:  WOMAC  **Functional**: Quadriceps strength  **Time points**: 10 week intervention   1. Baseline 2. 5 months 3. 12 months | **Pain**:  Knee pain at either at 5 month and 12 month improved, but did not show statistically significant differences between the groups  **Disability**:  No significant differences between groups at 5 months or 12 months.  **Functional**:  Significant increase in quadriceps strength in taping group at 5 months, but at 12 months, no longer significant. | Small improvements in pain and quadriceps strength post-intervention, but these between group differences disappear after 12 months. | 8 |
| Rinkle  (2010) [56] | **Age**: 40 and older  **Diagnosis**: chronic knee osteoarthritis    **Inclusion**: pain >1 year, Kellgren and Lawrence grade III radiographic evidence for osteoarthritis knee unilateral or bilateral, average knee pain 3/10 or greater  **Exclusion**: acute exacerbation in or around joint, traumatic injury to knee joint within 6 months | N = 60 | **T_1_**: McConnell patellar taping and conventional treatment  (n=20)  **T_2_**: Mulligan's Mobilisation with movement and conventional treatment  (n=20)  **T_3_**: McConnell patellar taping, Mulligan's mobilisation with movement, and conventional treatment  (n=20) | **Pain**:  VAS  **Disability**:  WOMAC  **Functional**: knee flexion range of motion  **Time points**: 2 week day intervention   1. Baseline 2. Post-treatment | **Pain**:  All groups showed statistically significant improvements in pain  **Disability**:  All groups showed statistically significant improvements on WOMAC  **Functional**:  All groups showed statistically significant improvements in active and passive ROM but the combination of McConnell taping and Mulligan mobilisation produced statistically significant better results than the other two separate therapies in all measures. | Combining McConnell patellar taping and Mulligan mobilisation with movement may result in improved range of motion, as well as decreased pain and disability in patients with chronic knee osteoarthritis. | 4 |
| Rood  (2012) [60] | **Age**: > 18  **Diagnosis**: Primary lateral patellar dislocation    **Inclusion**: presented at emergency department  **Exclusion**: previous knee operations, osteochondral fracture of the knee or neurologic disorders, previous patellar dislocation or other abnormality to one of both knees | N = 18 | **T_1_**: taping + pressure bandage and dorsal long leg split for one week.  (n=9)  **T_2_**: cylinder cast immobilization + pressure bandage and dorsal long leg split for one week. (n=9) | **Pain**: n/a  **Disability**:  Lysholm Knee Scoring Scale  **Functional**: Redislocation rate  **Time points**: 6 week intervention duration   1. Baseline 2. 1 week 3. 6 weeks 4. 12 weeks 5. 1 year 6. 5 year | **Disability:**  Taping resulted in significant differences in the Lysholm score at 6 weeks, 12 weeks, and 5 years  **Function:**  Knee function was also better in the taping group at the 1 year follow up as well as no cases of recurrent dislocation in either group | Tape bandage immobilization appears to be superior to a cylinder cast even after 5 years for knee dislocation patients. | 5 |
| Sedhom (2016) [57] | **Age**: 40-60  **Diagnosis**: knee osteoarthritis  **Inclusion**: female, BMI ranged from 25-35 kg/m2, grade 2 and 3 according to Kellgren and Lawrence radiological classification of osteoarthritis  **Exclusion**: prior knee operation, recent knee joint injury, vascular disease (atherosclerosis), history of intra-articular steroid or hyaluronic acid injections in the last 6 months | N = 40 | **T_1_**: KT + physical therapy therapy  (n=20)  **C**: Aescin, Diethyl-amine Salicylate gel with pulsed ultrasound + physical therapy  (n=20) | **Pain**:  VAS  **Disability**: n/a  **Functional**:  ROM  Proprioception  **Time points**: 4 week intervention   1. Baseline 2. Post-treatment | **Pain**:  A significant difference was observed between groups in favor of pulsed ultrasound  **Functional:**  No significant difference between groups in for  proprioceptive accuracy and knee flexion ROM | Aescin, Diethyl-amine Salicylate gel PH with the selected exercise program is more effective than KT application in relieving knee pain  in knee osteoarthritic patients | 4 |
| Sudhesh (2013) [58] | **Age**: > 45  **Diagnosis**: Knee osteoarthritis    **Inclusion**: Unilateral  **Exclusion**: none stated | N=30 | **T_1_**: taping (McConnell) and closed kinetic chain (n=15)  **C**: control  (n=15) | **Pain**: n/a  **Disability**:  WOMAC  **Functional**: Q-angle  **Time points**: intervention duration   1. Baseline 2. 3 weeks | **Disability:**  Taping group has comparatively higher reduction is pain and stiffness than in control group.  Taping group has more functional improvement than in control group  **Function:**  No significant change in Q-angle in Group A. Group B is no difference in Q angle | Significant reduction in pain and stiffness as well as improved functional activities with taping and closed kinetic chain exercises.  Q angle had no difference in either group for knee osteoarthritis. | 5 |
| Wageck (2016) [59] | **Age**: >60  **Diagnosis**: tibiofemoral osteoarthritis  **Inclusion**: 4 or more out of 10 VAS, knee joint stiffness during standing activities, stiffness that had been present for at least 6 months at screening, pain during passive mobilisation of the knee, intermittent swelling, radiographic signs of joint degeneration. Any grade of the Kellgren-Lawrence  scale was permissible.  **Exclusion**: systemic rheumatic  diseases, history of surgery in the affected limb, presence of any  other injuries in the affected limb (bone, muscle or skin), history of  skin allergy, and any other condition that could affect assessments | N = 76 | **T_1_**: KT  (n=38)  **C**: Sham taping  (n=38) | **Pain**:  VAS  Pressure pain threshold (PPT)  **Disability**:  WOMAC  **Functional**:  Isokinetic muscle strength  Perimetry  Volumetry  Lysholm Knee Scoring Scale  **Time points**: 4 day intervention duration   1. Baseline 2. Post-treatment Day 4 3. Day 19 | **Pain**:  No difference between the  KT and sham group for VAS or PPT at the end of the 4-day intervention period, or 15 days.  **Disability**:  No difference between the  KT and sham group for WOMAC at the end of the 4-day intervention period, or 15 days.  **Functional**:  No difference between the  KT and sham group for Lysholm Knee scoring scale, isokinetic muscle strength, perimetry or volumetry at the end of the 4-day intervention period, or 15 days. | People with knee osteoarthritis did not  experience any significant benefits in pain, swelling, quadriceps  strength, knee function or knee-related health status from  KT applied for four days. | 8 |
| Whittingham (2004) [42] | **Age**: 17-25  **Diagnosis**: Patellofemoral pain syndrome    **Inclusion**: Army recruit, 2 of the 4: pain on ascending/descending stairs, squatting, sitting for extended periods of time, increase in physical activity  **Exclusion**: history of subluxation or dislocation of the patella, anterior or posterior cruciate ligament insufficiency, previous knee surgery or meniscal damage, or any other underlying musculoskeletal problems that would have prevented the subject from performing the exercises | N = 30 | **T_1_**: patella taping [McConnell] with standardized exercise  (n=10)  **T_2_**: placebo patella taping with standardized exercise  (n=10)  **T_3_**: exercise program alone (n=10) | **Pain**:  VAS (24 hour average, with aggravating activity, step test)  **Disability**:  Functional Index Questionnaire  **Functional**: n/a  **Time points**: 4 week intervention   1. Baseline 2. 1 week 3. 2 weeks 4. 3 weeks 5. 4 weeks | **Pain**:  Combined taping and standardized exercises group had significantly lower 24 hour VAS ratings than both the placebo taping and exercise group and the exercise-alone group at weeks 2, 3, and 4  With step test VAS, Combined taping and standardized exercise group had significantly lower VAS score than both the placebo taping and exercise group and the exercise-alone  group at weeks 2, 3, and 4.  **Disability**:  Significantly better for taping-and-exercise than placebo taping-and-exercise and exercise only. No significant differences between placebo-taping-and-exercise and exercise only . | Daily patella taping and exercises superior to placebo taping and exercises or exercise alone in improving pain and function with patellofemoral pain syndrome. | 8 |

**APPENDIX 2B: EVIDENCE TABLE FOR RANDOMIZED CONTROLLED TRIALS OF THE ANKLE**

| Study | Population description | Sample size | Interventions | Outcomes/time points | Results | Key author conclusions | PEDro Score |
| --- | --- | --- | --- | --- | --- | --- | --- |
| Ardèvol (2002) [62] | **Age**: < 35  **Diagnosis:** First phase of grade III tear of anterior talofibular ligament or anterior talofibular ligament plus calcaneofibular ligament  **Inclusion**: closed physeal growth plates, habitually practiced a sport for a minimum of 4 hour per week  **Exclusion**: previous injury to the same ankle or who had associated injuries (fractures, avulsion) | N= 121 | **T_1_**: immobilization with below-knee plaster cast  (n=57)  **T_2_**: strapping and cryotherapy (n=64) | **Pain**: n/a  **Disability**: n/a  **Functional**: Reduction in objective laxity  Late symptoms of injury  Reinjury  Sporting level upon return  Time to return  **Time points**: 21 days intervention   1. Baseline 2. 3 months 3. 6 months | **Functional**:  Strapping group showed significantly earlier and better return to physical activity, fewer symptoms at 3 and 6 months, but these differences between groups disappeared at 12 months. Strapping treatment also showed a better decrease in joint laxity | Functional treatment is safe, associated with a more rapid recovery, and suitable in athletic populations. | 5 |
| Johannes (1993) [63] | **Age**: 12-60  **Diagnosis**: ligament injuries to one ankle  **Inclusion**: acute injury to one ankle, persistent symptoms and signs after 4-8 days of compressive bandage, rest and elevation, Grade I-III ankle sprain  **Exclusion**: fractures, concomitant injuries, pre-existing abnormalities | N=136 | **T_1_**: semi rigid bandage ('Scotchrap') (n=59)  **T_2_**: standard adhesive tape (n=57) | **Pain**:  Pain (unspecified)  **Disability**: n/a  **Functional**:  Mobility (unspecified) Patient satisfaction  **Time points**: intervention duration   1. Baseline 2. 2 weeks 3. 4 weeks | **Pain**: No significant differences between groups for pain  **Functional**:  No significant differences between groups with mobility.  No significant differences between patient satisfaction. | Scotchrap can be just as effective as adhesive taping for the management of ankle sprains. The length of time it takes to apply is slightly higher, but the effects remain without reapplication and can also be used for individuals allergic to tape. | 4 |
| Lardenoye (2012) [64] | **Age**: 16-55  **Diagnosis**: Grade II or III ankle sprain  **Inclusion**: grade II or III ankle sprain or anterior drawer instability (grade III) within 5-7 days of injury  **Exclusion:** grade I ankle sprain, undergoing preventative treatment of recurrent ankle sprains, previous ankle sprain or fracture, sustained swelling that made treatment with tape impossible, mentally disabled | N = 100 | **T_1_**: taping  (n=50)  **T_2_**: bracing (n=50) | **Pain**:  Verbal rating scale (poor=5; excellent=1)  **Disability**: n/a  **Functional**:  Ankle joint function/Karlsson ROM  **Time points**: 4 week intervention   1. Baseline 2. 2 weeks 3. Post-treatment | **Pain**:  Pain score was similar between tape and brace treatments.  **Functional**:  Ankle joint function increased significantly during 4 weeks of treatment and after 8 weeks; no difference between groups. | Semi-rigid brace leads to less complications and higher patient satisfaction with tape in the treatment of acute lateral ankle sprain. No difference regarding functional outcome and pain. | 7 |
| Mickel (2006) [65] | **Age**: not specifically described  **Diagnosis**: ankle sprain  **Inclusion**: high school junior varsity and varsity football players with stable, uninjured ankles  **Exclusion**: current complaints related to either ankle | N = 93 | **T_1_**: AirSport Ankle Brace  (n=48)  **T_2_**: adhesive tape in closed basket weave with figure-of-eight heel lock  (n=45) | **Pain**: n/a  **Disability**: n/a  **Functional**: Number of ankle sprains  Exposures (games and practices)  **Time points**: 23 practices and games   1. Baseline 2. 23 games | **Functional**:  Both groups suffered the same amount of ankle sprains showing not significant between group differences | As the brace was less expensive and time consuming and the results were equal, bracing can be a substitute for adhesive taping to prevent ankle sprains. | 4 |
| Nunes  (2015) [68] | **Age**: not specifically stated  **Diagnosis**: lateral ankle sprain  **Inclusion**: sprain within 48 to 96 hours of assessment, visible swelling of the ankle  **Exclusion**: had a fracture; had an open wound; had systemic lower limb swelling related to cardiac, kidney or venous diseases; or were suspected to be pregnant | N = 36 | **T_1_**: KT  (n=18)  **C**: sham taping  (n=18) | **Pain**: n/a  **Disability**: n/a  **Functional**:  Volumetry  Perimetry  **Time points**: 3 day intervention   1. Baseline 2. Post-treatment 3. 15 days | **Functional**:  No difference between groups for volumetry, perimetry or relative volumetry  No effect of KT after 3 or 15 days when injured and non-injured sides were compared | The application of KT with the aim of stimulating the lymphatic system is not effective in decreasing acute swelling after an ankle sprain in athletes. | 8 |
| Simon  (1969) [5] | **Age**: no specifically stated  **Diagnosis**: ankle sprain  **Inclusion**: SUNY Buffalo varsity football squad  **Exclusion**: history of chronic ankle problems | N =148 | **T_1_**: Louisiana Wrap  (n=75)  **T_2_**: taped using double stirrups, double figure-eights, medial and lateral heel locks  (n=73) | **Pain**: n/a  **Disability**: n/a  **Functional**:  Days of practice attended  Ankle injuries  **Time points:** Football season   1. Baseline 2. End of season | **Functional:**  No statistically significant difference between the two techniques on the rate of ankle injuries/practice days. | No difference between ankle taping or wrapping on the occurrence of ankle injuries in a relatively healthy population of athletes. | 1 |
| Specchiulli (2001) [66] | **Age**: < 40  **Diagnosis**: grade III lateral ankle ligament injury  **Inclusion**: closed epiphyseal growth plate, acute injury (<24 hours)  **Exclusion**: previous history of ankle instability | N = 100 | **T_1_**: surgical treatment and immobilization in non-weight bearing cast  (n=50)  **T_2_**: adhesive ankle taping (n=50) | **Pain**: n/a  **Disability**: n/a  **Functional**: 100 point ankle-hindfoot scale Interval before resumption of exercise duration  Residual functional instability  Atrophy of calf muscles  Return to sport at same level as before  **Time points**: 40 day intervention | **Functional**:  No significant difference in ankle-hindfoot scale.  Surgical patients returned to injury on average 10 weeks after injury compared to 7 weeks for the nonsurgical group (statistically significant)  No significant differences in the level of return to sport between groups.  No significant differences in swelling between groups. | No significant advantages to surgical treatments over non-surgical taping for lateral ankle Grade III ligament tears | 2 |
| van den Bekerom (2016) [67] | **Age**: ≥ 18 years  **Diagnosis**: Grades II or III ankle sprains  **Inclusion**: presentation to the emergency department < 72 h after the acute injury  **Exclusion**: history of chronic instability, fracture on conventional radiograph, other injuries or disabilities on the same limb, alcoholism, serious psychiatric and neurological illness, bilaterally sprained ankles, previous surgery on the lateral ankle ligaments, skin diseases where taping is contraindicated, neuromuscular disorders of the lower extremities, rheumatoid arthritis, gait disturbances | N = 193 | **T_1_**: tape  (n=66)  **T_2_**: semi-rigid brace  (n=58)  **T_3_**: lace-up brace  (n=62) | **Pain**:  VAS at rest  **Disability**:  Foot and Ankle Outcome Score  **Functional**: Karlsson scoring scale  Return to work  Return to sports  Tegner activity level  **Time points**: 6 weeks intervention   1. Baseline 2. 2 weeks 3. 4 weeks 4. 6 months | **Pain**:  No significant differences.  **Disability**:  Except for the difference in Foot and Ankle outcome score sport between the lace-up and the semi-rigid braces, there are no differences in any of the outcome measures after 6-month follow-up  **Functional**:  No significant differences | No difference in outcome between tape, semi-rigid brace and a lace-up brace 6 months after treatment of acute lateral ankle ligament injury. | 6 |

**APPENDIX 2C: EVIDENCE TABLE FOR RANDOMIZED CONTROLLED TRIALS OF THE FOOT**

| Study | Population description | Sample size | Interventions | Outcomes/time points | Results | Key author conclusions | PEDro Score |
| --- | --- | --- | --- | --- | --- | --- | --- |
| Abd El Salam  (2014) [69] | **Age**: 40-60  **Diagnosis**: unilateral plantar fasciitis  **Inclusion**: pain > 4 weeks duration, pain at plantar heel, worse when first standing or walking after rest and improved initially after first standing, but worsened after increasing activity  **Exclusion**: non-athletes, corticosteroid injection in 3 months prior | N = 30 | **T_1_**: ultrasound, calf stretching, medial arch support  (n=15)  **T_2_**: ultrasound, calf stretching, low-dye (rigid) taping (n=15) | **Pain**:  VAS  **Disability**:  Foot pain disability  **Functional**:  n/a  **Time points**: 3 week intervention   1. Baseline 2. Post treatment | **Pain**:  There were statistically significant reductions in pain in both groups, however, significantly better in the medial arch support group  **Disability**:  There were statistically significant improvements in foot pain disability in both groups, however, significantly better in the medial arch support group | Medial arch support is both more convenient than low-dye taping in the short-term management of pain and pain-related disability in plantar fasciitis. | 6 |
| Hyland (2006) [70] | **Age**: 18-65  **Diagnosis**: plantar heel pain  **Inclusion**: pain with first steps upon waking (>3/10), pain at heel or plantar midfoot, everted calcaneus ≥ 2 degrees  **Exclusion**: previous surgery or treatment for plantar fasciitis in  the previous 6 months and during the study period, history of ankle or foot fracture, congenital deformity of the foot or ankle, spasticity throughout the lower extremity, use of an assistive device for ambulation, bilateral plantar heel pain | N = 41 | **T_1_**: stretching alone  (n=10)  **T_2_**: calcaneal low dye (rigid) taping only  (n=11)  **T_3_**: sham taping (n=10)  **C**: control  (n=10) | **Pain**:  VAS  **Disability**:  n/a  **Functional**: Patient specific functional scale  **Time points**: 1 week intervention   1. Baseline 2. Post treatment | **Pain**:  Within-group analysis revealed a significant difference in VAS score from pretreatment to post treatment for the stretching, calcaneal taping, and sham taping. No significant difference between stretching and sham taping or between control and sham taping  **Functional**:  Significant change pretreatment to post treatment for the control group in PSFS, but not significantly in other groups | Calcaneal taping was shown to be more effective for the relief of plantar heel pain than stretching, sham taping, or no treatment. | 4 |
| Lynch  (1998) [71] | **Age**: not specifically stated  **Diagnosis**: Plantar fasciitis  **Inclusion**: pain upon rising in the morning or after rest  **Exclusion**: history of trauma to the heel within previous 3 months, professional treatment [arch supports, heel cups, injections, or NSAIDS] within 1 month | N= 103 | **T_1_**: anti-inflammatory therapy [injection and capsules]  (n=35)  **T_2_**: accommodative heel cup and acetaminophen (n=33)  **T_3_**: orthoses and low-dye strapping  (n=35) | **Pain**:  VAS  **Disability**:  effect of pain on work, leisure, and exercise  **Functional**: none  **Time points**: 12 weeks intervention   1. Baseline 2. 2 weeks 3. 4 week 4. 6 weeks 5. 12 weeks | **Pain**:  Statistically significant differences in final VAS (45% of anti-inflammatory progressed to VAS between 0 and 2; 23% of accommodative; 64% of mechanical)  **Disability**:  No statistically significant differences with heel pain in work, exercise, leisure activities or in first-step pain. | Mechanical therapy, including orthoses and taping, is a more effective method than an anti-inflammatory therapy of NSAIDs and injections or an accommodative method of a heel cup. | 3 |
| Mehta  (2017) [75] | **Age**: 20-45  **Diagnosis**: plantar heel pain  **Inclusion**: pain in morning, pain after sitting a long time, pain increasing with extended walking or standing for more than 15 minutes, pain localized to area where the fascia attaches to the heel  **Exclusion**: recent surgery, pathology and fractures around ankle or foot, autoimmune or systemic or local inflammatory diseases, impaired circulation to lower extremity, neurological disorders or nerve entrapment, soft tissue abnormalities like fat pad syndrome, plantar fascia rupture, heel bruises, congenital deformity of ankle or foot and allergic to tape | N = 30 | **T_1_**: KT  (n=15)  **T_2_**: Mulligan taping and conventional physical therapy  (n=15) | **Pain**:  VAS  **Disability**:  Foot functional index  **Functional**:  n/a  **Time points**: 6 days intervention   1. Baseline 2. Post-treatment | **Pain**:  There was statistically significant difference  between Mulligan taping and KT on improvement of pain, however, Mulligan was a significantly large effect  **Disability**:  There was a statistically significant difference between Mulligan taping and KT on improvement of foot functional index for subjects with plantar fasciitis, however, Mulligan was a significantly large effect | Both groups were effective in improving the pain threshold in subjects with plantar heel pain.  Mulligan taping showed better results to alleviate pain and improve functional measures. | 4 |
| Radford (2006) [72] | **Age**: ≥ 18  **Diagnosis**: plantar heel pain  **Inclusion**: worst when first standing/walking after rest improve initially, but worsened with increasing activity, symptoms for > 4 weeks  **Exclusion**: inflammatory, osseous, metabolic or neurological abnormalities or had received a corticosteroid injection within the past three months, known tape allergies | N = 92 | **T_1_**: low-dye taping and sham ultrasound (n=46)  **T_2_**: sham ultrasound alone (n=46) | **Pain**:  VAS ‘first-step' pain  **Disability**:  Foot health status questionnaire  **Functional**:  n/a  **Time points**: 1 week intervention   1. Baseline 2. Post treatment | **Pain**:  No significant differences between groups for foot pain.  **Disability**:  No significant differences between groups for foot function and general foot health. | Low-dye taping provides a small improvement in 'first-step' pain compared to the sham intervention after one week. | 7 |
| Tsai  (2010) [74] | **Age**: not specified  **Diagnosis**: plantar fasciitis  **Inclusion**: onset within 10 months  **Exclusion**: history of foot surgery or any significant foot disorder such as arthritis, trauma, tumor | N = 52 | **T_1_**: traditional physical therapy (ultrasound, low frequency electrotherapy) (n=26)  **T_2_**: traditional physical therapy and KT (gastrocnemius and plantar fascia)  (n=26) | **Pain**:  McGill Pain Questionnaire  **Disability**:  Foot Function Index  **Functional**: Plantar fascia thickness  Structural change  **Time points**: 1 week intervention   1. Baseline 2. Post treatment | **Pain**:  Significantly greater pain decreases in experimental group  **Disability**:  There was significant improvement after treatment in the experimental group, but not in the control group  **Functional**:  Thickness of plantar fascia significantly reduced in KT group, compared to control, but the difference was not significant at the most inflamed site. No significant difference in the existence of hypoechoic phenomena [ultrasound assessment] between two groups | Treatment with standard therapy as well as KT for one week can provide pain relief in patients with plantar fascia compared to only a traditional physical therapy program. | 5 |
| Vishal  (2010) [73] | **Age**: 18-65  **Diagnosis**: plantar heel pain  **Inclusion**: pain > 1 month, pain located at heel or plantar surface of midfoot, consistent with plantar fasciitis  **Exclusion**: no previous surgery or treatment for plantar fasciitis in previous six months | N = 60 | **T_1_**: Calcaneal taping, ultrasound, passive stretching  (n=30)  **T_2_**: ultrasound, stretching, plantar fasciitis taping  (n=30) | **Pain**:  VAS  **Disability**:  Foot Function Index  **Functional**:  **Time points**: 7 days intervention   1. Baseline 2. Post treatment | **Pain**:  Significant changes in pain relief in both groups, but the calcaneal taping group showed greater improvements than the plantar fasciitis taping group  **Disability**:  Significant improvement in functional ability in both groups, but the calcaneal taping group showed greater improvements than the plantar fasciitis taping group | Calcaneal and plantar fasciitis taping may both be useful to reduce pain and improve function in patients with plantar fasciitis. | 4 |

**APPENDIX 2D: EVIDENCE TABLE FOR RANDOMIZED CONTROLLED TRIALS OF THE SHOULDER**

| Study | Population description | Sample size | Interventions | Outcomes/time points | Results | Key author conclusions | PEDro Score |
| --- | --- | --- | --- | --- | --- | --- | --- |
| Apeldoorn (2017) [96] | **Age**: 18-65  **Diagnosis**: subacromial pain syndrome  **Inclusion**: subacromial pain syndrome is primarily complaint, ≥ 2 positive tests (the painful arc of abduction test, the empty can test (Jobe test), the external rotation resistance test, and the Hawkins-Kennedy test)  **Exclusion**: structural narrowing of the subacromial space confirmed with radiography and/or diagnostic ultrasound, surgical history at the shoulder or cervical spine, polymyalgia rheumatica, rheumatoid arthritis, lupus erythematosus or fibromyalgia, severe arthritis of the glenohumeral joint, 3 or more subacromial corticosteroid injections in the last year, malignancy, severe trauma of the shoulder in the last 6 months, cerebral vascular accident, multiple sclerosis or Parkinson’s disease, type II diabetes, luxation or  fracture of the affected shoulder; cervico-radicular syndrome,  pathology of organs with negative consequences for the shoulder,  dementia; psychiatric disease, insufficient understanding of the Dutch language, bad condition of the skin around the affected shoulder because of a skin disease, allergy to tape | N = 140 | **T_1_**: Physical therapy  (n= 68)  **T_2_**: Rigid taping and physical therapy  (n=72) | **Pain**:  Numeric pain rating scale (NPRS)    **Disability**:  Simple shoulder test  Global perceived effect  **Functional**: n/a  **Time points**: 4 week intervention   1. Baseline 2. Post-treatment 3. 12 weeks 4. 26 weeks | **Pain**:  Significant difference between groups favoring the control group on pain intensity  There was a larger reduction in pain intensity at 4 weeks in the control group, but no difference at 12 and 26 weeks  **Disability**:  Significant difference between the groups was found favoring the intervention group on global perceived effect, but not on patient specific complaints during the 6-month follow-up  Period | Rigid tape technique used in this study did not bring additional value in a population of persons with subacromial impingement syndrome treated by individualized physical therapy and cannot be recommended for use in clinical practice. | 7 |
| Devereaux (2016) [99] | **Age**: 18 and older  **Diagnosis**: subacromial impingement syndrome  **Inclusion**: primary complaint of anterolateral shoulder pain, subacute onset of pain, a painful arc, a positive Hawkins– Kennedy test,  imaging consistent with impingement  **Exclusion**: history of shoulder surgery on the affected side, previous history of therapeutic kinesiology taping of the shoulder, medical contraindication to NSAIDs, frozen shoulder, labral tears, soft-tissue imaging documenting high-grade or partial-thickness rotator cuff tears, instability, glenohumeral arthritis, traumatic shoulder pathology, signs and symptoms because of referred pain (e.g. cervical), chronic pain, history of contact dermatitis | N = 100 | **T_1_**: KT and exercise  (n=33)  **T_2_**: NSAID and exercise  (n=29)  **C**: Exercise  (n=38) | **Pain**:  NPRS at rest and arm elevation  **Disability**:  Simple shoulder test  **Functional**:  Constant score  **Time points**: 2 weeks intervention   1. Baseline 2. Post-treatment | **Pain**:  All 3 treatment groups showed a statistically significant decrease in pain by all four measures when compared with pretreatment values  Between-group differences on pain were not statistically significant or clinically meaningful  **Disability**:  Between-group differences on pain were not statistically significant or clinically meaningful  **Functional**:  Between-group differences on pain were not statistically significant or clinically meaningful | There is no clinically meaningful difference between the use of Naprosyn 500 mg twice daily or KT as adjuvant therapies to an exercise program versus exercise  physiotherapy alone.  If adjuvant therapy is desired by either clinician or patient, KT seems to be better tolerated than Naprosyn 500 mg twice daily, although the difference is not statistically significant. | 5 |
| Djordjevic (2011) [100] | **Age**: 34-79  **Diagnosis**: rotator cuff lesion or impingement shoulder syndrome  **Inclusion**: shoulder pain, painful or restricted ROM with ADL  **Exclusion**: shoulder girdle fractures and dislocation, shoulder surgery in the last 12 months, adhesive capsulitis, full thickness rotator cuff tear, cervicobrachial pain due to cervical spine pathology, neuromuscular disorders in upper extremities, use of corticosteroid, use of non-steroidal anti-inflammatory therapy within 10 days before the first day of measuring ROM, Acute, clearly defined traumatic event that provoked pain and restricted shoulder motion | N = 20 | **T_1_**: KT and mobilization with movement (n=10)  **T_2_**: Supervised exercise  (n=10) | **Pain**: n/a  **Disability**: n/a  **Functional**: Pain-free active abduction and flexion  **Time points**: 10 day intervention   1. Baseline 2. Day 5 3. Day 10 | **Functional**:  Both groups showed improvement, but the KT with mobilization with movement group improved more quickly | Mobilization with movement and KT may be useful therapy modalities in improving active ROM in individuals with rotator cuff | 6 |
| Frassanito (2018) [101] | **Age**: 18 and older  **Diagnosis**: subacromial impingement with calcific tendinopathy  **Inclusion**: pain and shoulder ROM limitation in ADLs for at least 2 weeks, signs of rotator cuff calcific  tendinopathy on imaging (musculoskeletal ultrasound,  standard radiography, magnetic resonance), positive tests for functionality (Jobe, Lift-off, Patte, Palm-up, Yocum, Neer), absence of cognitive impairment  **Exclusion**: corticosteroids or corticosteroids/anesthetic injection, therapy for shoulder < 4 weeks prior to the study, ongoing cortisone or  NSAID therapy, partial or complete tear of tendons of the rotator cuff on imaging, severe glenohumeral and/or acromioclavicular osteoarthritis, surgery for direct shoulder injury, concomitant cervical symptoms consistent with radiculopathy, neurological diseases involving the shoulder, dermatological diseases, damaged skin (scars, infections,  or ulcerations not fully healed) involving the affected  shoulder, blood coagulation diseases, anticoagulant therapy, diabetes, tumors, bone infections, pregnancy, pacemaker, rheumatoid arthritis, connective tissue diseases, allergy to adhesive tape | N = 42 | **T_1_**: Extracorporeal shock wave therapy (ESWT)  (n=21)  **T_2_**: ESWT + KT  (n=21) | **Pain**:  VAS  **Disability**:  Disabilities of the Arm,Shoulder and Hand (DASH)  **Functional**: Subjective Shoulder Rating Questionnaire (SSRQ)  Oxford Shoulder Score (OSS)  **Time points**: 3 week intervention   1. Baseline 2. 1 week post-treatment 3. 4 weeks post-treatment 4. 12 weeks post-treatment | **Pain:**  Improvement was observed in the ESWT + KT group at all time points while ESWT improved significantly at 1 week follow-up.  **Disability:**  Improvement was observed in the ESWT + KT group compared to ESWT at all time points, and significantly at 1 week follow-up.  **Functional:**  Improvement in SSRQ and OSS was observed in the ESWT + KT group compared to ESWT at all time points, and significantly at 1 week follow-up. | KT is an effective adjuvant therapy for calcific tendinopathy of the shoulder in the short-term and enhanced the effects of ESWT in the medium to long-term.  There was little to no complications or side effects to report for KT. | 5 |
| Göksu  (2016) [102] | **Age**: 20-50  **Diagnosis**: subacromial impingement syndrome  **Inclusion**: pain for 1-3 months, pain before 150 degrees_ in any plane of range of motion, positive Jobe or Hawkins test, pain in ADLs, detection of rotator cuff tendinopathy or subacromial impingement syndrome on magnetic resonance imaging  **Exclusion**: previous fracture in the shoulder girdle complex, glenohumeral dislocation/subluxation, acromioclavicular sprain or separation, adhesive capsulitis, diabetes mellitus, use of anticoagulants, history of steroid injection therapy for shoulder, total rupture in the rotator cuff tendons on MRI, history of neck and shoulder surgery, or radicular neck pain within previous 3 months, patients taking regular systemic NSAIDs or steroids, pregnant or breastfeeding mothers and malignancy | N = 61 | **T_1_**: KT and home exercise  (n=30)  **T_2_**: Subacromial corticosteroid injection and home exercise  (n=31) | **Pain**:  VAS at rest and with movement  **Disability**:  Shoulder Pain and Disability Index  **Functional**: ROM: flexion and abduction  **Time points**: 9 days intervention   1. Baseline 2. 1 week post treatment 3. 4 weeks post treatment | **Pain**:  Both groups had significant improvements in all types of VAS at the end of first and fourth weeks after treatments  Injection group were found to be statistically higher  than in patients receiving KT with regard to shoulder pain at rest  **Disability**:  Both groups had significant improvements in Shoulder Pain and Disability Index at the end of first and fourth weeks after treatments  Injection group were found to be statistically higher than in patients receiving KT with regard to Shoulder Pain and Disability Index  **Functional**:  Both groups had significant improvements in all types of ROM at the end of first and fourth weeks after treatments  Injection group were found to be statistically higher  than in patients receiving KT with regard to abduction range of motion | Pain, shoulder ROM and function improved after both local injection therapy and KT.  However these improvements were more significant in injection group than in KT group at the end of first and four weeks. | 7 |
| Kaya, E  (2011) [103] | **Age**: 18-70  **Diagnosis**: shoulder impingement syndrome  **Inclusion**: positive empty can test, subjective complaint of difficulty performing ADLs, pain before 150 degrees active shoulder elevation, positive Hawkins–Kennedy test  **Exclusion**: intra-articular steroid injection, shoulder girdle fracture, glenohumeral dislocation,  acromioclavicular sprain, concomitant cervical symptoms consistent with radiculopathy, history of a shoulder surgery within the previous 12 weeks, or shoulder pain which lasted more than 6 months | N = 55 | **T_1_**: KT and home exercise program (isometric, ROM, strengthening, stretching) (n=30)  **T_2_**: passive modalities (ultrasound, TENS, exercise, hot pack) and home exercise program (n=25) | **Pain**:  Night Pain  Daily Pain  Pain with motion  **Disability**:  DASH  **Functional**: n/a  **Time points**: 2 weeks intervention   1. Baseline 2. 1 week 3. 2 week | **Pain**:  Rest, night, and movement median pain scores of KT were statistically significantly lower at the first week of the trial, but there was no significant difference at the end of the second week.  **Disability**:  Disabilities of the Arm Shoulder and Hand  score of KT group was significantly lower at the end of the second week than the physical therapy group. | KT has been found to be more effective than the local modalities at both first and second week of treatment.  KT may be an effective treatment for shoulder impingement syndrome. | 4 |
| Kaya, DO  (2014) [104] | **Age**: 30-60  **Diagnosis**: subacromial impingement syndrome  **Inclusion**: diagnosed with subacromial impingement syndrome by an orthopedic surgeon  **Exclusion**: cervical spine involvement, the presence of a glenohumeral joint adhesive capsulitis, or instability, a history of previous shoulder surgery, physiotherapy treatment of this disorder in the past 6 weeks;, or steroid injection into or around the shoulder in the past 2 months, recurrent complaints or long history of complaining over a year | N = 54 | **T_1_**: Manual treatment  (n=26)  **T_2_**: KT  (n=28) | **Pain**:  VAS at rest, with activity and at night  **Disability**:  DASH  **Functional**: n/a  **Time points**: 6 weeks intervention   1. Baseline 2. Post-treatment | **Pain**:  There were significant differences between pre and post, VAS in both groups, in that they both improved  Night pain in that the KT group had better results in comparison with the manual therapy group  **Disability**:  There were significant differences between pre and post DASH scores in both groups, in that they both improved | KT with exercise and manual therapy with exercise reduced pain and disability in  subacromial impingement in 6 weeks.  KT may have an adjunct effect of reducing night pain. | 7 |
| Kocyigit (2016) [105] | **Age**: 18-70  **Diagnosis**: subacromial impingement syndrome  **Inclusion**: ≥ 3 positive tests (Neer’s impingement sign, Hawkins’ test, painful arc test, and Jobe test), mild–moderate shoulder pain (VAS between 20 and 70 mm), no previous application of KTs  **Exclusion**: History of previous shoulder fracture/surgery in last 6 months, previous or concurrent diagnosis of rotator cuff tear, glenohumeral joint/ACJ osteoarthritis, cervical disc herniation accompanied by radicular symptoms, Inflammatory joint disease, history of electrotherapy or injection for the shoulder in the last  3 months | N = 41 | **T_1_**: KT  (n=21)  **T_2_**: Sham taping  (n=20) | **Pain**:  VAS with activity and at night  **Disability**:  NHP  **Functional**: Constant-Murley Score  ROM: abduction, flexion  **Time points**: 12 days intervention   1. Baseline 2. Post-treatment 3. 4 weeks | **Pain**:  Mean VAS for nocturnal pain was statistically significant between time points in the KT group  **Disability**:  KT group demonstrated significant change in  NHP pain and physical activity scores  **Functional**:  A significant main effect for both groups was observed regarding the mean change in Constant-Murley Score | Similar improvement in both groups raises the possibility that tape application might have been beneficial, regardless of the kind of the tape used.  We documented a significant decrease in VAS for nocturnal pain and Constant-Murley Score in both groups.  The KT group showed significant change in NHP pain and physical activity scores. | 7 |
| Kul  (2019) [106] | **Age**: 18-70  **Diagnosis**: subacromial impingement syndrome    **Inclusion**: clinically and radiologically diagnosed, supraspinatus tendinitis or partial supraspinatus rupture  **Exclusion**: physical therapy for shoulder area last 6 months, steroid injection to the shoulder < 3 months, chronic steroid use, adhesive capsulitis, presence of bicipital tendinitis, total rupture in the supraspinatus or other rotator cuff muscles, and rotator cuff tendinitis, history of shoulder joint surgery, systemic inflammatory disease, cervical radiculopathy, metabolic bone disease, diabetes mellitus | N = 40 | **T_1_**: KT + home exercise  (n=20)  **T_2_**: Modalities + home exercise  (n=20) | **Pain**:  VAS (rest, movement, night)  **Disability**:  American Shoulder and Elbow Surgeons Evaluation (ASESS-100)  Constant–Murley scale  Western Ontario Rotator Cuff Index (WORC)  **Functional**: ROM  **Time points**: 15 day intervention duration   1. Baseline 2. Post treatment 3. 1 month follow-up | **Pain**:  Both groups improved, however modalities was more effective than KT regarding  the end-of-treatment VAS. VAS improvement favored modality group at 1 month follow-up  **Disability**:  Improvement was observed in all variables in the KT and modality groups post-treatment, however, modalities was more effective than KT for AESS and WORC post-treatment. AESS favored modality group at 1 month follow-up.  **Functional**:  A significant improvement was observed in all variables in the KT and modality groups | Modalities are more effective during the early period.  KT may be a good supportive treatment especially during the early period.  End-of-the-study efficacy demonstrated that KT application provides  a considerable benefit in the treatment. | 4 |
| Kumar (2012) [97] | **Age**: Not specifically described  **Diagnosis**: subacromial impingement syndrome  **Inclusion**: unilateral shoulder pain >1 week localized (anterior and/or anterolateral) to the acromion and pain produced or increased during flexion and/or abduction of the symptomatic shoulder and at least any four of the following (Neer impingement sign, Hawkins sign, Pain reproduced during supraspinatus empty can test, Painful arc of movement between 60° to 120°), Pain with palpation on the greater tuberosity of the humerus  **Exclusion**: cervical radiculopathy or radiculitis, a rotator cuff tear, adhesive capsulitis, history of shoulder dislocation, subluxation or fracture, history of cervical, shoulder, or upper back surgery, shoulder instability, calcifying tendonitis, degenerative arthritis of the glenohumeral joint acromioclavicular joint | N = 52 | **T_1_**: Scapular (leukotape) taping  **T_2_**: Conventional treatment  ***sample size not described | **Pain**: n/a  **Disability**:  Shoulder Pain and Disability Index  **Functional**: Isometric muscle strength  **Time points**: 6 weeks intervention   1. Baseline 2. Post-treatment | **Disability**:  Taping group demonstrated improved score in Shoulder Pain and Disability Index  **Functional**:  Taping group demonstrated improvement in isometric muscle strength compared with the control group | Scapular taping may be a useful adjunct for promoting proper scapular position and can be used in conjunction with other interventions, like specifically selected exercises, patient education and about modification of performing overhead activities. | 4 |
| Miccinilli (2018) [107] | **Age**: 18-50  **Diagnosis**: rotator cuff tendinopathy    **Inclusion**: radiologic diagnosis of Rotator cuff tendinopathy (evidence of pathognomonic signs at musculoskeletal echography or magnetic resonance), 1 or more clinical shoulder test positive, provision of informed consent to participate, shoulder pain  **Exclusion**: total tendon lesion, previous fracture or shoulder dislocation, presence of skin lesions, coexisting elbow, forearm, wrist, hand and fingers pathologies, history of neoplasm, cognitive impairment, diabetes mellitus, statin use, diagnosis of anxiety-depressive syndrome | N = 30 | **T_1_**: KT + rehabilitation exercises  (n=21)  **T_2_**: sham KT + rehabilitation exercises  (n=19) | **Pain**:  NRS (at rest, activity)  **Disability**:  Constant-Murley scale  **Functional**: Medical Research Council (MRC) Shoulder strength assessment  **Time points**: 2 week intervention duration   1. Baseline 2. Post treatment | **Pain**:  No significant between group differences were observed  **Disability**:  No significant between group differences were observed  **Functional**:  MRC showed significant between group differences for MRC shoulder extension strength in favor of the KT group | KT combined with conventional rehabilitative treatment can facilitate immediate pain reduction during rehabilitative treatment, can increase function recovery, and increase strength recovery.  These findings are not strong enough to recommend the application of KT during rehabilitative treatment for rotator cuff tendinopathy. | 7 |
| Miller  (2009) [98] | **Age**: 18-70  **Diagnosis**: subacromial impingement syndrome  **Inclusion**: unilateral shoulder pain, duration > 6 weeks, symptoms reproduced with Hawkins-Kennedy test, effective written and verbal competence in the English language.  **Exclusion**: active neck movement reproducing pain in the shoulder region, presence of a glenohumeral joint adhesive capsulitis as identified by a loss of passive shoulder motion into external, history of previous shoulder surgery, physiotherapy treatment for this disorder in the past four weeks, steroid injection into or around the shoulder in the past two months, demonstrable neurological deficits, poor or fragile skin condition, report of past skin reaction associated with the use of adhesive tape | N = 17 | **T_1_**: scapular (rigid) taping and routine physical therapy  (n=6)  **T_2_**: routine physical therapy  (n=11) | **Pain**:  VAS  **Disability**:  Shoulder Pain and Disability Index  **Functional**: ROM: flexion and abduction  **Time points**: 6 weeks intervention   1. Baseline 2. 2 weeks 3. 6 weeks | **Pain**:  VAS during movements also much lower in taped group  **Disability**:  Shoulder Pain and Disability Index all markedly lower in the taped group than physiotherapy only.  **Functional**:  No differences in impairment measures  At 6 weeks, between group differences are minimal. | Scapular taping as an adjunct to physical therapy may be an effective short-term tool to manage shoulder impingement symptoms. | 6 |
| Pekyavas (2016) [108] | **Age**: Not specifically described  **Diagnosis**: subacromial impingement syndrome  **Inclusion**: symptoms > 3 months, complaints of shoulder pain (5/10 points from VAS)  **Exclusion**: soft tissue or bone problems affecting the shoulder, acute inflammation affecting the shoulder region, neurologic problems, scoliosis, systematic rheumatic problems, orthopedic problems or surgery affecting neck,  and obesity (BMI> 30 kg/m^2^). | N = 70 | **T_1_**: Exercise  (n=15)  **T_2_**: KT and exercise  (n=20)  **T_3_**: Manual therapy, KT and exercise  (n=16)  **T_4_**= Manual therapy, KT, high intensity laser, exercise  (n=19) | **Pain**:  VAS  **Disability**:  Shoulder Pain and Disability Index  **Functional**: ROM: flexion, abduction, external rotation  **Time points**: 15 days intervention   1. Baseline 2. Post-treatment | **Pain**:  VAS was not found to be significantly different in comparison with assessments before and after treatment  **Disability**:  In the KT and exercise group, Shoulder Pain and Disability Index total, and Shoulder Pain and Disability Index pain scale results were found significantly different  **Functional**:  ROM parameters were not found to be significantly different in comparison with assessments before and after treatment | Laser and manual therapy are more effective in decreasing pain and disability and increasing the ROM.  When the laser and manual therapy treatment approaches were compared, laser is found to be more effective on shoulder abduction ROM against manual therapy. | 6 |
| Shakeri (2013A) [109] | **Age**: Not specifically described    **Diagnosis**: subacromial impingement syndrome  **Inclusion**: positive ≥ 2 shoulder impingement screening items (history of proximal anterior or lateral shoulder pain > one week during the last six months, a painful arc sign during active shoulder elevation, tenderness to rotator  cuff tendon palpation, pain with resisted isometric shoulder abduction, positive Jobe’s test) and tested positive on ≥ 1 specific subacromial impingement tests (Neer sign, the Hawkins sign, Yocum test)  **Exclusion**: history of dislocation, fracture, or traumatic injuries within the shoulder complex, a history of shoulder surgery within the last 6 months, reproduction of symptoms during the cervical screening examination, failure to complete testing sessions, complete rupture of rotator cuff muscles, acute inflammation | N = 30 | **T_1_**: KT  (n=15)  **C**: Placebo  (n=15) | **Pain**:  VAS during movement and at night  **Disability**: n/a  **Functional**: Pain-free ROM: abduction, flexion scapular plane elevation  **Time points**: 7 days intervention   1. Baseline 2. Post tape application 3. 3 days with wearing tape 4. Post-treatment | **Pain**:  A significant difference of 3-4 points in VAS for nocturnal pain measures was also demonstrated after KT while control showed no significant difference in pain intensity during movement and nocturnal pain  **Functional**:  Significant differences in pain-free shoulder active  ROM in each of the three ROM measurements were  found after KT, while control showed no significant difference for pain free range of motion | KT produced an immediate improvement in pain intensity during movement and in the measure of nocturnal pain.  KT can be prescribed for patients with subacromial impingement syndrome especially when pain relief is the short-term goal of the treatment. | 6 |
| Shakeri (2013B) [110] | **Age**: Not specifically described  **Diagnosis**: subacromial impingement syndrome  **Inclusion**: positive sign in ≥2 shoulder impingement screening items (history of proximal anterior or lateral shoulder pain persisted for >1 week during the last six months, painful arc during active shoulder elevation, tenderness to palpation of rotator cuff tendons, pain with resisted isometric shoulder abduction, positive Jobe’s test), and ≥1 of the specific subacromial impingement tests (Neer sign, Hawkins sign, Yocum test)  **Exclusion**: history of dislocation, fracture or traumatic injuries on the tested shoulder complex, history of shoulder surgery within the last 6 months, reproduction of symptoms with active and passive ROM, or overpressure, failure to complete two testing sessions, cervical radiculopathy, complete rupture of rotator cuff muscles with acute inflammation | N = 30 | **T_1_**: KT  (n=15)  **C**: Placebo  (n=15) | **Pain**: n/a  **Disability**:  DASH  **Functional:** n/a  **Time points**: 7 days intervention   1. Baseline 2. Post-treatment | **Disability**: A significant decrease in DASH was observed in the KT group after 1 week in treatment compared with pre-treatment score  However, the change in DASH score was significant in control group  Change in DASH after 1 week was significantly greater for treatment group than control group | The application of taping produces improvement in disability of the shoulder, arm and hand and can be prescribed for patients with shoulder impingement syndrome. | 6 |
| Şimşek (2013) [111] | **Age**: 18-70  **Diagnosis**: subacromial impingement syndrome  **Inclusion**: pain interferes with daily routine, >1 month duration, positive Neer’s test and positive Hawkin's impingement  **Exclusion**: calcific tendinitis and degenerative arthritis in plain roentgenograms, pathological findings in addition to subacromial effusion in MR images, a history of shoulder, waist and chest surgery, fracture or dislocation of the affected shoulder, cervical problems accompanied by radicular symptoms, inflammatory joint disease, and physiotherapy for the shoulder within last three months | N = 38 | **T_1_**: KT and exercises  (n=19)  **T_2_**: sham KT and exercises  (n=19) | **Pain**:  VAS: rest, activity, night  **Disability**:  DASH  **Functional**: Painless ROM: flexion, abduction, internal rotation, external rotation  **Time points**: 12 days intervention   1. Baseline 2. Day 5 3. Post-treatment | **Pain**:  Therapeutic group significant differences observed at day 12 specifically for night/activity pain scores  **Disability**:  Therapeutic group significant differences observed at day 12  **Functional**:  Therapeutic group significant differences day 12 in painless abduction ROM, and muscle strength during external rotation | The addition of KT to an exercise program appears to be more effective than exercise alone to treat subacromial impingement syndrome . | 5 |
| Subaşı (2016) [112] | **Age**: Not specifically described  **Diagnosis**: subacromial impingement syndrome  **Inclusion**: pain >1 month, one or more positive results for shoulder impingement tests (Neer’s, Hawkins, painful arc, supraspinatus and 0° abduction) or supraspinatus lesion revealed by radiological examination  **Exclusion**: Not specifically described | N = 70 | **T_1_**: Injection (betamethasone)  (n=35)  **T_2_**: KT  (n=35) | **Pain**:  VAS for movement pain  **Disability**:  Shoulder Pain and Disability Index  **Functional**: ROM in all planes  **Time points**: 3 weeks intervention   1. Baseline 2. 1 month 3. 3 months | **Pain**:  Significant differences were detected in VAS in both groups  **Disability**:  Significant differences were detected in Shoulder Pain and Disability Index in both groups  **Functional**:  Significant differences were detected in all planes ROM in both groups  Statistically significant difference was determined in the injection group in improved active extension degree | Both KT and steroid injection in conjunction with an exercise program can be beneficial in the rehabilitation of subacromial impingement syndrome.  KT can be considered a safe and inexpensive alternative for subacromial impingement syndrome especially for patients who have anxiety problems about injections or for whom steroid injections are contraindicated. | 5 |
| Teys  (2013) [114] | **Age**: 18 and older  **Diagnosis**: shoulder pain  **Inclusion**: pain in antero-superior aspect of one shoulder, pain > 4 weeks duration, reduced shoulder elevation due to pain, positive response to application of shoulder Mulligan's Mobilization with Movement (MWM) at initial visit  **Exclusion**:history of cancer, previous fractures of the shoulder complex, recent shoulder surgery or corticosteroid injection, any neurological or autoimmune disorder or any recent shoulder dislocation, allergy to adhesive tape, pain was exacerbated on neck examination | N = 25 | **T_1_**: MWM  (n=13 )  **T_2_**: MWM with tape  (n=12)  **Crossover study with one week washout period between treatments | **Pain**:  Pressure pain threshold  VAS  **Disability**: n/a  **Functional**: Range of motion  **Time points**:30 minute intervention   1. Baseline 2. Post-treatment 3. 24 hours 4. 7 days | **Pain**:  No significant PPT differences between groups for any time point  Pain severity was significantly different over time but not between groups  **Functional**:  MWM with tape provided statistically and clinically significant improvement of 20 degrees ROM maintained for one week, whereas MWM alone only produced improvement for 30 minutes post intervention | In individuals who show positive responses to MWM, a single intervention of MWM with tape can provide an improvement in ROM for up to one week, compared to MWM alone. | 6 |
| Thelen (2008) [113] | **Age**: 18-50  **Diagnosis**: subacromial impingement  **Inclusion**: shoulder pain < 150 degrees elevation, positive empty can test, positive Hawkins-Kennedy test, difficulty performing ADLs  **Exclusion**: shoulder girdle fracture, glenohumeral dislocation, acromioclavicular sprain, concomitant cervical spine symptoms, a history of shoulder surgery within the previous 12 weeks, or shoulder pain > 6 months | N = 42 | **T_1_**: KT  (n=21)  **T_2_**: Sham KT (n=21) | **Pain**:  VAS  **Disability**:  Shoulder Pain and Disability Index  **Functional**: ROM  **Time points**: 6 days intervention   1. Baseline 2. 3 days 3. Post-treatment | **Pain**:  No difference between groups were present  **Disability**:  No difference between groups were present  **Functional**:  KT provided statistically significant improvements in pain-free shoulder abduction immediately after application | KT tape may help give immediate improvement in pain-free shoulder abduction, but over time no significant differences in pain or function between groups. | 9 |

**APPENDIX 2E: EVIDENCE TABLE FOR RANDOMIZED CONTROLLED TRIALS OF THE ELBOW**

| Study | Population description | Sample size | Interventions | Outcomes/time points | Results | Key author conclusions | PEDro Score |
| --- | --- | --- | --- | --- | --- | --- | --- |
| Bhambhani (2016) [115] | **Age**: 20-60  **Diagnosis**: Tennis elbow  **Inclusion**: no deformity of the affected wrist or elbow  **Exclusion**: clinical disorder which may become worse with taping, such as skin disease, dermatitis, eczema. | N = 24 | **T_1_**: Taping and Conventional therapy  (n=12)  **T_2_**: Conventional therapy  (cryotherapy, stretching & strengthening exercise, deep transverse friction massage, and ultrasound)  (n=12) | **Pain**:  VAS: rest, forceful wrist extension, strong grasp, cozen test, resisted middle finger extension, palpation  **Disability**: n/a  **Functional**: n/a  **Time points**: 6 treatment in 3 weeks duration   1. Baseline 2. Intervention 3. Post-treatment | **Pain**:  Taping with conventional therapy is more effective than conventional therapy alone | Conventional physiotherapy with taping is better treatment than the conventional physiotherapy alone for reducing pain and disability in tennis elbow patient.  ***Only measure VAS, so can not technically speak to disability despite statement | 3 |
| Desai  (2014) [116] | **Age**: 30-50  **Diagnosis**: Lateral epicondylitis  **Inclusion**: Pain at lateral epicondyle, onset >6 months, increased pain with gripping, palpation, resisted finger and wrist extension, positive Mill's and Cozen's test  **Exclusion**: Concomitant bony, neurological impairments, neurological diseases, previous trauma to the elbow region, previous surgery to the elbow region, cervical radiculopathy, systemic diseases, allergic to tape | N = 40 | **T_1_**: Taping (rigid) and exercise  (n=20)  **T_2_**: Exercise  (n=20) | **Pain**:  VAS  **Disability**:  Patient-rated tennis elbow evaluation questionnaire  **Functional**: n/a  **Time points**: 4 week intervention   1. Baseline 2. Post-treatment | **Pain**:  Statistically significantly better VAS in group with taping and exercise versus exercise alone  **Disability**:  Statistically significantly better patient-rated tennis elbow evaluation questionnaire in group with taping and exercise versus exercise alone | Taping with exercise program is more effective than just exercise program in reduction of pain and improvement of function. | 5 |
| Eraslan (2017) [118] | **Age**: 36-66  **Diagnosis**: Lateral epicondylitis  **Inclusion**: >3 months duration, diagnosis as a result of occupational exposure to forceful and repetitive hand activities.  **Exclusion**: inflammatory, autoimmune, endocrine, or renal diseases, cubital tunnel syndrome, carpal tunnel syndrome; cervical radiculopathy; additional shoulder–hand–wrist pathologies, arthritis; upper extremity operations or traumas, allergies to adhesive tape and those who received corticosteroid injections due to lateral epicondylitis within the last 3 months were excluded | N = 45 | **T_1_**: Physiotherapy  (n=15)  **T_2_**: Physiotherapy and KT  (n=15)  **T_3_**: Physiotherapy and extracorpeal shockwave therapy  (n=15) | **Pain**:  VAS  **Disability**:  Patient-Rated Tennis Elbow Evaluation  **Functional**: Cyriax resisted muscle test  Maximum grip strength  **Time points**: 3 week intervention   1. Baseline 2. Post-treatment | **Pain**:  KT group yielded better results in decreasing pain intensity  **Disability**:  KT group yielded better results in improving Patient-Rated Tennis Elbow Evaluation  **Functional:**  KT group yielded better results in improving Cyriax resisted muscle test and maximum grip strength | Physiotherapy alone or coupled with KT or extracorpeal shockwave decreases pain associated with lateral epicondylitis and improves patients’ functional scores.  KT along with physiotherapy yielded better results after short-term management in decreasing pain during activity. | 5 |
| Giray  (2019) [119] | **Age**: not specifically described  **Diagnosis**: lateral epicondylitis    **Inclusion**: duration < 12 weeks, tenderness and pain over lateral elbow, provocation of the lateral elbow pain with at least one of the following tests – resisted middle finger extension (Maudley’s test), resisted wrist extension or passive  stretch of wrist extensors (Mill’s test), diagnosis confirmed by ultrasound  **Exclusion**: cervical spondylosis or radiculopathy, diabetes mellitus, concomitant neuropathy, entrapment neuropathy, polyneuropathy, systemic arthritic conditions, pregnancy, history of surgery or acute trauma in the elbow, history of injection or physiotherapy for epicondylitis, allergy to tape | N = 30 | **T_1_**: KT + exercise  (n=10)  **T_2_**: Sham tape + exercise  (n=10)  **C**: Exercise  (n=10) | **Pain**:  VAS (rest, daily activity, night)  **Disability**:  Patient-rated tennis elbow evaluation (PRTEE)  QuickDASH  **Functional**: Grip strength  **Time points**: 2 week intervention   1. Baseline 2. Post-treatment 3. 4 weeks | **Pain**:  KT had a post-treatment decrease in VAS at rest and with daily activity compared to sham taping.  **Disability**:  PRTEE at post-treatment and at 4 weeks were statistically significantly lower in KT group compared to sham taping and control group.  **Functional**:  KT had immediate effect on grip strength compared to sham taping. | KT in addition to exercises is more effective than sham taping and exercises only in improving pain in daily activities and arm disability due to lateral epicondylitis.. | 7 |
| Kachanathu (2013) [117] | **Age**: 20-40  **Diagnosis**: Lateral epicondylitis  **Inclusion**: 3 weeks from onset of symptoms, discomfort/tenderness lateral epicondyle, pain with 2 of 3 tests : (resisted middle finger extension, resisted wrist extension, passive wrist extensors stretch)  **Exclusion**:bilateral elbow pain, history of surgery to elbow or distal upper extremity, combined lesions (e.g. Cervical and elbow problems, multiple lesions about elbow, Carpal tunnel syndrome), any medications or injection to elbow in the past, any other medical or neurological condition, any patient beyond 6 weeks, allergies to adhesive tape | N = 45 | **T_1_**: Forearm band (nonelastic support)  (n=15)  **T_2_**: Elbow (rigid) taping (n=15)  **C**: conventional physiotherapy  (n=15) | **Pain**:  VAS - grip strength  **Disability**:  Patient-rated forearm evaluation questionnaire  **Functional**:  Grip strength  **Time points**: 4 week intervention   1. Baseline 2. 2 weeks 3. Post-treatment | **Pain**:  Significant changes in the pain-free grip strength  in all the three groups  **Disability**:  There was a significant diminution in the level of pain in the affected arm in all three groups. The findings  indicated highly significant differences for  Group-A.  **Functional**:  Highly significant post-test patient-rated forearm evaluation questionnaire, Group 1 > Group 2 > Group 3 | Forearm band produces significantly greater responses in both patient-rated forearm evaluation questionnaire  and grip strength compared to taping and conventional physiotherapy. | 5 |
| Shakeri (2018) [120] | **Age**: not specifically described  **Diagnosis**: lateral epicondylitis    **Inclusion**: female, pain was elicited by all 3 clinical pain provocation tests (Cozen’s, Mill’s, and third finger extension)  **Exclusion**: proximal upper extremity  or neck symptoms, history of cervical pathology, nerve entrapment syndromes, nonunion fractures, surgical treatments for lateral epicondylitis, steroid injection for elbow pain < 6 months | N = 30 | **T_1_**: KT  (n=15)  **T_2_**: sham KT (without tension)  (n=15) | **Pain**:  VAS  PPT  **Disability**:  DASH  **Functional**: Grip strength  **Time points**: 1 week intervention duration   1. Baseline 2. Post treatment 3. 2 days post-treatment | **Pain**:  No significant difference in immediately after intervention for either group  Pain during daily activities was more effective in group with KT than no tension  **Disability**:  No significant difference in immediately after intervention for either group  DASH was more effective in group with KT than no tension  **Functional**:  No significant difference in immediately after intervention for either group | The application of KT produces an improvement in pain  intensity and upper extremity disability in subjects with lateral epicondylitis and KT with tension was more effective  than sham KT group. | 5 |
| Wegener (2016) [121] | **Age**: 18-80  **Diagnosis**: lateral elbow tendinosis  **Inclusion**: referral from their  general practitioner or orthopaedic or hand surgeon, medical investigations confirming lateral elbow tendinosis (e.g. MRI)  **Exclusion**: any comorbidities  (such as fractures, pain syndromes or inflammatory diseases), patients with any contraindications to the use of taping (such as skin allergies to the tape material), patients who were unable to understand written material in English and those unable to give informed consent | N = 40 | **T_1_**: Intervention (elastic therapeutic tape with structured exercise program)  (n=14)  **T_2_**: Sham (tape was applied with no tension with structured exercise program)  (n=13)  **C**: Control (no taping and provided with structured exercise program)(n=13) | **Pain**: n/a  **Disability**:  Patient-Rated Tennis  Elbow Evaluation  Short Form 36  Occupational Self Assessment  **Functional**: Pain-free grip strength  **Time points**: intervention duration   1. Baseline 2. 3 months 3. 6 months | **Disability**:  Patient-Rated Tennis Elbow Evaluation scores were of greater magnitude in the intervention group, there were no statistically significant difference between the three groups at three or six months.  Improvements were also seen in short form 36 and occupational self-assessment though not significant  **Functional**:  Improvements were also seen in grip strength though not significant | All groups improved on key outcomes.  It is likely that eccentric exercises and activity modification techniques are more appropriate, evidence-based, and cost-effective in the clinical management of lateral elbow tendinosis. | 7 |

**APPENDIX 2F: EVIDENCE TABLE FOR RANDOMIZED CONTROLLED TRIALS OF THE HAND AND WRIST**

| Study | Population description | Sample size | Interventions | Outcomes/time points | Results | Key author conclusions | PEDro Score |
| --- | --- | --- | --- | --- | --- | --- | --- |
| Aktürk (2018) [122] | **Age**: 20-65  **Diagnosis**: carpal tunnel syndrome    **Inclusion**: mild to moderate carpal tunnel syndrome on electroneuromyography, pain or numbness spreading to the palmar face of the hand, one of the following positive: Tinel, Phalen’s, or carpal compression tests in the physical examination, symptoms duration >3 months  **Exclusion**: diabetes, rheuma-  toid arthritis, thyroid disease, brachial plexopathy, polyneuropathy, cervical radiculopathy, wrist fractures, prior carpal tunnel syndrome surgery, steroid injections | N = 58 hands  44 patients | **T_1_**: KT + exercise  (n=28 hands)  **T_2_**: Splinting + exercise  (n=30 hands) | **Pain**: n/a  **Disability**:  Boston Carpal Tunnel Syndrome Questionnaire (BCTSQ)  **Functional**:  Nerve conduction studies  Tinel  Phalen’s signs  Compression test  Sensory examination  Motor assessment of hand  **Time points**: 5 week intervention duration   1. Baseline 2. 6 weeks post-treatment | **Disability**:  There were statistically significant differences in BCTSQ in favor of KT group  **Functional**:  There were statistically significant differences in motor distal latency, sensory latency, sensory conduction velocity, provocative test responses in favor of KT group | KT has positive effects on electrophysiological  changes and physical examination findings and may also help prevent further disease progression in carpal tunnel syndrome when applied timely and with the appropriate technique. | 5 |
| Geler Kulcu (2016) [123] | **Age**: 18 or older  **Diagnosis**: Carpal tunnel syndrome  **Inclusion**: symptoms <1 year, Mild and moderate CTS patients, according to nerve conduction studies. Mild abnormal median nerve peak sensory conduction velocity (<42 m/s) and normal median nerve motor latency (<4 ms). Moderate defined as NCS abnormalities for median nerve peak sensory conduction velocity (<42 m/s) and abnormal median nerve motor distal latency (>4 ms)  **Exclusion**: any secondary entrapment neuropathy (e.g., diabetes, inflammatory arthritis, hypothyroidism, previous wrist trauma), pregnancy, skin infection on the forearm, cervical radiculopathy, polyneuropathy, previous history of carpal tunnel decompression surgery, and corticosteroid injection into the carpal tunnel | N = 45  (60 total limbs) | **T_1_**: KT  (n= 13)  (20 wrists)  **T_2_**: KT Placebo  (n= 13)  (20 wrists)  **T_3_**: Splint  (n= 14)  (20 wrists) | **Pain**:  VAS  Douleur Neuropathique 4 Questionnaire  **Disability**:  Boston questionnaire  **Functional**: Grip strength  **Time points**: 4 week intervention   1. Baseline 2. Post-treatment | **Pain**:  VAS decreased in all groups and there was no difference among the groups regarding VAS scores after treatment  Douleur Neuropathique 4 scores significantly decreased in all groups and there was no difference among groups regarding DN4  scores after treatment  **Disability**:  There was significant difference between KT and splint groups, which was in favor of KT  **Functional**:  Grip strength improved for splint but not in KT or KT placebo. There was no significant difference among the groups regarding grip strength after treatment | There was improvement in grip strength observed only by  splinting, and improvement in functional status was observed only by KT.  KT application for the treatment of CTS is as useful as applying an orthotic device regarding pain relief and superior to orthotic device in functional status improvement. The KT should be used as an alternative treatment method for CTS without the disadvantage of restricting daily activities. | 7 |
| Güner  (2018) [124] | **Age**: 18-65  **Diagnosis**: carpal tunnel syndrome    **Inclusion**: mild to moderate CTS diagnosis according to electroneuromyography findings  **Exclusion**: predisposing etiological factors for polyneuropathy, diabetes mellitus, acute trauma, rheumatologic diseases, chronic renal failure, pregnancy, hypothyroidism,  hyperthyroidism, continuous NSAID use in the last 1 month, physical therapy in the last 1 month, local steroid injections in carpal tunnel area < 3 months, severe CTS, h/o malignancy, cervical radiculopathy, ulnar neuropathy | N = 64 | **T_1_**: low power laser  (n=21)  **T_1_**: KT + low power laser  (n=22)  **C**: sham laser  (n=21) | **Pain**:  Visual Numeric Pain Score  **Disability**:  Boston Carpal Tunnel Syndrome Questionnaire (BCTSQ)  **Functional**: Hand grip strength  Finger pinch test  Electroneuromyography (ENMG)  **Time points**: 3 week intervention duration   1. Baseline 2. Post-treatment 3. 12 weeks | **Pain:**  Laser and KT + laser improved and were not found to be significantly different in the short- or long-term  **Disability:**  Laser and KT + laser improved and were not found to be significantly different in the short- or long-term  **Functional:**  Laser and KT + laser improved and were similar in the short-term, but there was a statistically significant difference in favor of KT + laser in finger pinch test and hand grip strength in the long-term | Laser treatment provided improvement in pain, and functional status both alone and combined with KT.  Combination of laser with KT did significantly increase HGS and FPS in the long-term.  KT and laser method in the treatment of CTS may be an effective and reliable treatment option in clinical parameters. | 8 |
| Homayouni (2013) [126] | **Age**: 18-65  **Diagnosis**: de Quervain’s Disease  **Inclusion**: >4 weeks duration, pain, swelling, tenderness over the first extensor compartment, positive finklestein test  **Exclusion**: pregnancy,  history of hand or wrist fracture or acute trauma, dislocation or surgery, rheumatoid arthritis, wrist injection in the three last months | N = 60 | **T_1_**: KT  (n=30)  **T_2_**: Physical therapy modalities  (Paraffin bath, ultrasound, TENs, friction massage)  (n=30) | **Pain**:  VAS  **Disability**: n/a  **Functional**: Swelling by inspection and palpation  **Time points**: 4 week intervention   1. Baseline 2. Post-treatment | **Pain**:  VAS differences were significant between the KT and PT groups statistically  **Functional**:  Swelling improvement was significant in the KT group but not in the PT group | Patients respond more favorably to KT rather than PT | 5 |
| Kim  (2019) [127] | **Age**: not specifically described  **Diagnosis**: dorsal wrist pain  **Inclusion**: < 50 PROM in wrist extension, >2 months of nonspecific dorsal wrist pain due to joint hypomobility without ligamentous instability  **Exclusion**: history of surgery,  fracture, or tenosynovitis (intersection syndrome) around the wrist, dorsal wrist ganglion, scapholunate ligament injury, neurological symptoms such as carpal tunnel syndrome, skin sensitivity to taping | N = 30 | **T_1_**: carpal stabilizing rigid tape  (n=15)  **T_2_**:KT  (n=15) | **Pain**:  VAS  **Disability**: n/a  **Functional**: ROM (Wrist extension)  **Time points**: 1 week intervention duration   1. Baseline 2. Post treatment | **Pain**:  Both groups improved, however, VAS score was significantly higher in the carpal stabilizing group than the KT group  **Functional**:  AROM and PROM of wrist extension significantly increased in both groups  When comparing pre- and post-intervention values, the changes in AROM and PROM were significantly greater in the carpal stabilizing tape group than in the KT group. | Rigid tape can be used to increase wrist ROM and decrease wrist pain in patients with dorsal wrist pain during weight-bearing hand activities. | 6 |
| Wade  (2018) [128] | **Age**: 18 and older  **Diagnosis**: proximal interphalangeal joint osteoarthritis    **Inclusion**: osteoarthritis of the proximal interphalangeal joint of any finger based on both symptoms and  radiographic changes  **Exclusion**: non-English speakers, unable to consent, lacked the dexterity to cut and apply the tape to the painful finger, active infection, unhealed wound on the same hand, dermatological conditions involving the proposed trial finger, vulnerable or thin dorsal skin | N = 10 | **T_1_**: Elastic suture tape (analgesic potential supportive configuration)  (n=5)  **C**: Control elastic suture tape (proposed to lack analgesic effect)  (n=5) | **Pain**:  VAS  **Disability**:  QuickDASH  **Functional**: Active ROM  **Time points**: 3 week intervention duration   1. Baseline 2. 1st week 3. 2nd week 4. 3rd week | **Pain**:  No significant group-difference in the change in pain at all time points  For the whole sample, the application of KT reduced reported pain by 6%, although there was no between-group difference  **Disability**:  No significant changes in either group at all time points reported  **Functional**:  Taping did not affect the ROM in any finger joint in either group | Supportive tape on the dorsum of proximal interphalangeal joints  affected by OA may reduce perceived pain.  Whether pain reduction is due to KT or not requires  further investigation. | 9 |
| Yıldırım (2018) [125] | **Age**: 18-60  **Diagnosis**: carpal tunnel syndrome    **Inclusion**: electrophysiologically diagnosed with mild or moderate  CTS, symptoms ≥3 months, conservative treatment recommended  **Exclusion**: electrophysiological diagnosis of severe CTS, thenar  atrophy, local corticosteroid injection or physical therapy for CTS within the past 3 months, cervical disc herniation, peripheral nerve damage,  Upper extremity entrapment neuropathy, pregnancy | N = 38 wrists from 21 patients | **T_1_**: KT + exercise  (n=19)  **C**: Exercise (tendon and nerve gliding)  (n=19) | **Pain**: n/a  **Disability**:  BCTSQ  **Functional**: Moberg pick-up test  Hand grip  Pinch strength  **Time points**: 6 week intervention duration   1. Baseline 2. 3 weeks 3. 6 weeks | **Disability**:  There was a significant improvement in BCTQ at 3 weeks in the intervention group compared to the control group  **Functional**:  There was a significant improvement in  the severity of symptoms and functional capacity scale as  assessed by the Moberg pick-up test at 3 weeks  in the intervention group compared to the control group | Tendon and nerve gliding exercises may be effective in the short-term clinical outcomes of patients  with the diagnosis of mild or moderate CTS.  The addition  of KT to exercises provides an additional, but  limited, contribution. | 7 |

**APPENDIX 2G: EVIDENCE TABLE FOR RANDOMIZED CONTROLLED TRIALS OF THE LUMBAR SPINE**

| Study | Population description | Sample size | Interventions | Outcomes/time points | Results | Key author conclusions | PEDro Score |
| --- | --- | --- | --- | --- | --- | --- | --- |
| Added (2016) [142] | **Age**: 18-60  **Diagnosis**: chronic non-specific low back pain  **Inclusion**: pain >3 months seeking physical therapy  **Exclusion**: pregnant or had contraindications to physical exercise according to the American College of Sports Medicine, no serious spinal pathology, nerve root compromise, contraindication to the use of KT due to allergy and intolerance to the tape or cardiorespiratory disease | N = 148 | **T_1_**: physical therapy (pragmatic application of exercise and manual therapy)  (n=74)  **T_2_**: physical therapy plus KT  (n=74) | **Pain**:  NPRS  **Disability**:  RMDQ  **Secondary outcomes**  Global Perceived Effect scale  MedRisk Instrument for Measuring Patient Satisfaction with Physical Therapy Care  **Time Points**: 5 week intervention   1. Baseline 2. Post-treatment 3. 3 months 4. 6 months | **Pain**:  Within-group analysis found treatments reduced pain. No between-group differences in NPRS were observed at post-treatment, 3 months, or 6 months  **Disability:**  Within-group analysis found treatments reduced disability. Between group differences RMDQ favored physical therapy at 6 months  **Secondary Outcome**:  Within-group analysis found treatments increased perceived improvement. no significant between-group differences at any time point for satisfaction or perceived improvement | No additive effect of using KT in addition to pragmatic physical therapy for improvement in pain rating or disability was observed. | 8 |
| Al-Shareef  (2016) [143] | **Age**: 25-55  **Diagnosis**: chronic non-specific low back pain  **Inclusion**: low back pain ≥ 3 months duration  **Exclusion**: systemic metabolic and/or neurological disorder; neuropathic pain; spinal surgery and/or fracture; pregnancy; previous physical therapy treatment during the last 6 weeks; and contraindicated to KT (e.g., skin allergy and/or intolerance to tape, dermatitis, or preexisting skin lesion and infection) | N = 44 | **T_1_**: KT (10-15% tension)  (n=21)  **C**: placebo tape (0% tension)  (n=23) | **Pain**:  VAS  **Disability**:  ODI (Arabic)  **Secondary Outcome**: Trunk flexion ROM modified Schober’s Test  **Time points**: 2 week intervention   1. Baseline 2. 2 week post-intervention 3. 4 week follow-up | **Pain**:  Both the experimental and control groups changed significantly, however, pain intensity improvement was greater in the KT group and was maintained after 4-week follow-up  **Disability**:  ODI improved significantly in both groups, though greater in the KT group and was sustained at 4-week follow-up  **Secondary Outcome**:  Trunk flexion ROM significantly improved in both groups was greater in the KT group and was maintained at 4-week follow-up. | KT appears to reduce pain and disability and improves trunk flexion ROM after 2 weeks of application and sustained for 4 weeks.  The effects of KT were very small to be considered clinically relevant when compared with placebo taping for pain, disability, and ROM. | 7 |
| Araujo  (2018) [144] | **Age**: 18-80  **Diagnosis**: low back pain  **Inclusion**: pain ≥ 3 months  **Exclusion**: any contraindication to physical exercise, serious spinal pathology (i.e. nerve root compromise, fracture, tumor), serious cardiopulmonary conditions, pregnancy or any contraindications to the use of taping were excluded | N = 148 | **T_1_**: KT with skin convolutions  (n=74)  **C**: Sham KT (taping without skin convolutions)  (n=74) | **Pain**:  NPRS  **Disability**:  RMDQ  Global Perceived Effect scale  **Functional**: none  **Time points**: 4 week intervention   1. Baseline 2. 6 month follow-up | **Pain**:  Both groups experienced a reduction in pain at 6 months but there were no significant between-group differences  **Disability**:  Both groups experienced a reduction in RMDQ at 6 months but there were no significant between-group differences | There was no effect of KT versus sham on pain intensity, global impression of recovery or disability. Improvements over time in both groups can be attributed to the natural history of the condition, regression to the mean and/or non-specific effects of treatment. | 8 |
| Bae  (2013) [145] | **Age**: not specifically described  **Diagnosis**: chronic low back pain  **Inclusion**: pain > 12 weeks, VAS and ODI scores were 6 or higher, those who had not conducted exercises using the muscles of the lumbar spinal area for the past three months  **Exclusion**: lumbar surgery, structural malformation or other musculoskeletal disease, skin sensitivity to tape, previous tape treatment experience, active prescription for adrenocortical hormone or pain alleviation medication | N = 20 | **T_1_**: placebo tape and physical therapy (hot pack, ultrasound, TENS)  (n=10)  **T_2_**: KT and physical therapy  (n=10) | **Pain**:  VAS  **Disability**:  ODI  **Functional**: none  **Time points**: 12 week intervention   1. Baseline 2. 12 weeks | **Pain**:  VAS significantly decreased in both groups.  **Disability**:  ODI significantly decreased in both groups and the most significant changes were observed in the ODI of the experimental group | Results suggest that application of physical therapy and KT reduces pain and affects functional performance capabilities. In particular, the experimental group’s VAS significantly decreased. | 5 |
| Castro-Sanchez  (2012) [146] | **Age**: 18-65  **Diagnosis**: low back pain  **Inclusion**: pain > 3 months, RMDQ > 4, fail to achieve flexion-relaxation in lumbar muscles during trunk flexion  **Exclusion**: radiculopathy, lumbar stenosis, fibromyalgia, spondylolisthesis, previous spinal surgery or KT, corticosteroid treatment in previous two weeks, and central or peripheral nervous system disease | N = 60 | **T_1_**: KT  (n=30)  **T_2_**: placebo KT  (n=30) | **Pain**:  VAS  **Disability**:  ODI  RMDQ  Tampa scale for kinesiophobia (TSK)  **Functional**:  Trunk flexion ROM  **Time points**: 1 week intervention   1. Baseline 2. Post treatment 3. 4 week follow-up | **Pain**:  Pain improved significantly at one week and was maintained at 4 weeks  **Disability**:  After one week, statistically significant improvement in disability, but not significant at 4 weeks. Fear of movement did not show statistically significant differences, nor did trunk flexion ROM.  **Functional**: trunk flexion ROM did not show statistically significant differences | Immediate improvements after application in disability, pain, endurance of trunk muscles, and perhaps trunk flexion ROM were statistically significant, however, small effects disappeared at week 4, except for pain and trunk muscle endurance. | 9 |
| Chen  (2012) [147] | **Age**: 18-65  **Diagnosis**: non-specific low back pain  **Inclusion**: pain > 6 weeks or recurrent low back pain, discomfort during trunk flexion  **Exclusion**: spinal pathology, major trauma, systemic disease, cancer, osteoporosis, inflammatory disease, neurological deficit, pregnancy, previous back surgery or waiting for surgery, active or pending legal proceedings due to low back pain, skin sensitivity to tape, dermatitis or a pre-existing skin lesion over the taping area | N = 43 | **T_1_**: functional fascial taping and standardized simple trunk flexion exercise  (n=21)  **T_2_**: placebo taping and standardized simple trunk flexion exercise (n=22) | **Pain**:  VAS  **Disability**:  ODI  **Functional**: none  **Time points**: 2 week intervention   1. Baseline 2. Post treatment 3. 6 week follow-up 4. 12 week follow-up | **Pain**:  The minimum clinically important difference was similar between groups. However, higher proportion of patients in the taping group reported lower scores for worst pain on VAS.  **Disability**:  The minimum clinically important difference was similar between groups | Functional fascial taping reduced worst pain in patients with non-specific low back pain during treatment phase. No medium differences in pain or function were found. | 9 |
| Elshinnawy (2019) [148] | **Age**: 30-50  **Diagnosis**: chronic low back dysfunction    **Inclusion**: chronic low back dysfunction, male or female  **Exclusion**: hypersensitivity to the tape, vertebral compression fracture, neurologic deficit, symptoms of vertigo or dizziness, cardiopulmonary disease with decreased activity tolerance, sensory disturbances | N = 90 | **T_1_**: KT + conventional therapy (stretching & strengthening exercises)  (n=30)  **T_2_**: KT + conventional therapy + muscle energy technique  (n=30)  **T_3_**: conventional therapy + muscle energy technique  (n=30) | **Pain**:  VAS  **Disability**: n/a  **Functional**: Lumbar ROM  **Time points**: 4 week intervention duration   1. Baseline 2. Post treatment | **Pain**:  All groups demonstrated significant improvement from baseline, however no significant difference between groups was observed  **Functional**:  All groups demonstrated significant improvement from baseline, however no significant difference between groups was observed | A combination between muscle energy techniques and KT with conventional therapy appears to improve pain and ROM in  patients with chronic low back dysfunction. | 4 |
| Grześkowiak (2018) [137] | **Age**: 20-55  **Diagnosis**: chronic low back pain secondary to lumbar disc herniation    **Inclusion**: unilateral or central disk herniation at L4/L5 and/or L5/S1  vertebra levels on MRI, RMDQ score ≥ 4, lumbosacral (with or without referring to leg) pain ≥ 3 months  **Exclusion**: disc degenerations or herniation to other than specified levels of lumbar spine, systemic  or orthopedic diseases, pregnancy, coexisting pathologies  of spinal column and pelvis, previous spine or pelvis surgery, previous KT therapy, no physical therapy referral at the study time, body mass index >30 | N = 38 | **T_1_**: KT  (n=19)  **T_2_**: placebo tape  (n=19) | **Pain**:  VAS  PPT  **Disability**:  RMDQ  **Functional:**  surface EMG  ROM  back extension maximum voluntary isometric contraction  **Time points**: 7 day intervention duration   1. Baseline 2. Post-treatment | **Pain**:  Subjective pain intensity in the KT group decreased some, similar to the placebo group.  **Disability:**  Both groups had similar improvement in  disability scores  **Functional:**  KT application did not change trunk range of motion.  No meaningful improvement was noted in back muscle force. | Seven-day KT does not normalize lumbar paraspinal muscle function and is not superior to placebo in reducing disability and pain intensity in patients with lumbar disc herniation. | 7 |
| Kachanathu  (2014) [149] | **Age**: 27-42  **Diagnosis**: non-specific low back pain  **Inclusion**: pain >3 months  **Exclusion**: no other pathological problems | N = 40 | **T_1_**: conventional physiotherapy with KT  (n=20)  **T_2_**: conventional physiotherapy (stretching exercises for back and abdominal strengthening)  (n=20) | **Pain**:  VAS  **Disability**:  RMDQ  **Functional**: Trunk flexion and extension  Schober’s test  **Time points**: 4 week intervention   1. Baseline 2. Post treatment | **Pain**:  Significant differences in pre- and post-intervention. However, between groups showed no significant differences in pre- and post-intervention.  **Disability**:  Significant differences in pre- and post-intervention. However, between groups showed no significant differences in pre- and post-intervention.  **Functional**:  Significant differences in pre- and post-intervention. However, between groups showed no significant differences in pre- and post-intervention. | Physical therapy that involves stretching of the back, hamstring, and iliopsoas muscles with KT or without KT may be effective in relieving LBP, increasing the ranges of pain-free active trunk flexion and extension, and improving RMDQ. | 3 |
| Kamali  (2017) [150] | **Age**: 20-45  **Diagnosis**: non-specific low back pain  **Inclusion**: pain ≥ 3 months, NRS ≥ 30 out 100, ODI ≥ 8  **Exclusion**: sciatalgia, disk herniation, spinal canal stenosis, spondylolisthesis, previous lumbar surgery, history of neurological, rheumatoid and psychological diseases, physiotherapy during the last three months, or used opioid and analgesic drugs in the last 72 h prior to the trial. Subjects with contraindication to spinal manipulation | N = 42 | **T_1_**: spinal manipulation  (n=21)  **T_2_**: spinal manipulation and KT  (n=21) | **Pain**:  NRS  **Disability**:  ODI  **Functional**: Trunk flexor and extensor endurance  **Time points**: 24-hour of taping intervention   1. Baseline 2. Post treatment 3. 1 day follow-up 4. 1 week follow-up 5. 1 month follow-up | **Pain**:  NRS decreased significantly after the interventions in both groups, however, there was no statistically significant difference between the two groups  **Disability**:  ODI decreased significantly after the interventions in both groups, however, there was no statistically significant difference between the two groups  **Functional**:  Endurance of the trunk flexors and extensors increased significantly after the interventions in both groups, however, there was no statistically significant difference between the two groups | KT might not add any extra positive effect on spinal manipulation in treating athletes with chronic non-specific low back pain. | 6 |
| Kaplan  (2016) [139] | **Age**: 18-40  **Diagnosis**: pregnancy related low back pain  **Inclusion**: any parity, gestational age between 10 and 30 weeks, low back pain experienced from T12 to the gluteal fold without leg pain,  VAS ≥ 4  **Exclusion**: scoliosis, spine injuries, ankylosing spondylitis or rheumatoid arthritis, intervertebral disc pathology, a history of low back pain prior to pregnancy, twin pregnancy or fetal anomaly; and any uncontrolled medical condition | N = 65 | **T_1_**: KT and paracetamol  (n= 33)  **C**: paracetamol (1500mg/day for 5 days)  (n= 32) | **Pain**:  VAS  **Disability**:  RMDQ  **Functional**: n/a  **Time points**: 5 day duration   1. Baseline 2. Post treatment | **Pain**:  In both groups, pain intensity during rest and pain intensity during movement were significantly reduced at day 5 compared with baseline. KT group was significantly superior than the control group  **Disability**:  In both groups, disability was significantly reduced at day 5 compared with baseline.  KT group was significantly superior than the control group | When compared with paracetamol therapy alone, combined KT and paracetamol therapy seems to be more effective to reduce pain and improve functional ability in the treatment of pregnancy-related low back pain.  KT can be used as a complementary treatment method to achieve effective control of pregnancy-related low back pain. | 6 |
| Kelle  (2016) [136] | **Age**: 18-65  **Diagnosis**: acute non-specific low back pain  **Inclusion**: pain between the 12^th^ rib and buttock crease with or without leg pain, pain <6 weeks duration, NRS ≥ 4  **Exclusion**: known or suspected serious spinal pathology, spinal surgery within the preceding six months, serious comorbidities preventing prescription of paracetamol, physical therapy for low back pain in previous six months | N = 109 | **T_1_**: KT with minimal care  (n=54)  **T_2_**: minimal care (reassurance, booklet on back pain, as needed paracetamol)  (n=55) | **Pain**:  NRS  **Disability**:  ODI  **Functional**: Number of paracetamol tablets consumed per day  **Time points**: 12 day intervention   1. Baseline 2. Post-treatment 3. 4 week follow-up | **Pain**:  Statistically significant improvements were found in both groups regarding pain intensity at the 12^th^ day and at the fourth week of intervention differences between the 12^th^ day and fourth week were not significant. Pain reduction was significantly better in the KT group  **Disability**:  Statistically significant differences between the groups at the 12th day of intervention, the difference was borderline not significant at the fourth week  **Functional**:  Statistically significant differences in the consumption of paracetamol were seen at days 1–4 and 5 8 | The study demonstrated the positive effects of the KT application in patients with acute non-specific low back pain.  KT can be used as a complementary treatment method to achieve fast and effective control of pain.  KT may reduce drug consumption in patients with acute low back pain. | 7 |
| Keles  (2017) [138] | **Age**: 18-45  **Diagnosis**: lumbar disc herniation  **Inclusion**: lumbar disc herniation proven by medical history, physical examination and confirmed by MRI, symptomatic ≥ 3 months, self-reported activity pain levels of ≥ 3 on numeric rating scale  **Exclusion**: severe degenerative disorders, concomitant spinal stenosis proven by physical examination and confirmed by imaging, congenital spinal abnormalities, coexistent or preexisting spine pathology (spondylolysis, spondylolisthesis, infection, tumor, fracture) history of lumbar surgery, trauma or malignancy, inflammatory low back pain, motor weakness, urinary/fecal incontinence due to lumbar disc herniation, cauda equina syndrome requiring emergency surgical decompression, neurological disease, dermatitis or pre-existing skin lesion over taping area, use or prior knowledge of KT method, pregnancy | N = 52 | **T_1_**: KT  (n=29)  **T_2_**: Placebo  (n=23) | **Pain**:  Numeric rating scale: at rest and activity  **Disability**:  ODI  Health assessment questionnaire  **Functional**: Finger to floor distance  Lumbar Schober test  Number of paracetamol tablets taken  **Time points**: 3 week intervention   1. 1 week 2. 2 weeks 3. 3 weeks 4. 6 weeks 5. 12 weeks | **Pain**:  Despite significant improvements of pain in KT group, there was no significant difference between two groups in terms of percentage changes from baseline  **Disability**:  Despite significant improvements of disability in KT group, there was no significant difference between two groups in terms of percentage changes from baseline  **Functional**:  No significant difference  for the percentage changes from baseline in finger to floor distance and lumbar schober between two groups | Analgesic need in  KT group at long term follow up period was significantly less than placebo taping group.  KT reduces the pain level and improves the disabilities of patients with chronic  low back pain due to lumbar disc herniation. When  KT compared with placebo taping, no significant differences regarding pain, disability and lumbar flexion were detected between groups. | 7 |
| Köroğlu (2017) [151] | **Age**: 18 and older  **Diagnosis**: chronic low back pain  **Inclusion**: pain ≥ 3 months  **Exclusion**: receiving any other treatment for low back pain at the time of the study, allergic reaction to the taping, any contraindications for physical therapy modalities or exercise, primary or metastatic neoplasm on spine or other tissues, previously spinal surgery, infectious pathologies of the spine, inflammatory disease of the spine, radiculopathy, prolonged immobilization that could lead to muscle atrophy, or steroids and similar drugs usage | N = 60 | **T_1_**: KT  (n=20)  **T_2_**: Placebo tape  (n=20)  **C**: Control  (n=20) | **Pain**:  VAS  **Disability**:  ODI  **Functional**: Trunk flexibility - Sit and reach test  Biering-Sorenson test  **Time points**: 2 week intervention   1. Baseline 2. Post-treatment | **Pain**:  Placebo and control groups had statistically similar results regarding pain scores while the KT group was statistically superior to both groups  **Disability**:  The highest average change in disability scores was in the taping group  **Functional**:  Placebo and control groups had statistically similar results regarding flexibility and endurance scores while the KT group was statistically superior to both groups | A greater improvement was obtained in pain, functionality, lumbar mobility and muscle endurance with the application of KT addition to the electrotherapy and exercise methods which are commonly used in the treatment of chronic low back pain.  The results from this study suggested that the use of KT is an effective supportive method for chronic low back pain rehabilitation. | 5 |
| Luz Junior (2015) [152] | **Age**: 18-80  **Diagnosis**: chronic non-specific low back pain  **Inclusion**: back pain of mechanical  origin, apparently without a defined cause, ≥ 12 weeks duration.  **Exclusion**: skin diseases, contraindication due to the use of the tape, serious spinal pathologies (tumor, inflammatory disease, fracture), nerve root compromise, pregnancy, subjects who had physical therapy treatment in the past six months, subjects who had used or had prior knowledge of the KT method | N = 60 | **T_1_**: KT  (n=20)  **T_1_**: Placebo (Micropore)  (n=20)  **C**: no treatment  (n=20) | **Pain**:  NRS  **Disability**:  RMDQ  **Functional**: n/a  **Time points**: 2 day intervention   1. Baseline 2. Post-treatment 3. 7 days | **Pain**:  There was no observable difference between groups at 48 hours or at 7 days  **Disability**:  A statistically significant difference was observed  between the KT and control  group for the disability outcome at the 48-hour  follow up  No differences were detected between the KT and placebo groups for disability at either time point. | KT showed similar results to Micropore taping in pain and disability at 2 days and at 7 days  KT was superior only when compared to the control group  for the disability outcome at post-treatment assessment.  The therapeutic effects of the KT are similar to the placebo effect and these results suggest that physical therapists should avoid this type of therapy. | 8 |
| Macedo (2019) [153] | **Age**: 18-50  **Diagnosis**: low back pain  **Inclusion**: duration > 3 months  **Exclusion**: diagnosis of fractures or tumours in the spine, ankylosing spondylitis, disc herniation, spondylolisthesis with neurological involvement, lumbar stenosis, previous spinal surgery, fibromyalgia and any central or peripheral neurological diseases. pregnant,  were on their menstrual cycle or the premenstrual period, BMI > 30, NPRS < 2 in the last 24 hours of the first evaluation, corticosteroids use last 2 weeks, anti-inflammatory medication use in last 24 hours, allergy to KT during a test, undergone prior treatment with this technique in the lumbar region, lack of understanding of the instructions, inadequate performance of the evaluations | N = 108 | **T_1_**: KT with tension  (n=27)  **T_2_**: KT no tension  (n=27)  **T_3_**: Micropore tape (n=27)  **C**: Control  (n=27) | **Pain**:  NPRS  **Disability**:  RMDQ  **Functional**:  Trunk ROM  Trunk Extensor Strength  **Time points**: single application   1. Baseline 2. 3 days post-treatment 3. 10 days post-treatment | **Pain**:  Significant improvement in NPRS was observed in the KT with tension and KT no tension compared to control at 3 days after tape application.  **Disability**:  There was significant improvement in RMDQ for KT with tension compared to control at 10 days  **Functional**:  There was no significance difference between groups and no interaction between group and time | KT reduces pain and disability in patients  with chronic non-specific low back pain.  There is no difference between the use of KT with or without tension for pain.  The Micropore group showed no differences compared with the KT or control groups. | 8 |
| Mohamed (2018) [140] | **Age**: 18-35  **Diagnosis**: Postpartum low back pain    **Inclusion**: delivered by normal vaginal delivery without epidural anesthesia, breast feeding, body mass  index ≤ 30, housewives, moderate education level, low back pain from T12 to the gluteal fold with or without radiation to the knee  **Exclusion**: low back and/or pelvic pain prior to pregnancy, pain radiating below the knee, congenital anomalies of the spine, rheumatic disease affecting the locomotion system, disc prolapse, spondylosis, spondylolisthesis confirmed by  x-ray, previous surgery of the lumbar spine or pelvis | N = 30 | **T_1_**: KT + exercise  (n=15)  **T_2_**: Exercise  (n=15) | **Pain**:  VAS  **Disability**: n/a  **Functional**: Back Pain Function Scale (BPFS)  **Time points**: 2 week intervention duration   1. Baseline 2. Post treatment | **Pain**:  There was significant reduction of VAS in favor of KT + exercise  **Functional**:  There was a significant increase of BPFS in favor of KT + exercise | KT and postural correction exercises can be used as effective and safe methods for the treatment of postnatal women with backache and could be considered as a viable alternative or complement to medical treatment of low back pain. | 6 |
| Neamat Allah  (2019) [158] | **Age**: not specifically described  **Diagnosis**: Sacroiliac joint dysfunction    **Inclusion**: female, positive in at least 3 of 4 clinical tests: standing flexion test, supine to long sitting test, prone knee flexion test and palpation of posterior superior iliac spine heights for symmetry on sitting, anterior innominate rotation greater than 2.5◦  compared to the other side, unilateral pain over the sacroiliac region, at 3 month duration, aggravated by  standing and walking  **Exclusion**: nerve root involvement,  muscle weakness involving more than one muscle with the same nerve root innervation in the ipsilateral lower extremity, diminished lower extremity reflexes, previous back or hip surgery, primary symptoms of anterior or lateral hip pain | N = 32 | **T_1_**: Rigid tape  (n=17)  **C**: no treatment  (n=15) | **Pain**:  VAS  SIJ pain provocation testing  **Disability**: n/a  **Functional**: Innominate rotation measurement  Gillet test  Sitting Forward Flexion  Active hip internal and external rotation  **Time points**: 2 week intervention duration   1. Baseline 2. Post treatment | **Pain**:  Pain decreased in the rigid tape group but not in the control group  The number of participants with positive pain provocation tests at follow-up was significantly smaller in the rigid tape group compared to the control  **Functional**:  The number of participants with positive mobility tests at follow-up was significantly smaller in the Rigid tape group compared to the control  Degree of innominate rotation decreased in the rigid tape group but not in the control group  No differences were observed across time for active hip internal and external rotation range in either group | Rigid taping may be an effective treatment for pain and mobility deficits related to anterior innominate dysfunction. | 5 |
| Paoloni  (2011) [154] | **Age**: 30-80  **Diagnosis**: chronic low back pain    **Inclusion**: pain > 12 weeks, fail to achieve flexion-relaxation in lumbar muscles during trunk flexion  **Exclusion**: clinical signs of radiculopathy, lumbar stenosis, spondylolisthesis, previous spinal surgery, corticosteroid injection in the last two weeks, central and/or peripheral nervous system diseases | N = 39 | **T_1_**: KT and exercise  (n=13)  **T_2_**: KT  (n=13)  **T_3_**: exercise only  (n=13) | **Pain**:  VAS  **Disability**:  RMDQ  **Functional**: none  **Time points**: 4 week intervention   1. Baseline 2. Post treatment | **Pain**:  Significant reduction in VAS scores from baseline in all three groups.  **Disability**:  RMDQ scores reduced in all three groups as well, significant for exercise group alone. | KT leads to pain relief and lumbar muscle function normalization shortly after application and persists over a short follow-up period. | 7 |
| Parreira  (2014) [155] | **Age**: 18-80  **Diagnosis**: chronic nonspecific low back pain  **Inclusion**: pain > 3 months, pain caused by herniated disc,  **Exclusion**: any contraindication to physical exercise, according to the guidelines of the American College of Sports Medicine, serious spinal pathology, nerve root compromise, serious cardiopulmonary conditions, pregnancy or any contraindications to the use of  taping (such as skin allergy) | N = 148 | **T_1_**: KT  (n=74)  **T_2_**: sham taping  (n=74) | **Pain**:  NRS  **Disability**:  RMDQ  **Functional**: Global perceived effect scale  **Time points**: 4 week intervention   1. Baseline 2. Post treatment 3. 12 week follow-up | **Pain**:  No significant between-group differences were observed for pain intensity at four weeks  **Disability**:  No significant between-group differences were observed for RMDQ at four weeks  **Functional**:  A significant, but small, difference in favor of the intervention group for global perceived effect at four weeks, but not at 12 weeks | KT did not reduce pain or disability in people with chronic non-specific low back pain. There was a small improvement in global perceived effect after four weeks, but this was not sustained to 12 weeks. | 9 |
| Tuttle  (2018) [141] | **Age**: 18 and older  **Diagnosis**: postpartum diastasis recti abdominis    **Inclusion**: primiparous or multiparous females, 6 to 12 weeks postpartum, palpable separation of rectus abdominis muscles  **Exclusion**: Edinburgh Postnatal Depression Questionnaire score of ≥ 13, Pelvic Floor Distress Inventory score > 75% | N = 30 | **T_1_**: transverse abdominis (TRA) exercise  (n=10)  **T_2_**: KT  (n=8)  **T_3_**: KT + TRA  (n=5)  **C**: control  (n=7) | **Pain**: n/a  **Disability**:  RMDQ  **Functional**: Inter-recti distance  Pelvic Floor Distress Inventory (PFDI-20)  **Time points**: 3 month intervention duration   1. Baseline 2. Post treatment | **Disability**:  No significant difference in RMDQ score between the groups at any time point  **Functional**:  KT + TRA group and TRA exercise only groups  were both significantly better at decreasing inter-recti distance at rest and with head lift than either the tape group or the control group, but adding taping did not improve  the exercise results  No significant difference in PFDI-20 score between the groups at any time point | Groups with a TRA exercise component (TRA exercise and taping and TRA exercise alone) had the largest reduction in inter-recti distance at rest and with head lift.  These data provide preliminary evidence that taping alone has minimal effect on reducing inter-recti distance for women with diastasis recti abdominis. | 7 |
| Uzunkulaoğlu  (2018) [156] | **Age**: 18-30  **Diagnosis**: chronic non-specific low back pain    **Inclusion**: ≥3 VAS, pain in the area  between the 12th rib and buttock crease, >12-week duration.  **Exclusion**: elevated full blood count, elevated erythrocyte sedimentation rate (ESR), elevated C-reactive protein (CRP), elevated biochemical markers, presence of skin diseases, contraindication to use of the tape, pregnancy, known or suspected serious congenital spinal pathology, spinal surgery history, lumbar disc herniation, rheumatoid arthritis,  spondyloarthropathy diagnosis, physical therapy for low back pain during prior six months | N = 60 | **T_1_**: KT + daily home exercise program  (n=30)  **T_2_**: Sham taping (no tension) + daily home exercise program  (n=30) | **Pain**:  VAS  **Disability**:  ODI  **Functional**: Schober’s test  Hand ground distance  Lumbar ROM  **Time points**: 15 day intervention duration   1. Baseline 2. 1 month (15 days post-treatment) 3. 6 month follow-up | **Pain**:  There was statistically  significant improvement in the KT group at 1 month follow-up only  **Disability**:  There was statistically  significant improvement in the KT group at 1 month and was sustained at 6 month follow-up  **Functional**:  There was statistically  significant improvement in the KT group for all functional measures at 1 month, while Schober and hand-ground distance was sustained at 6 month follow-up | KT provided significant improvements in pain, ROM and disability in chronic  non-specific low back pain in the short- term. These  positive effects were sustained for ROM and disability  at long term, but not for pain. | 8 |
| Velasco-Roldán  (2017) [157] | **Age**: 18-45  **Diagnosis**: nonspecific low back pain  **Inclusion**: pain > 6 weeks before the study or on-and-off spinal pain with ≥ 3 episodes of low back pain during the year before the study, each lasting more than 1 week  **Exclusion**: previous spinal surgery, history of spinal or pelvic fracture, severe trauma and/or injuries related to an automobile accident, osteoarthritis and/or fractures of the lower extremities, degenerative,  systemic, rheumatic, and/or tumoral disorders, received manual therapy within 8 weeks before data collection or during the study, having received KT as an intervention procedure for low back pain; under pharmacological treatment to relieve pain, low back pain associated with radicular pain and/or radiculopathy with presence of neurologic signs, and having any allergies that would prevent the placement of a bandage | N = 75 | **T_1_**: standard KT tension  (n=26)  **T_2_**: increased KT tension  (n=25)  **T_3_**: no KT tension  (n=24) | **Pain**:  Pressure pain threshold  **Disability**: n/a  **Functional**: Trunk flexion ROM  **Time points**: intervention duration   1. Baseline 2. 10 minutes after KT application 3. 24 hours after KT application 4. Immediately after KT removal (~25 hours) | **Pain**:  No differences were found for pressure algometry when considering the whole sample (P > .05)    Higher pain thresholds over the gluteus medius were observed in men  **Functional**:  Significant difference was observed only between those who underwent an increased KT tension treatment and the no tension group in the evaluation after 24 hours, for the left backsaver sit-and-reach test  Double inclinometry evaluations were not statistically significant | The use of different percentages of KT tension does not seem to influence its impact on pain sensitivity and lumbar mobility in chronic low back pain. | 6 |

**APPENDIX 2H: EVIDENCE TABLE FOR RANDOMIZED CONTROLLED TRIALS OF THORACIC PAIN**

| Study | Population description | Sample size | Interventions | Outcomes/time points | Results | Key author conclusions | PEDro Score |
| --- | --- | --- | --- | --- | --- | --- | --- |
| Bautmans (2010) [159] | **Age**: 69-83  **Diagnosis**: thoracic pain  **Inclusion**: scheduled for 3-month intravenous pamidronate treatment  **Exclusion**: Paget’s disease, rheumatoid arthritis, ankylosing spondylitis, cancer, and cognitive or physical inability to understand and/or participate in the test procedures, recent (< 3 months) and/or symptomatic vertebral fractures | N = 48 | **T_1_**: rehabilitation (thoracic mobilization, KT, posture exercises)  (n= 29)  **C**: control waiting list  (n= 19) | **Pain**:  VAS  **Disability**:  Quality of Life (Qualeffo-41)  **Functional**: Thoracic kyphosis degree  **Time points**: 3 month duration   1. Baseline 2. Post treatment | **Pain**:  No significant difference between groups was observed  **Disability**:  Mental health perception worsened slightly but significantly in the rehabilitation group  **Functional**:  Degree of thoracic kyphosis was significantly reduced in the rehab group compared to the control and in comparison to those non-compliant | Three months of rehabilitation with manual mobilization can attenuate thoracic kyphosis in elderly patients with osteoporosis. Impact on back pain and quality of life remains unclear. | 7 |
| Bulut  (2019) [160] | **Age**: not specifically described  **Diagnosis**:Postmenopausal osteoporosis-associated thoracic kyphosis    **Inclusion**: not specifically described  **Exclusion**: not specifically described | N = 47 | **T_1_**: KT [for posture correction] + home exercises (n=23)  **T_2_**: home exercises (strengthening, stretching exercises)  (n=24) | **Pain**:  VAS  **Disability**: n/a  **Functional**:  Static Balance  Dorsal kyphosis angle  **Time points**: 3 week intervention   1. Baseline 2. Week 3 3. Week 6 4. Post-Treatment | **Pain**:  There was improvement for both groups, though there were no significant differences between the groups in terms of the values recorded.  **Functional**:  No significant difference between groups for static balance measurements values. | Application of KT may have short-term positive effects on pain, but is unlikely to have significant effects on kyphosis angle or balance for women with osteoporosis.  Positive changes seen in kyphosis angle and balance 30 min after  taping are short-lived. | 6 |

**APPENDIX 2I: EVIDENCE TABLE FOR RANDOMIZED CONTROLLED TRIALS OF THE CERVICAL SPINE**

| Study | Population description | Sample size | Interventions | Outcomes/time points | Results | Key author conclusions | PEDro Score |
| --- | --- | --- | --- | --- | --- | --- | --- |
| Ata  (2019) [171] | **Age**: 18-65  **Diagnosis**: Myofascial pain syndrome of the neck and shoulder    **Inclusion**: ≥ 1 painful myofascial trigger point (trapezius, levator scapula, rhomboids, supraspinatus and infraspinatus), Simons et al was used for myofascial pain syndrome diagnosis whereby 5 major and at ≥ 1 minor criteria are required for clinical diagnosis  **Exclusion**: pregnant, lactating women, fibromyalgia, mental disorder, history of lidocaine allergy | N = 76 | **T_1_**: Lidocaine  (n=26)  **T_2_**: Lidocaine + sham KT (no tension)  (n=25)  **T_3_**: Lidocaine + KT (tension)  (n=25) | **Pain**:  VAS  **Disability**: n/a  **Functional**: SF-12  **Time points**: 1 week intervention duration   1. Baseline 2. Post treatment 3. 1 week follow up 4. 4 weeks follow up | **Pain**:  There was significant improvement in pain in the short-term for KT + lidocaine compared the other groups  **Functional**:  There was significant improvement in the physical component of function in the short-term for KT + lidocaine compared to the other groups, however, mental component summary was not | KT may be useful to increase the efficacy of myofascial trigger point lidocaine injection in myofascial pain syndrome of the neck. | 6 |
| Ay  (2017) [172] | **Age**: 18 and older  **Diagnosis**: myofascial pain of levator scapulae  **Inclusion**: trigger point criteria described by Travell and Simons, presence of at least one active trigger point located in levator scapula muscle, symptom duration of at least ≥3 months  **Exclusion**: fibromyalgia syndrome, cervical disc lesion, radiculopathy, myelopathy, recent trigger point injection or participating in a physical treatment program within the last 6 months, neurologic and inflammatory diseases, pregnancy, history of neck or shoulder surgery | N = 61 | **T_1_**: KT + exercise  (n=31)  **C**: sham taping + exercise  (n=30) | **Pain**:  VAS  Pain pressure threshold  **Disability**:  Neck Pain Disability Scale  **Functional**:  Cervical ROM  **Time points**: 15 day intervention   1. Baseline 2. Post-treatment | **Pain**:  Both groups improved, however, statistical significance favored KT for VAS and PPT  **Disability**:  Both groups improved at post-treatment  **Functional:**  Both groups improved with statistical significant favoring KT for cervical flexion-extension only | KT leads to improvements on pain, PPT, and ROM, but not in disability in short period.  KT can be used as an alternative therapy in the treatment of myofascial pain syndrome of levator scapulae | 7 |
| Azatcam (2017) [173] | **Age**: 18-65  **Diagnosis**: myofascial pain syndrome of upper trapezius  **Inclusion**: ≥ 1sensitive points on stiff bands at ipsilateral posterolateral cervical paraspinal areas, mastoid process or temporal area, twitch response by palpation of sensitive point on a stiff band, limitation of motion by lateral bending of the cervical spine on contralateral side  **Exclusion**: injection to the myofascial trigger point, use of  physical therapy modalities last year, history of acute trauma, inflammatory joint or muscle disease, infection, malignancy, cervical radiculopathy or myelopathy, symptoms and findings fulfilling the 1990 American college of rheumatology criteria for fibromyalgia, poor cooperation | N = 69 | **T_1_**: Transcutaneous Electrical Nerve Stimulation (TENS) + exercise  (n=23)  **T_2_**: KT + exercise  (n=23)  **C**: Exercise  (n=23) | **Pain**:  VAS  PPT  **Disability**:  NDI  **Functional**:  Cervical ROM  **Time points**: 2 week intervention   1. Baseline 2. Post-treatment 3. 3 month follow-up | **Pain**:  VAS and pain threshold significantly improved in all groups post-treatment and at 3 months. Between group comparison favored KT at post-treatment and at 3 months no difference between KT and TENS  **Disability**:  Neck Disability Index was significantly improved at post-  treatment and 3 months in all groups  **Functional:**  Contralateral lateral flexion was significantly improved at post-treatment and at 3 months in all groups | The addition of TENS or KT to the exercise therapy resulted in more significant improvement compared to exercise therapy alone with a more pronounced improvement in KT group compared to the TENS group in the early period.  KT was found to be more effective in decreasing the pain and had the advantage of being beneficial in acute painful periods in myofascial pain syndrome patients. | 6 |
| Dawood  (2013) [161] | **Age**: 22-36  **Diagnosis**: mechanical non-specific neck pain  **Inclusion**: pain > 3 months, cervical lordotic curve < 34° , NDI > 5  **Exclusion**: cervical disc problems or cervical spondylosis, history of previous neck trauma or head injuries, ankylosing spondylitis, osteoporosis, cervical rib, post-surgical neck conditions | N = 54 | **T_1_**: KT  (n=19)  **T_2_**: Cervical traction  (n=19)  **C**: Control  (n=16) | **Pain**:  VAS  **Disability**:  NDI  **Functional**: Absolute Rotatory Angle  **Time** **points:** 4 weeks intervention   1. Baseline 2. Post treatment | **Pain**:  VAS was significantly for KT and cervical traction groups compared to sham  **Disability**:  KT and cervical traction groups demonstrated significant decrease in NDI compared to control group  **Functional**:  KT and cervical traction groups demonstrated significant increase in absolute rotary angle compared to control group | KT and cervical traction posture pump are equally effective in improving cervical curvature, pain intensity and function neck disability in patients with mechanical neck disorders compared to exercises program alone which was the least effective. | 5 |
| Doğan (2019) [174] | **Age**: 20-50  **Diagnosis**: Myofascial pain of the upper trapezius    **Inclusion**: unilateral upper trapezius muscle trigger point, symptom duration ≥ 3months, NPRS ≥ 4 at rest or activity, all criteria had to be met for the diagnosis of active myofascial trigger point  **Exclusion**: prior neck surgery, cervical radiculopathy, neurologic deficit, history of any treatment for trigger points in the upper trapezius muscle region in the last six months, pregnancy, infection, irritation of the skin, open wound in the region of the upper trapezius, presence of blood dyscrasias, current anticoagulant therapy, malignancy, advanced cervical spine degeneration, fibromyalgia, kyphosis and/or scoliosis epilepsy, polyacrylate allergy. | N = 42 | **T_1_**: KT  (n=23)  **T_2_**: Dry needling  (n=19) | **Pain**:  VAS  PPT  **Disability**:  NDI  **Functional**: Cervical ROM  **Time points**: 15 day intervention duration   1. Baseline 2. 5th day treatment 3. Post treatment 4. 4 weeks | **Pain**:  While both groups improved from baseline, there was no significant difference was found between the groups for pain in the short- and medium-term, except dry needling did not significantly improve in the short-term  **Disability**:  While both groups improved from baseline, there was no significant difference was found between the groups for pain in the short- and medium-term  **Functional**:  While both groups improved from baseline, there was no significant difference was found between the groups for pain in the short- and medium-term | KT and dry needling appeared to have an equally positive effect in the treatment of myofascial pain syndrome of the upper trapezius muscle, especially in pain reduction.  KT may be a choice to treatment myofascial pain syndrome of the upper trapezius in patients who do not desire to be needled or who have contraindications to other treatments including dry needling. | 5 |
| el-Abd (2017A) [162]  [J Bodywork Movement Ther] | **Age**: 20 – 40  **Diagnosis**: mechanical neck dysfunction  **Inclusion**: NDI>15  **Exclusion**: cervical surgery/trauma, radiculopathy/myelopathy, current treatment for cervical dysfunction, definite cervical disorder | N = 46 | **T_1_**: KT  (n=22)  **C**: Postural exercises  (n=23) | **Disability**:  NDI  **Functional**:  sEMG activity (EMG-MF) for upper trapezius and levator scapulae  **Time points**: 4 weeks intervention   1. Baseline 2. Post treatment | **Disability**:  Both groups displayed significant reduction in NDI and significant reduction in muscle fatigue from baseline to 4 weeks.  **Functional**:  There was no significant interaction for either upper trapezius sEMG | Postural correction exercises and KT exhibited similar reduction in axioscapular muscles fatigue.  Reduction of neck disability was greater in patients received KT. Therefore, KT may be an alternative treatment option in the treatment of mechanical neck dysfunction | 8 |
| el-Abd (2017B) [163]  [J Sports Med Phys Fitness] | **Age**: 18-40  **Diagnosis**: mechanical neck pain  **Inclusion**: NDI > 15  **Exclusion**: cervical surgery/trauma, cervical radiculopathy or myelopathy, current medical or physical treatment, allergy to KT and pathologies such as skin diseases, inflammatory diseases, neurological diseases, congenital diseases, fractures, dislocations, neoplasms, and infection. | N = 46 | **T_1_**: KT  (n=23)  **T_2_**: Postural correction exercise  (n=23) | **Pain**:  VAS  **Disability**:  n/a  **Functional**: upper trapezius and levator scapulae (axioscapular muscles) EMG root mean score  **Time points:** 4 weeks intervention   1. Baseline 2. Post treatment | **Pain**:  KT group experienced more pain reduction than postural corrections, however, effect size was small. Within group testing revealed significant reduction of pain in both groups  **Functional**:  There was no significant interaction for either upper trapezius or levator scapular root mean score. Within group testing revealed significant reduction in root mean score in both groups for upper trapezius and levator scapulae | Postural correction exercises or receiving an application of  KT exhibited similar reduction in axioscapular muscle activities. However, reduction of pain was greater in patients received KT.  KT may be an alternative treatment option in the treatment of mechanical neck dysfunction. | 8 |
| el-Abd (2017C) [164]  [IJTR] | **Age**: 18-40  **Diagnosis**: mechanical idiopathic neck pain  **Inclusion**: negative compression and distraction tests, NDI > 15, cervical lordotic angle < 34 degrees  **Exclusion**: history of whiplash or cervical surgery, cervical radiculopathy or myelopathy, fibromyalgia syndrome, previous postural correction therapy or KT applications, current medical or physical treatment, vertebrobasilar insufficiency or inflammatory disorders | N = 45 | **T_1_**: KT  (n=23)  **T_2_**: Postural exercises  (n=22) | **Pain**:  VAS  **Disability**:  NDI  **Functional**: cervical curvature  upper trapezius EMG root mean score  **Time** **points:** 4 weeks intervention   1. Baseline 2. Post treatment | **Pain**:  KT group experienced more pain reduction than those who performed postural correction exercises. Within group assessment for KT and posture correction found significant reduction in pain, disability and upper trapezius muscle activities and an increase in cervical curvature  **Disability**:  No significant interaction for NDI  **Functional**:  No significant interaction for cervical curvature or upper trapezius muscle activity | KT and postural correction exercises are effective in improving cervical curvature, normalizing cervical muscle activity and reducing both pain intensity and functional neck disability in patients with mechanical neck dysfunction. KT appeared to be more effective in reducing pain intensity. | 7 |
| González-Iglesias  (2009) [180] | **Age**: 26-40  **Diagnosis**: acute whiplash injury  **Inclusion**: neck pain due to motor vehicle accident within 40 days of injury, Quebec Task Force Classification of whiplash associated disorder, without evidence of conduction loss on clinical neurological examination  **Exclusion**: concussion, loss of consciousness, head or upper quadrant injury during motor vehicle accident, treatment prior to their accident for neck pain, history of whiplash, neck pain, headaches, psychiatric or psychologic condition, neurologic or circulatory disorders, somatic condition (eg, fibromyalgia syndrome), current claim for litigation or compensation | N = 41 | **T_1_**: KT to cervical spine  (n=21)  **T_2_:** Placebo KT application  (n=20) | **Pain**:  NPRS  **Disability**:  n/a  **Functional**:  Cervical ROM  **Time points:** Immediate post tape application   1. Baseline 2. Post treatment 3. 24-hour follow-up | **Pain**:  Statistically significant differences in pain, but did not meet the minimum clinically important difference  **Functional**:  Statistically significant differences in cervical ROM between groups, but did not meet the minimum clinically important difference | Acute whiplash associated disorder patients show statistically significant improvements immediately after application and at 24-hour follow-up in pain and cervical ROM, but these changes are small and may not be clinically meaningful. | 8 |
| Halski  (2015) [175] | **Age**: 18 – 26  **Diagnosis**: latent trigger points of upper trapezius  **Inclusion**: pain during examination of latent, MTrPs in the upper part of the trapezius muscle, absence of skin allergies  **Exclusion**: history of upper limb, back or neck severe injury in the last 12 months, surgical intervention, upper limb fractures, neurological diseases or musculoskeletal disorders, pharmacological treatment at present, infection, open wound, rash, decreased blood circulation in the treatment area, a pacemaker, or epilepsy | N=105 | **T_1_**: Cross tape (n=24)  **T_2_**: KT  (n=25)  **C**: Adhesive, non-elastic medical tape with no therapeutic influence  (n=24) | **Pain**:  VAS  **Disability**: n/a  **Functional**:  Surface EMG for upper trapezius  Mobility of the cervical spine  **Time points**: 3 days intervention   1. Baseline 2. Post treatment, 3. 24 hours follow-up | **Pain**:  Significantly lower VAS scores following the intervention in all groups  **Function**:  sEMG: no significant differences were observed in bioelectrical activity between pre-, post-, and follow-up results  Cervical mobility: flexion ROM significantly improved for all groups post-treatment and at follow-up  In KT group, significant improvement in lateral flexion movement was observed post-treatment | All three types of tapes fail to influence the resting bioelectrical activity of upper trapezius muscle and may not lead to a reduction in muscle tone in the case of MTrPs.  KT application reduces the subjective pain sensation, which confirms the scientific reports about its analgesic influence. | 7 |
| Hayta  (2016) [176] | **Age**: 20-60  **Diagnosis**: myofascial pain syndrome of upper trapezius  **Inclusion**: duration of symptoms ≥ 3 months, palpation of taut muscles, area with severe tenderness was identified by repeated palpations, painful expression on the patient’s face produced with pressure on the tender areas, a “jump and shout” response, and the radiation of pain to a distant area indicated the location of trigger points  **Exclusion**: fibromyalgia syndrome, cervical disc lesion, radiculopathy, kyphoscoliosis, myelopathy, recent trigger point injection, inflammatory musculoskeletal system disorder, history of psychiatric and systemic disorders, bleeding diathesis, pregnant women, history of brain or shoulder surgery, inability to cooperate, participated in a physical therapy program for myofascial pain within the past six months | N = 55 | **T_1_**: KT  (n=27)  **T_2_**: Dry needling  (n=28) | **Pain**:  VAS  **Disability**:  NDI  **Functional**:  Nottingham health profile (NHP)  **Time points**: 2 week intervention   1. Baseline 2. 4 weeks 3. 12 weeks | **Pain**:  Both groups improved at week 4 and week 12, however, no significant differences were found between dry needling and KT with regard to the VAS scores measured at weeks 0, 4, and 12  **Disability**:  NDI scores measured at weeks 4 and 12 were statistically significant favoring dry needling group  **Functional:**  NHP scores measured at weeks 4 and 12 were statistically significant favoring dry needling group | Pain can be reduced comparably by both dry needling and kinesiotaping, however, disability and function are more remarkably improved by dry needling.  Both dry needling and kinesiotaping can provide an increasing effectiveness up to 12 weeks. | 6 |
| Kavlak  (2012) [165] | **Age**: 18-70  **Diagnosis**: neck pain  **Inclusion**: cervical disc herniation, cervical spondylosis, or cervical radiculopathy, acute subacute or chronic pain  **Exclusion**: physical therapy in the previous month, neck pain due to fracture, infection, or non-mechanical reasons, history of cervical spine surgery, inflammatory pathologies, rheumatoid arthritis, progressive neurological deficit, myelopathy | N=60 | **T_1_**: Classic therapy (n=20)  **T_2_**: Classic therapy plus mobilization  (n=20)  **T_3_**: Classic therapy plus KT  (n=20) | **Pain**:  VAS  **Disability**:  NDI  **Functional**: n/a  **Time points**: 3+ week intervention  1. Baseline  2. Post treatment | **Pain:**  No significant difference was detected among the  groups for VAS  **Disability**:  No significant difference was detected among the  groups for NDI | KT and mobilization may be helpful as an alternative treatment in neck pain. | 6 |
| Kilinc  (2015) [166] | **Age**: 18-50  **Diagnosis**: mechanical neck pain  **Inclusion**: pain >3 months, NDI >5 points  **Exclusion**: neck surgery, traumatic spinal cord injury, radiculopathy, myelopathy (sensory or motor deficit), neurological or rheumatologic disease and structural scoliosis | N = 31 | **T_1_**: scapular mobilization, ischemic compressions, Cyriax mobilization techniques, cervical manual traction  (n=14)  **T_2_**: T_1_ and KT (n=14) | **Pain**:  VAS for headache and neck pain  **Disability**:  NDI  Beck Depression Questionnaire (BDQ)  **Functional**:  Craniocervical Flexion Test (CCFT)  **Time points**: 45 minutes intervention   1. Baseline 2. 4 days | **Pain:**  No significant difference between groups for improvement in intensity of headache and neck pain-free duration or pre and post-test levels  **Disability**:  NDI and BDQ used for pre-assessment only  **Functional**:  No significant difference between groups on improvement in CCFT | KT application had no additional effect to the mobilization techniques on decreasing the neck pain, headache intensity and enhancing the activation of deep cervical neck flexor muscles in mechanical neck problems. | 5 |
| Kim  (2018) [167] | **Age**: not specifically described    **Diagnosis**: Forward head posture  **Inclusion**: center of the ears is positioned more than 2.5 cm anterior to the center of the shoulders, NDI of 14 or lower  **Exclusion**: cervical fracture, neuromyopathy. inflammatory disease, history of disc surgery < 3, vascular diseases, psychiatric problems that would hinder the comprehension of the survey content | N = 29 | **T_1_**: McKenzie and myofascial release  (n=10)  **T_2_**: McKenzie and KT  (n=10)  **T_2_**: McKenzie, myofascial release and KT  (n=9) | **Pain**: n/a  **Disability**:  NDI  **Functional**:  Craniovertebral angle  Cranial rotation angle  **Time points**: 4 week intervention duration   1. Baseline 2. Post treatment | **Disability**:  Post-treatment outcome was not reported  **Functional**:  The combined treatment group showed a significant change of craniovereterbral angle.    All three groups showed changes of cranial rotation angle post-treatment, but was not statistically significant | McKenzie exercise, KT, and myofascial release improved participants’ forward head posture, and the effects were significantly greater in the group that underwent all three interventions. | 5 |
| Öztürk (2016) [177] | **Age**: 18-50  **Diagnosis**: myofascial trigger points of upper trapezius  **Inclusion**: neck and/or upper back pain with active myofascial trigger point in the upper trapezius  Region, diagnosed according to the diagnostic criteria of Travell and Simon, symptom duration > 2 weeks  **Exclusion**: fibromyalgia syndrome, psychiatric disorders, symptoms of radiculopathy, brachial plexopathy or other nerve entrapments, treatment in last 6 months for myofascial pain, malignancy, pregnancy, infectious disease, inflammatory musculoskeletal disease, prior shoulder or neck surgery | N = 37 | **T_1_**: KT  (n=20)  **T_2_**: Sham KT  (n=17) | **Pain**:  VAS  PPT  **Disability:** n/a  **Functional**:  Muscle Strength  **Time points**: 4 week intervention   1. Baseline 2. Post-treatment 3. 1 month | **Pain**:  VAS and PPT were significantly different between groups at 1 month compared with baseline in favor of KT  **Functional:**  Trapezius muscle strength was significantly different between the groups at post-treatment compared to baseline in favor of KT | The application of KT to the trapezius muscle resulted in a significant improvement in the pain level post-treatment and at 1 month follow-up.  Applying KT along the trapezius area also increased trapezius muscle strength at 1 month after KT application. | 3 |
| Ptaszkowski (2015) [178] | **Age**: 18 and older  **Diagnosis**: upper trapezius pain  **Inclusion**: pain and limited cervical range of motion during forward flexion and lateral flexion to the side opposite to the involved upper trapezius muscle, increased upper trapezius muscle tone (palpation assessment), the lack of skin allergies  **Exclusion**: history of upper limb, back, or neck severe injury or medical intervention over the last 5 years (fracture, surgical intervention, and dislocation), peripheral or central nervous system neurological disease, pharmacological treatment at present, chronic headache, open wound, rash, infection, a pacemaker, or epilepsy | N = 52 | **T_1_**: Post isometric relaxation  (n=26)  **T_2_**: KT  (n=26) | **Pain**:  VAS  **Disability**:  n/a  **Functional**: sEMG activity for upper trapezius  **Time points**: 24 hours intervention   1. Baseline 2. 24 hours | **Pain**:  In post isometric relaxation group, the average VAS result decreased by 0.7 points from pre-intervention to post-intervention. Significant differences were found in KT group, and the average VAS result decreased by 2.0 points  **Disability**:  Significant differences were found in KT group,  and the average resting bioelectrical activity  No significant differences were found between pre- and post-intervention results in post isometric relaxation group  **Functional**:  No difference in sEMG findings between groups | Post isometric relaxation method did not change the sEMG activity and VAS score. However, KT application slightly decreased bioelectrical activity of the upper trapezius muscle as well as pain perception. | 4 |
| Puerma-Castillo  (2017) [168] | Age: 18-55  Diagnosis: mechanical neck pain  Inclusion: pain in situations of stretching, touching, movement, contraction against resistance or after holding the same position for a period of time, VAS ≥ 50 mm, asymmetrical lateral flexion of the neck of > 5 degrees  Exclusion: neck pain due to whiplash or direct trauma, prior neck or shoulder surgery, fibromyalgia, radiculopathy, cervical osteoarthritis, involvement in legal suit, physical therapy treatment including KT in last year, pain > 3 months, allergies to bandage or adhesive material | N = 42 | **T_1_**: KT and rehab  (n=13)  **T_2_**: Placebo and rehab  (n=15)  **C**: Rehab (postural education and cervical exercises)  (n=12) | **Pain**:  VAS  **Disability**:  Short Form 36  **Functional**: Medication Intake  Cervical ROM  **Time points:**   1. Baseline 2. After bandage application 3. 24 hours post bandage application 4. 4 days post bandage application 5. 1 month follow-up | **Pain**:  No significant differences between groups  **Disability**:  No significant differences between groups  **Functional**:  Between-group analyses detected no significant differences across groups at any of the six measurement times  Significant long-term improvements in neck flexion in all three treatment groups, when comparing range of flexion at baseline and at one-month follow-up | No evidence of additional benefits from the use of KT in this population | 8 |
| Rasti  (2018) [179] | **Age**: 18 and older  **Diagnosis**: myofascial trigger points of upper trapezius  **Inclusion**: symptoms > 2 weeks, at least one myofascial trigger point in the upper trapezius (diagnosis: Touching the taut bands or sensitive point, detecting the patient’s pain with soft tissue traction, normal neurological tests, the slow or  deep pain that gets worse due to stress)  **Exclusion**: fibromyalgia, medication for pain, musculoskeletal disease, neurological disease, inflammatory diseases, allergy to KT | N = 30 | **T_1_**: KT (tension0  (n=15)  **C**: KT (no tension)  (n=15) | **Pain**:  NPRS  **Disability**:  NDI  **Functional**:  ROM  **Time points**: 2 week intervention   1. Baseline 2. 3 days 3. Post-treatment | **Pain**:  A significant difference was observed between the two groups post-treatment and in 2 weeks favoring KT with tension.  **Disability**:  A significant difference was observed in the treatment groups in 2 week in favor of KT with tension    **Functional:**  A significant difference between the two groups in flexion, rotation, and lateral flexion following treatment and in 2 weeks | KT can improve neck pain, increase cervical ROM, and  reduce the disability caused by myofascial pain syndrome both post-treatment and in 2 weeks. However, the duration of the use of KT can increase its influence. | 5 |
| Saavedra-Hernández  (2012) [169] | **Age**: 18-55  **Diagnosis**: mechanical idiopathic neck pain  **Inclusion**: generalized neck or shoulder pain provoked by sustained neck postures, neck movement, or palpation of the cervical musculature  **Exclusion**: contraindication to neck manipulation, history of whiplash, history of cervical surgery, cervical radiculopathy or myelopathy, fibromyalgia syndrome, previous spinal manipulation therapy or KT applications, tape allergy | N = 80 | **T_1_**: KT  (n=40)  **T_2_**: cervical thrust manipulation  (n=40) | **Pain**:  NPRS  **Disability**:  NDI  **Functional**: Cervical ROM  **Time points:**   1. Baseline 2. 7 days | **Pain**:  Decreases in pain were similar in both groups  **Disability**:  Changes in disability slightly less than the minimum clinically important difference.  **Functional**:  Cervical ROM changes small and not clinically meaningful. | KT and cervical thrust manipulation both lead to similar decreases in pain and disability and increases in CROM. The effects on pain are small, but positive. There was no control group included so effects could be due to placebo or simply time passing. | 8 |
| Sobhani (2017) [170] | **Age**: not specifically described  **Diagnosis**: chronic neck pain    **Inclusion**: ale, bilateral cervical myofascial pain involving upper trapezius and levator scapulae muscles, pain duration ≥3 months, VAS pain intensity ≥ 2 out of 10, symptoms of neck pain provoked  either by neck postures or neck motions, NDI ≥ 15 points, cervical ROM restriction, myofascial trigger points of upper trapezius and levator scapulae muscles.  **Exclusion**: Manipulation application contraindication, orofacial pain or temporomandibular joint disorders,  history of traumatic injuries (i.e. contusions, fractures), systemic diseases (fibromyalgia and psori-  atic arthritis), neurological diseases, presence of concomitant to headache prior cervical surgery, cervical radiculopathy or myelopathy, unilateral neck pain, needle phobia, history of skin irritability, previous history of receiving physical therapy, KT or manipulation in the past 6 months | N = 39 | **T_1_**: Dry needling  (n=13)  **T_2_**: Manual therapy  (n=13)  **T_3_**: KT  (n=13) | **Pain**:  VAS  **Disability**:  NDI  Pain Catastrophizing Scale (PCS)  **Functional**: Cervical ROM  **Time points**: 10 day intervention duration   1. Baseline 2. Post treatment | **Pain**:  Pain intensity significantly improved in all groups  **Disability**:  PCS and NDI significantly improved in all groups  **Functional**: Cervical ROM in all 6 directions significantly improved in all groups  Manual therapy outperformed KT and dry needling, however no differences between KT and dry needling | Dry needling, manual therapy, and KT each improve pain and neck disability and increase cervical ROM in patients with myofascial  Chronic neck pain.  The manual therapy techniques are more effective in increasing cervical ROM for rotation compared to the other 2 methods. | 5 |

**APPENDIX 2J: EVIDENCE TABLE FOR RANDOMIZED CONTROLLED TRIALS OF TEMPOROMANDIBULAR JOINT DISORDERS**

| Study | Population description | Sample size | Interventions | Outcomes/time points | Results | Key author conclusions | PEDro Score |
| --- | --- | --- | --- | --- | --- | --- | --- |
| Bae  (2014) [181] | **Age**: 20 – 30  **Diagnosis**: temporomandibular joint myofascial pain  **Inclusion**: presence of any latent trigger point (palpable taut bands or nodules present in the skeletal muscles, hypersensitive tender spots present in the taut bands in the skeletal muscles) in the left or right unilateral sternocleidomastoid muscle, restriction of the ROM of the temporomandibular joint, crepitation or inconvenience during mastication  **Exclusion**: trauma, any latent trigger point caused by the same trauma, restriction of the ROM of the temporomandibular joint caused by any systematic disease such as arthritis, and treatment to release the sternocleidomastoid muscle or to relieve pain within several months before the time of examination | N = 42 | **T_1_**: KT  (n=22)  **C**: No intervention  (n=23) | **Pain:**  VAS  **Disability**:  n/a  **Functional**:  Pressure pain threshold  ROM  **Time points**: 4 weeks intervention   1. Baseline 2. Post treatment | **Disability**:  VAS score decreased significantly in the KT group  **Functional**:  Pressure pain threshold score decreased significantly in the KT group. ROM increased significantly in the KT group | Applying KT to the sternocleidomastoid muscle, the myofascial pain was relieved and the ROM of the temporomandibular joint increased. | 4 |
| Coskun  (2016) [182] | **Age**: <55  **Diagnosis**: temporomandibular joint disorder  **Inclusion**: patients diagnosed with myofascial pain, arthralgia, and/or disc displacement with reduction according to the Research Diagnostic Criteria for Temporomandibular Disorders by a dentist  **Exclusion**: history of any surgical procedures of the temporomandibular joint including arthrosynthesis and arthroscopy, inflammatory joint disease such as ankylosing spondylitis and rheumatoid arthritis, history of trauma to the jaw, > 55 years of age, any known tape allergy, any reason of orofacial pain other than  temporomandibular joint disorder | N = 28 | **T_1_**: KT + exercise and counseling  (n=14)  **C**: Exercise and counseling  (n=14) | **Pain:**  VAS at rest, on palpation  **Disability**:  Biobehavioral Questionnaire  Likert scale for functional limitation  Likert scale for masticatory efficiency  **Functional**:  ROM  **Time points**: 6 weeks intervention   1. Baseline 2. 1 week 3. Post treatment | **Pain:**  Although the reduction in VAS from baseline to sixth week was significantly higher in the KT group than that in controls, there was no statistically significant improvement from week 1 to week 6, in both groups  **Disability**:  No significant improvement from baseline was observed in controls. Improvement in the experimental group at first week was significantly favorable  **Functional**:  Change in baseline active mouth opening was significantly higher in the KT group, on the first and sixth week. Right laterotrusion improvement was achieved by time in the KT group, but not in controls. For left laterotrusion, both groups showed similar improvements from baseline to both first and sixth week | KT in combination with counseling and masticatory muscle exercises has additional benefit in relieving pain, improving disability and increasing the mobility of temporomandibular joint than counseling and exercise alone.  KT might serve as an alternative  and/or an adjuvant approach in the management of temporomandibular disorders. | 5 |
| Keskinruzgar (2019) [183] | **Age**: not specifically described  **Diagnosis**: Myofascial pain of temporomandibular joint (bruxism)    **Inclusion**: criteria that included a combination of the following: clenching or grinding of teeth for at ≥ 5 days a week for ≥ 6 months, someone comments teeth grinding during sleeping period, presence of shiny dental restorations, dental abrasion, hypertrophy in masseter muscles and palpation-induced pain in these muscles  **Exclusion**: poor treatment compliance, systemic disease, surgical history in the maxillofacial area, previously treated for bruxism | N = 34 | **T_1_**: KT  (n=16)  **T_2_**: Occlusal splint  (n=18) | **Pain**:  VAS  PPT  **Disability**: n/a  **Functional**: Mouth ROM  **Time points**: 5 week intervention duration   1. Baseline 2. 1 week 3. 5 week | **Pain**:  Both interventions improved significantly from baseline at week 1 and week 5, however, there was no statistical difference between interventions at any time points, except temporal PPT values in the KT group was significantly  **Functional**:  Both interventions improved significantly from baseline at week 1 and week 5, however, there was no statistical difference between interventions at any time points | KT is an easy and reliable treatment  method that reduces muscle pain and increases mouth  opening size in bruxism patients.  KT is at least as effective as occlusal splint for the treatment of sleep bruxism. | 5 |
| Lietz-Kijak (2018) [184] | **Age**: 18-35  **Diagnosis**: Temporomandibular myofascial trigger points    **Inclusion**: research diagnostic criteria for temporomandibular disorders by Dworkin and LeResche  **Exclusion**: regular drug therapy, mental illness, coagulopathy, diabetes, or chronic infections. addicted to nicotine, alcohol, or drugs, clinical diagnosis of disc displacement accompanied with joint clicking | N = 60 | **T_1_**: KT  (n=30)  **T_2_**: ischemic compression  (n=30) | **Pain**:  VAS  **Disability**: n/a  **Functional**: n/a  **Time points**: 5 day intervention duration   1. Baseline 2. Post treatment | **Pain**:  Both groups proved to be efficacious, though,  KT gives greater improvement in the reduction of pain | KT and ischemic compression demonstrated significant analgesic effects in the treatment of functional disorders of the masticatory muscles.  More beneficial pain reduction was observed in the KT group. | 4 |

**APPENDIX 2K: EVIDENCE TABLE FOR RANDOMIZED CONTROLLED TRIALS OF MUSCULOSKELETAL CONDITIONS NOT OTHERWISE CLASSIFIED**

| Study | Population description | Sample size | Interventions | Outcomes/time points | Results | Key author conclusions | PEDro Score |
| --- | --- | --- | --- | --- | --- | --- | --- |
| Aleksiev (2013) [187] | **Age**: 18 and older  **Diagnosis**: myofascial pain syndrome  **Inclusion**: myofascial pain due  to muscle spasm and shortening of static muscles, resulting in muscle imbalance in one of the following  8 kinetic segments: hand; forearm; arm, shoulder girdle; foot; leg; thigh; spine.  **Exclusion**: neurologic deficits, surgery during the previous year, structural abnormalities, osteoporosis, severe infections or decompensation (cardiovascular, pulmonary, hepatic, renal, etc.). | N = 320 | **T_1_**: Post-isometric relaxation (PIR)  (n=160)  **T_2_**: PIR and KT (n=160) | **Pain**:  VAS  **Disability**: n/a  **Functional**: n/a  **Time points**: 12 day intervention   1. Baseline 2. 4th day 3. 8th day 4. Post treatment | **Pain**:  Both PIR and PIR-taping resulted in a decrease in pain that was statistically significant, but the differences were not significant. PIR-taping decreased pain on weekends, whereas PIR alone increased pain on weekends | PIR displayed better short-term effects than PIR-taping, but the extended period of PIR-taping effects results in no statistically significant differences between groups | 3 |
| Lee  (2012) [186] | **Age**: 69-83  **Diagnosis**: myofascial pain syndrome  **Inclusion**: 3 of Simons’ 4 diagnostic criteria for myofascial pain syndrome  **Exclusion**: no specific description | N = 32 | **T_1_**: stabilization exercises  (n= 16)  **T_2_**: non-elastic taping and stabilization exercises  (n= 16) | **Pain**:  VAS  Pain pressure threshold  **Disability**: n/a  **Functional**: Constant-Murley scale  **Time points**: 4 week intervention   1. Baseline 2. Post treatment | **Pain**:  The changes in VAS and PPT  were statistically significant in both groups, however, comparison of VAS and PPT changes between the groups did not show any significant differences  **Functional**:  Stabilization group showed significant differences in all items except for ability to perform ADLs, while the taping group showed statistically significant differences in all items | Compared to the stabilization  exercise group, the taping group showed larger pain  relief effects and greater improvement in ability to perform  activities of daily living. | 5 |
| Palmer (2018) [188] | **Age**: not specifically described  **Diagnosis**: osteoporotic vertebral fractures    **Inclusion**: back pain concurrent with a diagnosis of osteoporotic vertebral fracture confirmed by a Rheumatologist, fracture not immobilised, independently mobile (with or without an aid), capable of applying the postural taping device to the low back  **Exclusion**: osteoporosis secondary to metabolic bone disorders, fragile or broken skin, allergy to adhesive plasters, vertebroplasty or kyphoplasty | N = 24 | **T_1_**: rigid tape (PosturePlast device)  (n=13)  **C**: usual care  (n=11) | **Pain**:  VAS (rest, movement)  **Disability**: n/a  **Functional**: Quality of Life Questionnaire of the European Foundation for Osteoporosis (QUALEFFO-41)  **Time points**: 4 week intervention duration   1. Baseline 2. Post treatment | **Pain**:  Mean difference favours taping  **Functional**:  Mean difference favours taping in with the exception of the QUALEFFO-41 general health perception subscale | The postural taping device has demonstrated the potential to improve pain, function and quality of life.  A more extensive pilot RCT with longer follow-up is recommended prior to a definitive trial. | 6 |

Abbreviations

Activities of Daily Living - ADL

American Shoulder and Elbow Surgeons Evaluation - ASESS-100

Boston Carpal Tunnel Syndrome Questionnaire - BCTSQ

Back Depression Inventory - BDI

Back Pain Function Scale - BPFS

Disabilities of the Arm,Shoulder and Hand - DASH

Extracorporeal shock wave therapy - ESWT

Electroneuromyography - ENMG

Kinesio tape - KT

Medical Research Council Shoulder strength assessment - MRC

Neck Disability Index -NDI

Mobilization with Movement - MWM

Non-steroidal anti-inflammatory drugs - NSAIDs

Numeric Pain Rating Scale - NPRS

Numeric Rating Scale - NRS

Oswestry Disability Index - ODI

Oxford Shoulder Score - OSS

Pain Catastrophizing Scale - PCS

Pain Pressure Threshold - PPT

Patient-rated tennis elbow evaluation - PRTEE

Post-isometric relaxation - PIR
Quality of Life Questionnaire of the European Foundation for Osteoporosis - QUALEFFO-41

Range of Motion - ROM

revised Oswestry Disability Index - rODI

Roland Morris Disability Questionnaire - RMDQ

36-item Short Form Survey- SF-36

Subjective Shoulder Rating Questionnaire - SSRQ

Timed Up and Go test - TUG

Visual Analog Scale - VAS

Western Ontario Rotator Cuff Index - WORC

Western Ontario and McMaster Universities Arthritis Index - WOMAC
